# The Synthesis of 3-(*R*)- and 3-(*S*)-Hydroxyeicosapentaenoic Acid

**DOI:** 10.3390/molecules27072295

**Published:** 2022-04-01

**Authors:** Gard Gjessing, Lars-Inge Gammelsæter Johnsen, Simen Gjelseth Antonsen, Jens M. J. Nolsøe, Yngve Stenstrøm, Trond Vidar Hansen

**Affiliations:** 1 Department of Chemistry, Biotechnology and Food Science, Norwegian University of Life Sciences, P.O. Box 5003, NO-1433 Ås, Norway; gjegar@gmail.com (G.G.); simen.antonsen@oslomet.no (S.G.A.); jens.mj.nolsoe@nmbu.no (J.M.J.N.); 2 Section of Pharmaceutical Chemistry, Department of Pharmacy, University of Oslo, P.O. Box 1068 Blindern, NO-0316 Oslo, Norway; lars.inge1988@gmail.com; 3 Department of Mechanical, Electronic and Chemical Engineering, Faculty of Technology, Art and Design, OsloMet, P.O. Box 4, St. Olavs Plass, NO-0130 Oslo, Norway

**Keywords:** 3-hydroxy eicosapentaenoic acid, eicosanoids, Nagao–Fujita acetate aldol reaction, Braun acetate aldol reaction, eicosapentaenoic acid, docosahexaenoic acid, oxylipins, ^19^F NMR Mosher esters analysis, stereoselective synthesis

## Abstract

Monohydroxylated polyunsaturated fatty acids belonging to the oxylipin class of natural products are present in marine and terrestrial sources as well as in the human body. Due to their biological activities and role in diverse biosynthetic pathways, oxylipins biosynthesized from eicosapentaenoic acid and arachidonic acid have attracted great interest from the scientific community. One example is 3-hydroxyeicosapentaenoic acid where the absolute configuration at C-3 has only been tentatively assigned. In this paper, studies on acetate type aldol reactions that enabled the preparation of 3-(*R*)-hydroxyeicosapentaenoic acid (3R-HETE, **2**) and its enantiomer are presented.

## 1. Introduction

**Figure 1:**
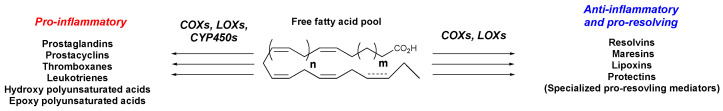
An outline of the different classes of oxylipins.

Biologically active oxygenated natural products derived from the two ω-3 polyunsaturated fatty acids (PUFAs), eicosapentaenoic acid and arachidonic acid, have for a long time been the topic of biological and biosynthetic investigations [1,2], but also targets for stereoselective total synthesis [3]. Examples of classes of such natural products are prostaglandins [4], leukotrienes [5], lipoxins [6], resolvins, maresins, protectins [7], epoxy fatty acids [8] and oxylipins [9]. These natural products are biosynthesized by three distinct main pathways where cytochrome P450 oxidases or various enzymes of the cyclooxygenase, lipoxygenase and epoxygenase classes are involved (Figure 1). Very often these biosynthetic pathways involve the formation of a mono-hydroxy substituted intermediate that is further converted to the aforementioned oxylipin classes [5,7].

**Figure 2:**
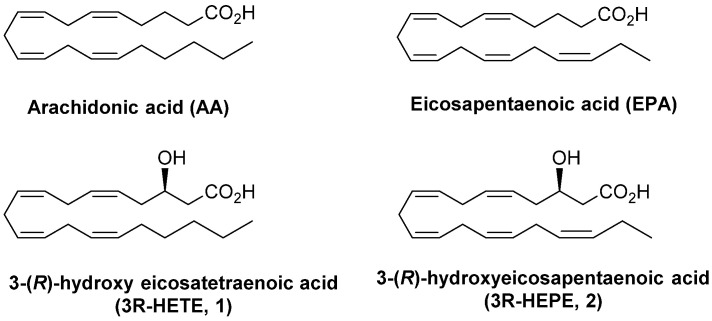
Chemical structures of (*R*)-3-hydroxy arachidonic acid (**1**) and (*R*)-3-hydroxyeicosapentaenoic acid (**2**) with their putative biosynthetic starting materials.

To date, numerous C-6 to C-20 fatty acids with a hydroxy substituent in the C-3 position have been reported isolated from several fungi [10]. However, only two mono-hydroxy oxylipins substituted in the 3-position derived from the PUFA eicosapentaenoic acid and arachidonic acid have been reported (see Figure 2) [10,11]. The first one to be reported was 3-(*R*)-hydroxy arachidonic acid (3R-HETE, **1**) [11,12], while 3-(*R*)-hydroxyeicosapentaenoic acid (3R-HEPE, **2**) was reported to have been isolated from the yeast *Dipodascopsis uninucleata* [13,14]. To date, only the oxylipin **1** has been synthesized [15,16] and subjected to biological investigations [11,14].

To date, limited knowledge with respect to the role of the oxylipin **2** constitutes in microbial pathogenesis is available. In order to gain new knowledge of the biological processes mediated by this oxylipin, biosynthetic and biological studies are needed. However, such endeavors require access to sufficient amounts of chemically pure EPA-derived oxylipin **2**, not easily available by isolation from biological sources. Moreover, the structural elucidations of these lipids have been based on GC/MS- and HPLC-analyses [13,17], due to the minute amount isolated from fungal sources. Hence, stereoselective synthesis is in demand in order to establish the tentatively assigned *R*-configuration of the PUFA derived **2**. Furthermore, the need for new antifungal drugs is imminent [18].

Based on the above arguments, we aim for a convenient and stereoselective synthesis of (*R*)-3-hydroxyeicosapentaenoic acid (**2**). In order to meet this aim, the four all-*Z* double bonds present in **2** called for docosahexaenoic acid (**3**) as a convenient starting material, in particular based on our prior successful experiences [19,20]. This approach keeps the all-*Z* methylene interrupted double bonds present in **3**, biosynthesized by highly specific desaturases, intact throughout the synthesis of **2**. 

The C18 aldehyde all-*Z*-(3,6,9,12,15)-octadeca-(3*Z*,6*Z*,9*Z*,12*Z*,15*Z*)-pentaenal (**4**) constitutes a useful starting material in order to meet these needs. Regarding the chiral center of the homoallylic alcohol present in **2**, we noted that the absence of any large stereo-directing groups and the vast number of possible conformers of **2** limited as well as directed our retrosynthetic analyses towards diastereoselective acetate type aldol reactions. The Nagao–Fujita reaction [21] allows the use of different thiazolidinedione auxiliaries to participate in aldol reactions with a range of different aldehydes, including aliphatic ones. Early reports were on the tin-mediated aldol addition of various substituted *N*-acetyl-1,3-thiazolidine-2-thione (**5a–5e**) to a wide range of aldehydes [21,22]. 

Later, Urpí and Vilarrasa expanded this methodology to titanium enolates using different bases, where diisopropylamine (DIPEA) gave high diastereoselection [23], also studied by Crimmins and co-workers with aliphatic aldehydes [24,25]. Moreover, Hodge and Olivo investigated the combined use of (−)-sparteine and titanium enolates of the *iso*-propyl substituted auxiliary **5b**, which afforded high diastereoinduction of aliphatic aldehydes [26]. In this paper, we report our results using the Nagao–Fujita reaction under various conditions with aldehyde **4** that enabled the first total synthesis of 3-(*R*)-hydroxyeicosapentaenoic acid (3R-HEPE, **2**). In addition, performing the Braun aldol reaction [27] on the same aldehyde, an efficient preparation of the enantiomer of **2** became available.

## 2. Results and Discussion

**Scheme 1:**
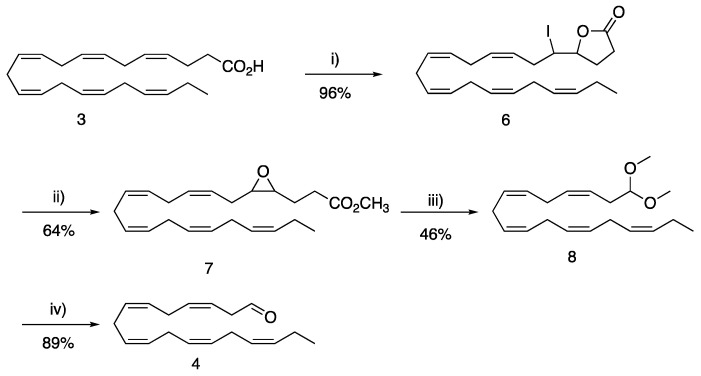
Preparation of chemically pure **4** starting from docosahexaenoic acid (**3**). (i) 2,5-lutidine, I_2,_ CH_2_Cl_2_, 0 °C, 15 h; (ii) K_2_CO_3,_ MeOH, rt, 3 h; (iii) H_5_IO_6_, MeOH, rt, 6 h; (iv) HCO_2_H, dioxane, rt, 1.5 h.

The preparation of aldehyde **4** was achieved as presented in Scheme 1 using docosahexaenoic acid (**3**) as the starting material. First, a modification of the Corey-lactonization protocol [28,29] was used for the preparation of δ-lactone **6** in 96% yield that was conveniently converted into epoxy methyl ester **7** in 64% yield. Reacting **7** with H_5_IO_6_ and MeOH afforded the acetal **8** in a 46% yield that was used for long-term storage, while the more sensitive aldehyde **4** was formed by treatment with formic acid in dioxane in 89% overall yield from **8**. The conservation of the five *all*-*Z* methylene interrupted double bonds in **4** was confirmed by ^13^C NMR spectroscopy, since no characteristic signals for allylic carbons from *E,Z*-conjugated isomers were present within the limits of detection [30].

**Scheme 2:**
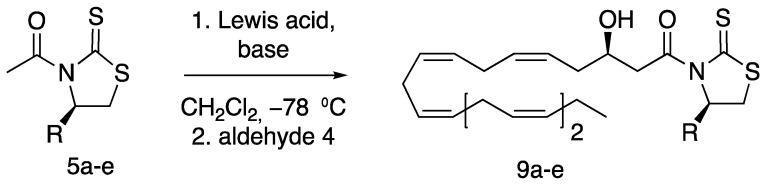
Investigations on the Nagao–Fujita acetate aldol reaction.

Next, the Nagao–Fujita reaction was investigated (see Scheme 2) with the aldehyde **4**. The results are compiled in Table 1.

Variable stereoinduction was observed with the different auxiliaries **5a**–**5e** with TiCl_4_ and DIPEA (Entries 1–5), giving reasonable yields of the desired acetate *syn* [26] and *R*-configured products **9a**–**9d**, Scheme 2. No reaction was observed with 4-phenyl-2-thioxothiazolidine (**5e**), entry 6, while the methyl **5a** and the *tert*-butyl **5c** substituted auxiliaries both gave low stereoinduction (entry 1 and 4). The benzyl substituted thiazolidine **5d** gave a modest selectivity (entry 5). The best result with respect to diastereoselectivity (dr = 10:1) of the desired product **9b** was obtained with *N*-acetyl-4-isopropyl-1,3-thiazolidine-2-thione (**5b**), but the yield was moderate (46%) (entry 2). A similar yield was observed when using *N*-methyl-2-pyrrolidinone as solvent [22], but no selectivity was observed (entry 3). Of note, the conditions reported by Hodge and Olivo afforded a 5.3:1 ratio of the desired *R*-configured product **9b** (entry 7). Using the conditions (Sn(OTf)_2_ and 1-ethyl piperidine) reported by Nagao and co-workers [21] did not yield any of the desired aldol products **9a**–**9e [26]**.

Chromatographic purification by silica gel chromatography enabled the isolation of the major acetate *syn* aldol product of **9b**. The assignment of the chiral center of the *syn R*-configured aldol product **9b** was based on the arguments by Hodge and Olivo [26], by the typical chemical shift values for the alpha protons at 3.64 ppm (dd, *J* = 17.7, 2.6 Hz, 1 H) and at 3.51 ppm (dd, *J* = 11.5, 7.9 Hz, 1 H), for the less and the more shielded protons, respectively.

**Scheme 3:**
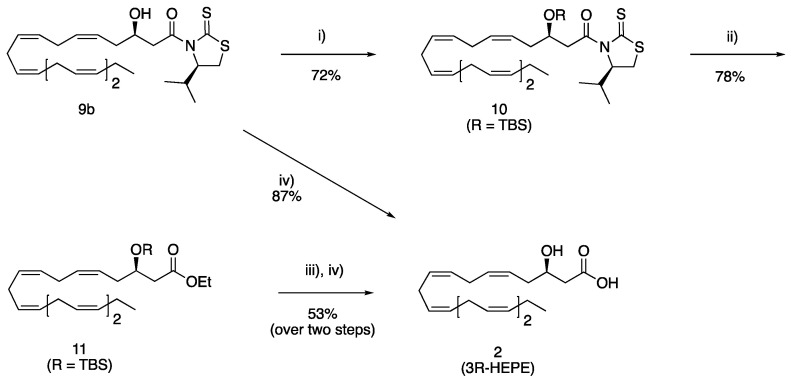
Synthesis of 3-(*R*)-hydroxyeicosapentaenoic acid (3R-HEPE, **2**). (i) TBSOTf, 2,6-lutidine, CH_2_Cl_2_, −78 °C; (ii) K_2_CO_3_, EtOH, 0 °C; (iii) TBAF, THF, 0 °C; (iv) LiOH, THF, MeOH, H_2_O, 0 °C.

Next, reaction conditions to obtain the desired product **2** were attempted. However, the long-time storage of the aldol product **9b** proved problematic, as it decomposed and formed its α, β-unsaturated isomer over time. Hence, TBS-protection was performed, which yielded **10** in 72% yield that was cleanly converted to the ethyl ester **11** in 78% yield. Then, **13** was converted using a two-step procedure (TBS-deprotection, hydrolysis, 53% yield) to afford **2** (see Scheme 3). The spectroscopic data (UV, IR, and NMR) were all in accord with the assigned structure and the MS-data were comparable to the literature [13]. Later, the treatment of **9b** with diluted aqueous LiOH in THF and MeOH cleanly afforded (*R*)-3-hydroxyeicosapentaenoic acid (**2**) in 87% isolated yield (Scheme 3) [31]. The shortest sequence presented in Scheme 1, Scheme 2 and Scheme 3 afforded **2** in 10% overall yield over the six steps.

**Scheme 4:**
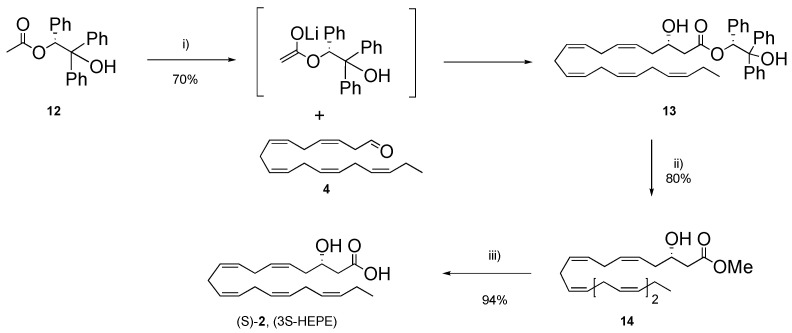
Synthesis of 3-(*S*)-hydroxyeicosapentaenoic acid (3S-HEPE, **2**) using the Braun reaction. (i) LDA, THF, −78 °C; (ii) K_2_HPO_4_, MeOH, Δ; (iii) LiOH, THF, MeOH, H_2_O, 0 °C.

Then, we attempted the method reported by Braun and co-workers [27] using (*R*)-2-hydroxy-1,2,2-triphenylethyl acetate (**12**) (see Scheme 4). In this case, the diastereomeric ratio was very high (95:5), and **13** was isolated in 70% yield after chromatographic purification. Hydrolysis with K_2_HPO_4_ in refluxing MeOH cleanly removed the ester auxiliary in **13** to give the methyl ester **14**. Hydrolysis afforded the *S* enantiomer of 3R-HEPE, namely (*S*)-3-hydroxyeicosapentaenoic acid (**2**). The spectral data matched those of synthetic R-**2**. The optical rotation was observed to be dextrorotary (${\left[\alpha \right]}_{D}^{20}$ = + 11.1, CHCl_3_, c 0.90), while (*R*)-3-hydroxyeicosapentaenoic acid gave a levorotary rotation (${\left[\alpha \right]}_{D}^{20}$ = −10.5°, CHCl_3_, c 1.08). Starting from DHA, the overall yield of *S*-**2** was 12% over the eight steps. Hence, both enantiomers were made available for biological and biosynthetic investigations.

To prove the absolute configuration of each of the enantiomers of 3-hydrocyeicosapentaenoic acid, we produced the Mosher ester derivatives [32]. However, looking at the ^1^H NMR spectra of the two compounds, the diagnostic chemical shifts overlapped to such an extent that accurate interpretation was impossible. Fluorine NMR analysis has frequently been used to make assignments of the configuration of MTPA esters. Using Hoye and co-workers set-up [32,33], we reacted both enantiomers of **2** with both *S*- and *R*-MTBA corresponding esters (See Table 2). Looking at the presumed *R*-3-hydrocyeicosapentaenoic acid (**2**) gave a chemical shift of −72.42 ppm for the *S*-MTBA derivative and a −72.38 ppm chemical shift for the corresponding *R*-MTBA derivative. The difference, thus, is negative, implying an *R*-configuration, according to Hoye’s work and in accord with our own assumption.

A similar set-up for the presumed *S*-enantiomer of **2** gave a positive difference in chemical shift. This implies an *S*-configuration, again in accord with our assumption. The results are summarized in Table 2.

**Figure 3:**
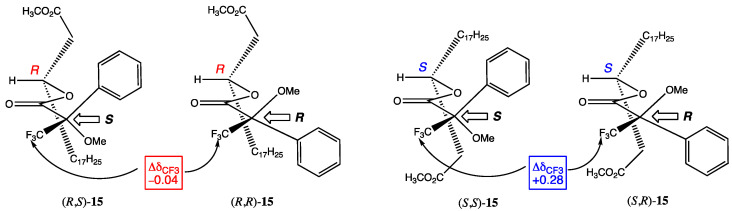
Assignment of configuration based on the Mosher ester analysis.

The trifluoromethyl group prefers the illustrated rotamers (see Figure 3), in which it is eclipsed with the carbonyl group and is, therefore, within its deshielding cone. The fundamental arguments are again based on the assumption of the preferred conformations depicted in Figure 3. The relative extent of this preference for conformers is assumed to be largely dependent upon the magnitude of the steric interaction between the large phenyl group and the carbinol substituents L2 and L3. For example, if L3 is larger than L2, the destabilizing L3, or phenyl, the interaction in the conformer of the 8-MTPA ester **15** will result in the trifluoromethyl group spending less time in the carbonyl deshielding plane (relative to other conformers for the (*R*)-MTPA ester **15**). 

Kakisawa and Kashman [34] have cautioned that conclusions based on ^19^F NMR analysis are often in error and must be scrutinized with care. However, the results are consistent with both our assumptions using well-established stereoselective reactions [22,23,24,25,26] and the conclusion by Hoye and co-workers, strongly indicating that the configuration was assigned correctly.

## 3. Materials and Methods

### 3.1. General Information

Unless stated otherwise, all commercially available reagents and solvents were used in the form in which they were supplied without any further purification. The stated yields are based on isolated material. All reactions were performed under an argon atmosphere using Schlenk techniques. Thin layer chromatography was performed on silica gel 60 F254 aluminum-backed plates fabricated by Merck. Flash column chromatography was performed on silica gel 60 (40–63 µm) produced by Merck. NMR spectra were recorded on a Bruker Ascend TM 400 (Bruker, Billerica, MA, USA) or Bruker AVI600 spectrometer (Bruker, Billerica, MA, USA) at 600 MHz or 400 MHz, respectively, for ^1^H NMR and at 150 MHz or 100 MHz, respectively, for ^13^C NMR. Coupling constants (*J*) are reported in hertz and chemical shifts are reported in parts per million (δ) relative to the central residual protium solvent resonance in ^1^H NMR (CDCl_3_ = δ 7.26, DMSO-*d*_6_ = δ 2.50 and MeOD-*d*_4_ = δ 3.31) and the central carbon solvent resonance in ^13^C NMR (CDCl_3_ = δ 77.00 ppm, DMSO-*d*_6_ = δ 39.43 and MeOD-*d*_4_ = δ 49.00). ^19^F NMR experiments were run in CDCl_3_ with signals calibrated against CF_3_CH_2_OH. Mass spectra were recorded at 70 eV on Waters Prospec Q spectrometer (Waters Corporation, Milford, MA, USA) using EI, ES or CI as the methods of ionization. High resolution mass spectra were recorded on Waters Prospec Q spectrometer using EI or ES as the methods of ionization. Optical rotations were measured using a 1 mL cell with a 1.0 dm path length on a Perkin Elmer 341 polarimeter (PerkinElmer Inc., Waltham, MA, USA). IR, MS, ^1^H and ^13^C NMR spectra as well as UV and HPLC chromatograms are found in the Supplementary Material.

#### 3.1.1. 5-((3Z,6Z,9Z,12Z,15Z)-1-Iodooctadeca-3,6,9,12,15-pentaenyl)dihydro-2(3H)-furanone (**6**)

Iodolactone **6** was prepared according to a literature procedure by Ulven and co-workers [35]. 2,6-Lutidine (7.1 mL, 60.9 mmol, 2 eq.) was added dropwise to a solution of **3** (10.0 g, 30.4 mmol, 1 eq.) dissolved in CH_2_Cl_2_ (100 mL). The mixture was cooled to 0 °C and I_2_ (15.453 g, 60.9 mmol, 2 eq.) was added. The reaction continued overnight (15 h) at 0 °C. The reaction was quenched by adding saturated aqueous Na_2_S_2_O_3_ (60 mL). Ethyl acetate was used for extraction (2 × 60 mL) and the combined organic layers were washed with saturated aqueous NaH_2_PO_4_ (50 mL) and brine, and dried using Na_2_SO_4_. The solvent was removed in vacuo and the product was passed through a short silica plug (hexane/EtOAc 1:1) to afford iodolactone **6** (13.279 g) in a 96% yield. All spectroscopic and physical data were in agreement with those reported in the literature [35]. *R*_f_ = 0.28 (hexanes/EtOAc 3:1 KMnO_4_ stain); ^1^H NMR (400 MHz, CDCl_3_) δ 5.58 (m. 1H), 5.46–5.29 (m, 9H), 4.28 (dt, *J* = 7.7, 3.0 Hz, 1H), 4.13 (dt, *J* = 7.4, 3.0 Hz, 1H), 2.90–2.69 (m, 11H), 2.63–2.52 (m, 1H), 2.42 (m, 1H), 2.09 (m, 3H), 0.98 (t, *J* = 7.5 Hz, 3H). ^13^C NMR (101 MHz, CDCl_3_) δ 176.2, 132.0, 131.6, 128.8, 128.6, 128.4, 127.9, 127.9, 127.4, 127.0, 126.7, 80.7, 37.7, 34.6, 28.5, 27.3, 25.9, 25.7, 25.7, 25.6, 20.6, 14.3.

#### 3.1.2. Methyl 3-(3-((2Z,5Z,8Z,11Z,14Z)-heptadeca-2,5,8,11,14-pentaen-1-yl)oxiran-2-yl) Propanoate (**7**)

Iodolactone **6** (8.482 g, 18.66 mmol, 1 eq.) was dissolved in MeOH (60 mL) and K_2_CO_3_ (3.096 g, 22.40 mmol, 1.2 eq.) was added. The reaction mixture was stirred at ambient temperature for 3 h. Water (50 mL) was added and the aqueous phase was extracted using hexane (3 × 50 mL). The combined organic layers were dried (MgSO_4_) and the solvent removed in vacuo. Epoxide **7** was obtained as a light brown oil (6.023 g) in a 90% yield. All spectroscopic and physical data were in agreement with those reported in the literature [36]. *R*_f_ = 0.45 (hexanes/EtOAc 3:1 KMnO_4_ stain); ^1^H NMR (400 MHz, CDCl_3_) δ 5.55–5.21 (m, 10H), 3.65 (s, 3H), 3.00–2.88 (m, 2H), 2.85–2.75 (m, 8H), 2.55–2.32 (m, 3H), 2.25–2.18 (m, 1H), 2.09–1.98 (m, 2H), 1.90–1.82 (m, 1H), 1.80–1.70 (m, 1H), 0.93 (t, *J* = 7.5 Hz, 3H). ^13^C NMR (101 MHz, CDCl_3_) δ 173.1, 132.0, 130.6, 128.5, 128.4, 128.3, 127.9, 127.8, 127.7, 127.0, 124.2, 56.5, 55.9, 51.7, 31.0, 26.2, 25.8, 25.6, 25.5, 23.3, 20.5, 14.3.

#### 3.1.3. (3Z,6Z,9Z,12Z,15Z)-1,1-Dimethoxyoctadeca-3,6,9,12,15-pentaene (**8**)

Epoxide **7** (6.023 g, 16.80 mmol, 1 eq.) was dissolved in MeOH (120 mL) and periodic acid (4.595 g, 20.16 mmol, 1.2 eq.) was added. The reaction mixture was stirred for 6 h. Water (100 mL) was added and the aqueous phase was extracted (3 × 100 mL) using hexane. The combined organic layers were washed with brine and dried (MgSO_4_). The solvent was removed in vacuo. The crude product was purified by column chromatography on silica (hexane/EtOAc 95:5) to obtain dimetylacetal **8** as a colorless oil (2.086 g) in a 41% yield. All spectroscopic and physical data were in agreement with those reported in the literature [35]. *R*_f_ = 0.46 (hexanes/EtOAc 9:1 KMnO_4_ stain). ^1^H NMR (400 MHz, CDCl_3_) δ 5.54–5.24 (m, 10H), 4.37 (t, *J* = 5.8 Hz, 1H), 3.32 (s, 6H), 2.89–2.74 (m, 8H), 2.39 (ddd, *J* = 7.2, 55.9, 1.5 Hz, 2H), 2.06 (dd, *J* = 7.3, 1.3 Hz, 2H), 0.96 (t, *J* = 7.6 Hz, 3H). ^13^C NMR (101 MHz, CDCl_3_) δ 132.1, 130.3, 128.6, 128.3, 128.1, 128.0, 127.9, 127.1, 124.0, 104.1, 53.0, 31.1, 25.9, 25.7 (2C), 25.6, 20.6, 14.3.

#### 3.1.4. (3Z,6Z,9Z,12Z,15Z)-Octadeca-3,6,9,12,15-pentaenal (**4**)

Acetal **8** (1.010 g, 3.32 mmol, 1 eq.) was dissolved in 1,4-dioxane (14 mL) and an 80% (*w*/*w*) aqueous solution of formic acid (16 mL) was added. The reaction mixture was stirred for 1.5 h at ambient temperature. The reaction was quenched by the addition of water (50 mL). The aqueous layer was extracted (3 × 30 mL) with hexane. The combined organic layers were washed with an aqueous NaHCO_3_ solution (50 mL), then with brine (50 mL) and dried (MgSO_4_). The solvent was removed in vacuo and aldehyde **4** (0.771 g) was obtained as a colorless oil in a 90% yield. All spectroscopic and physical data were in agreement with those reported in the literature [35]. *R*_f_ = 0.40 (hexanes/EtOAc 9:1 KMnO_4_ stain); ^1^H NMR (400 MHz, CDCl_3_) δ 9.66 (t, *J* = 2.0 Hz, 1H), 5.42–5.30 (m, 8H), 3.21 (dt, *J* = 7.3, 1.9 Hz, 2H), 2.86–2.80 (m, 8H), 2.11–2.00 (m, 2H), 0.96 (t, *J* = 7.6 Hz, 3H). ^13^C NMR (101 MHz, CDCl_3_) δ 199.3, 133.2, 132.1, 128.9, 128.7, 128.5, 127.9 (2C), 127.2, 127.1, 118.8, 42.6, 26.1, 25.7 (2C), 25.6, 20.7, 14.4.

#### 3.1.5. (R)-1-(4-Isopropyl-2-thioxothiazolidin-3-yl) Ethan-1-one (**5b**)

(*R*)-valinol (1.010 g, 9.79 mmol, 1 eq.) was dissolved in EtOH (5 mL) and CS_2_ (2.35 mL, 39.1 mmol, 4 eq.) was added. A 2.25 M solution of KOH (17.40 mL, 39.1 mmol, 4 eq.) in 1:1 EtOH/H_2_O was added dropwise at room temperature over 20 min. The reaction mixture was stirred and heated at reflux for 72 h. After cooling, the mixture was acidified by slowly adding 0.5 M HCl (20 mL). The slightly acidic mixture was extracted with CH_2_Cl_2_ (3 × 20 mL) and the combined organic layers were concentrated in vacuo. The crude product was purified by column chromatography on silica (hexane/EtOAc 4:1) to afford **5b** as a yellow oil (1.40 g) in a 88% yield. All spectroscopic and physical data were in agreement with those reported in the literature [37]. *R*_f_ = 0.33 (hexanes/EtOAc 7:3 KMnO_4_ stain); ^1^H NMR (400 MHz, CDCl_3_) δ 8.19 (bs, 1H), 4.15–3.96 (m,1H), 3.49 (dd, *J* = 11.1, 8.2 Hz, 1H), 3.30 (dd, *J* = 11.1, 8.3 Hz, 1H), 2.04–1.89 (m, 1H), 1.02 (d, *J* = 6.8 Hz, 3H), 0.99 (d, *J* = 6.8 Hz, 3H). ^13^C NMR (101 MHz, CDCl_3_) δ 201.1, 70.1, 36.0, 32.1, 18.9, 18.3. The obtained thioxothiazolidin (1.40 g, 9.3 mmol, 1. eq.) was dissolved in dry THF (18 mL) and cooled to 0 °C. A 60% dispersion of sodium hydride in mineral oil (0.409 g, 10.2 mmol, 1.1 eq.) was dissolved in dry THF (18 mL) and the thioxothiazolidin solution was added dropwise. The mixture was stirred for 10 min at 0 °C, followed by the addition of AcCl (0.73 mL, 10.2 mmol, 1.1 eq.) and the mixture was stirred for another 10 min at the same temperature. The heterogenous solution was allowed to reach ambient temperature and stirred for 1 h. The reaction was quenched by the addition of an aqueous 5% HCl solution (15 mL) and the aqueous layer was extracted with EtOAc (3 × 20 mL). The combined organic layers were dried (Na_2_SO_4_), filtrated and the solvent was removed in vacuo. The crude product was purified by column chromatography (hexane/EtOAc 9:1) to afford the chiral auxiliary **5b** (1.70 g) as a bright yellow oil in a 90% yield. All spectroscopic and physical data were in agreement with those reported in the literature [37]. *R*_f_ = 0.23 (hexanes/EtOAc 9:1 KMnO_4_ stain); ^1^H NMR (400 MHz, CDCl_3_) δ 5.14 (ddd, *J* = 7.7, 6.1, 1.2 Hz, 1H), 3.50 (dd, *J* = 11.5, 8.1 Hz, 1H), 3.01 (dd, *J* = 11.5, 1.2 Hz, 1H), 2.76 (s, 3H), 2.41–2.31 (m, 1H), 1.05 (d, *J* = 6.8 Hz, 3H), 0.96 (d, *J* = 7.0 Hz, 3H). ^13^C NMR (101 MHz, CDCl_3_) δ 203.3, 170.8, 71.4, 30.9, 30.5, 27.0, 19.2, 17.8. The auxiliaries **5a** and **5c-e** were produced by the same method described above for **5b**.

#### 3.1.6. (R)-1-(4-Methyl-2-thioxothiazolidin-3-yl) Ethan-1-one (**5a**)

*R*_f_ = 0.22 (hexanes/EtOAc 7:3 KMnO_4_ stain); ^1^H NMR (400 MHz, CDCl_3_) δ 5.34 (tt, *J* = 6.4, 0.9 Hz, 1H), 3.64 (dd, *J* = 11.2, 7.3 Hz, 1H), 2.79 (dd, *J* = 11.2, 0.9 Hz, 1H), 2.76 (s, 3H) 1.51 (d, *J* = 6.4 Hz, 3H). ^13^C NMR (101 MHz, CDCl_3_) δ 201.3, 170.7, 63.2, 35.4, 27.1, 18.1. Exact mass for C_6_H_9_NOS_2_ [M]Na^+^: 198.0018.

#### 3.1.7. (R)-1-(4-Isobutyl-2-thioxothiazolidin-3-yl) Ethan-1-one (**5c**)

*R*_f_ = 0.46 (hexanes/EtOAc 7:3 KMnO_4_ stain); ^1^H NMR (400 MHz, CDCl_3_) δ 5.29 (dddd, *J* = 10.8, 7.3, 3.6, 0.8 Hz, 1H), 3.56 (ddd, *J* = 11.2, 7.2, 1.1 Hz, 1H), 3.07–2.86 (m, 1H), 2.76 (s, 3H), 1.91 (ddd, *J* = 13.1, 10.2, 4.2 Hz, 1H), 1.72–1.61 (m, 1H), 1.56 (dddd, *J* = 13.3, 9.7, 3.6, 1.1 Hz, 1H), 1.01 (dd, *J* = 6.4, 3.2 Hz, 6H). ^13^C NMR (101 MHz, CDCl_3_) δ 201.91, 170.54, 65.75, 39.61, 33.01, 27.06, 25.45, 23.56, 21.35. Exact mass for C_9_H_15_NOS_2_ [M]Na^+^: 240.0487.

#### 3.1.8. (R)-1-(4-Benzyl-2-thioxothiazolidin-3-yl) Ethan-1-one (**5d**)

*R*_f_ = 0.44 (hexanes/EtOAc 3:1 KMnO_4_ stain); ^1^H NMR (400 MHz, CDCl_3_) δ 7.32 (ddd, *J* = 20.2, 7.3, 1.5 Hz, 5H), 5.46–5.25 (m, 1H), 3.40 (ddd, *J* = 11.6, 7.3, 1.1 Hz, 1H), 3.23 (dd, *J* = 13.2, 3.9 Hz, 1H), 3.05 (dd, *J* = 13.2, 10.5 Hz, 1H), 2.90 (dd, *J* = 11.5, 0.7 Hz, 1H), 2.81 (s, 3H). ^13^C NMR (101 MHz, CDCl_3_) δ 201.6, 170.7, 136.5, 129.5 (2C), 128.9 (2C), 127.2, 68.2, 36.7, 31.8, 27.1. Exact mass for C_12_H_13_NOS_2_ [M]Na^+^: 274.0331.

#### 3.1.9. (R)-1-(4-Phenyl-2-thioxothiazolidin-3-yl) Ethan-1-one (**5e**)

*R*_f_ = 0.20 (hexanes/EtOAc 7:3 KMnO_4_ stain); ^1^H NMR (400 MHz, CDCl_3_) δ 7.45–7.33 (m, 5H), 6.26 (dd, *J* = 8.2, 1.3 Hz, 1H), 3.95 (dd, *J* = 11.2, 8.2 Hz, 1H), 3.09 (dd, *J* = 11.2, 1.5 Hz, 1H), 2.82 (s, 3H). ^13^C NMR (101 MHz, CDCl_3_) δ 202.6, 170.6, 139.1, 129.0 (2C), 128.5, 125.4 (2C), 69.4, 36.6, 27.2. Exact mass for C_11_H_11_NOS_2_ [M]Na^+^: 260.0174.

#### 3.1.10. (R,5Z,8Z,11Z,14Z,17Z)-3-Hydroxy-1-((R)-4-isopropyl-2-thioxothiazolidin-3-yl) Icosa-5,8,11,14,17-pentaen-1-one (**9b**)

The aldol product **9b** was prepared according to a literature procedure by Tungen and co-workers [31]. (*R*)-4-isopropyl-1,3-thiazolidine-2-thione (**5b**) (1.213 g, 5.97 mmol mmol, 2 eq.) was dissolved in CH_2_Cl_2_ (60 mL) and 1 M TiCl_4_ in CH_2_Cl_2_ (6.56 mL, 6.56 mmol mmol, 2.2 eq.) was added at −78 °C. After 5 min, DIPEA (1.25 mL, 7.16 mmol, 2.4 eq.) was added. The reaction mixture was stirred at −78 °C for 1 h. Aldehyde **4** (0.771 g, 2.98 mmol, 1 eq.) in CH_2_Cl_2_ (16 mL) was added dropwise over 20 min, and the reaction mixture was stirred for 4 h at −78 °C. The reaction was quenched by adding aqueous saturated NH_4_Cl (60 mL) and the mixture was allowed to reach ambient temperature. The aqueous phase was extracted using CH_2_Cl_2_ (3 × 60 mL). The combined organic layers were dried (Na_2_SO_4_) and the solvent removed in vacuo. The crude product was purified by column chromatography on silica (hexane/EtOAc 9:1) yielding aldol product **9b** (630 mg, 46%) as a yellow oil. In addition, its diastereomer (85 mg, 6%) was also obtained. *R*_f_ = 0.25 (hexanes/EtOAc 7:3 KMnO_4_ stain); ${\left[\alpha \right]}_{D}^{20}$ = −214.3° (CHCl_3_, c 1.08); ^1^H NMR (400 MHz, CDCl_3_) δ 5.53–5.24 (m, 10H), 5.21–5.10 (m, 1H), 4.18 (ddt, *J* = 9.0, 6.5, 3.2 Hz, 1H), 3.64 (dd, *J* = 17.7, 2.6 Hz, 1H), 3.51 Hz (dd, *J* = 11.5 Hz, 7.9 Hz, 1H), 3.16 (dd, *J* = 17.7, 9.4 Hz, 1H), 3.02 (dd, *J* = 11.5 Hz, 1.0 Hz, 1H), 2.87–2.77 (m, 9H), 2.40–2.25 (m, 3H), 2.14–1.99 (m, 2H), 1.05 (d, *J* = 6.7 Hz, 3H), 0.96 (t, *J* = 7.5 Hz, 6H). ^13^C NMR (101 MHz, CDCl_3_) δ 203.0, 173.0, 132.1, 131.2, 128.6, 128.5, 128.4, 128.1, 128.0 (2C), 127.1, 125.1, 71.4, 67.9, 45.0, 34.2, 30.9, 30.7, 25.9, 25.8, 25.7, 25.6, 20.7, 19.2, 17.9, 14.4. Exact mass for C_26_H_39_NO_2_S_2_ [M]Na^+^: 484.2314.

#### 3.1.11. **9b** Diastereomer (Minor Product)

*R*_f_ = 0.37 (hexanes/EtOAc 7:3 KMnO_4_ stain); ${\left[\alpha \right]}_{D}^{20}$ = −197.0° (CHCl_3_, c 0.46); ^1^H NMR (400 MHz, CDCl_3_) δ 5.66–5.25 (m, 10H), 5.17 (ddd, *J* = 7.8, 6.3, 1.2 Hz, 1H), 4.11–4.01 (m, 1H), 3.57–3.34 (m, 3H), 3.24 (bs, 1H), 3.03 (dd, *J* = 11.5, 1.2 Hz, 1H), 2.95–2.69 (m, 8H), 2.45–2.26 (m, 3H), 2.06 (tdd, *J* = 7.6, 7.0, 1.5 Hz, 2H), 1.05 (d, *J* = 6.8 Hz, 3H), 0.96 (t, *J* = 7.5 Hz, 6H). ^13^C NMR (101 MHz, CDCl_3_) δ 203.1, 173.6, 132.1, 131.1, 128.7, 128.5, 128.4, 128.1, 128.0 (2C), 127.1, 125.1, 71.4, 68.3, 44.7, 34.4, 30.9, 30.7, 25.9, 25.8, 25.7 (2C), 20.7, 19.2, 17.9, 14.4. Exact mass for C_26_H_39_NO_2_S_2_ [M]Na^+^: 484.2314.

#### 3.1.12. (S)-1-((R,5Z,8Z,11Z,14Z,17Z)-3-((Tert-butyldimethylsilyl)oxy)icosa-5,8,11,14,17-pentaenoyl)-5-isopropylpyrrolidin-2-one (**10**)

The aldol product **9b** (0.085 g, 0.18 mmol) was dissolved in CH_2_Cl_2_ (9.5 mL). The solution was cooled on a dry ice/ethanol bath. After 10 min, 2,6-lutidine (0.064 mL, 0.059 g, 0.55 mmol) was added. The reaction mixture was stirred for an additional 10 min, and TBSOTf (0.063 mL, 0.073 g, 0.28 mmol) was added dropwise. The reaction was stirred at this temperature for two hours before it was quenched with a saturated aqueous solution of NH_4_Cl (5 mL). The phases were separated. The water phase was extracted with CH_2_Cl_2_ (3 × 10 mL). The combined organic phases were dried (Na_2_SO_4_), filtered and concentrated in vacuo. The crude oil was purified by column chromatography on silica (EtOAc/hexane 5:95) to give compound **10** (0.077 g, 0.13 mmol, 72%) as a yellow oil. Rf: 0.70 (EtOAc:Heksan 3:7 KMnO_4_ stain). [α]: −126.5° (CHCl_3_, c 0.68) IR (film): 3014, 2964, 1700 cm^−1^. UV: λ_max_ 262, 310 nm. ^1^H NMR (400 MHz, CDCl_3_): δ 5.53–5.27 (m, 11H), 5.03 (ddd, *J* = 7.6, 6.3, 1.0 Hz, 1H), 4.39–4.31 (m, 1H), 3.58–3.42 (m, 2H), 3.14 (dd, *J* = 17.1, 3.7 Hz, 1H), 3.02 (dd, *J* = 11.4, 1.0 Hz, 1H), 2.88–2.77 (m, 8H), 2.42–2.26 (m, 3H), 2.14–2.02 (m, 2H), 1.06 (d, *J* = 6.7 Hz, 3H), 0.99–0.94 (m, 6H), 0.89–0.84 (m, 8H), 0.09 (s, 3H), 0.04 (s, 3H). ^13^C NMR (100 MHz, CDCl_3_): δ 202.8, 172.0, 132.1, 130.6, 128.7, 128.4, 128.2, 128.0, 127.2, 125.4, 71.7, 69.2, 45.3, 35.7, 31.1, 30.9, 26.0, 25.8, 25.8, 25.7, 20.7, 19.3, 18.1, 18.0, 14.4, −4.3, −4.6. 

#### 3.1.13. Ethyl (R,5Z,8Z,11Z,14Z,17Z)-3-((Tert-butyldimethylsilyl)oxy)icosa-5,8,11,14,17-penta-enoate (**11**)

Silyl ether **10** (0.041 g, 0.072 mmol) was dissolved in absolute EtOH (1.45 mL) and cooled to 0 °C. K_2_CO_3_ (0.015 g, 0.11 mmol) was added to the solution, and the reaction mixture was left stirring for 3 h, then slowly allowed to reach room temperature, and stirred for an additional 23 h. The reaction was stopped by adding saturated aqueous NH_4_Cl (10 mL). The product was extracted with CH_2_Cl_2_ (3 × 10 mL). The combined organic phase was washed with 1 M KOH (5 mL), saturated aqueous NaCl (10 mL) and dried over Na_2_SO_4_. Filtration and concentration in vacuo gave a crude oil, which was purified by column chromatography on silica (EtOAc/hexane 1:39) to give ethyl ester **11** (0.026 g, 0.056 mmol, 78%) as a colorless oil. Rf: 0.53 (EtOAc:hexane 1:9). [α]: −22.6° (CHCl3, c 0.39). IR (film): 3014, 2931, 2858, 1739 cm^−1^. UV: λ_max_ 246 nm. ^1^H NMR (400 MHz, CDCl_3_): δ 5.57–5.18 (m, 10H), 4.28–4.01 (m, 3H), 2.89–2.73 (m, 8H), 2.42 (dd, *J* = 6.3, 2.5 Hz, 2H), 2.35–2.23 (m, 2H), 2.16–2.00 (m, 2H), 1.25 (t, *J* = 7.2 Hz, 3H), 0.97 (t, *J* = 7.5 Hz, 3H), 0.86 (s, 9H), 0.07 (s, 3H), 0.04 (s, 3H). ^13^C NMR (100 MHz, CDCl_3_): δ 171.96, 132.18, 130.43, 128.72, 128.44, 128.42, 128.20, 128.16, 128.01, 127.16, 125.41, 69.47, 60.46, 42.47, 35.62, 25.95, 25.90, 25.80, 25.77, 25.69, 20.71, 18.13, 14.43, 14.35, −4.31, −4.79. 

#### 3.1.14. Synthesis of 3R-HEPE (**2**)

At 0 °C, TBAF in THF (1 M, 0.26 mL, 0.26 mmol) was added to a stirred solution of ethyl ester **11** (0.024 g, 0.052 mmol) in THF (1 mL). The reaction was stirred at this temperature for seven hours before it was quenched with phosphate buffer (pH = 7.29, 1 mL). Saturated aqueous NaCl (5 mL) and EtOAc (5 mL) was added, followed by the separation of the phases. The aqueous phase was extracted with EtOAc (2 × 5 mL). The combined organic phases were dried (Na_2_SO_4_), filtered and concentrated in vacuo. The crude oil was purified by column chromatography on silica (EtOAc/hexane 3:7) to give the ethyl ester of 3*R*-HEPE (**2**) (0.010 g, 0.030 mmol, 58%) as a colorless oil. R_f_: 0.49 (EtOAc:Heksan 3:7) [α]: −19.4° (CHCl_3_, c 1.03). IR (film): 3507, 3014, 2964, 1734 cm^−1^. UV: λ_max_ 245 nm. ^1^H NMR (400 MHz, CDCl_3_): δ 5.70–5.15 (m, 10H), 4.17 (q, *J* = 7.1 Hz, 2H), 4.12–4.01 (m, 1H), 2.94 (d, *J* = 2.8 Hz, 1H), 2.90–2.68 (m, 8H), 2.52 (dd, *J* = 16.4, 3.4 Hz, 1H), 2.42 (dd, *J* = 16.4, 8.9 Hz, 1H), 2.38–2.21 (m, 2H), 2.07 (pd, *J* = 7.5, 1.3 Hz, 2H), 1.27 (t, *J* = 7.2 Hz, 3H), 0.97 (t, *J* = 7.5 Hz, 3H). ^13^C NMR (100 MHz, CDCl_3_): δ 172.98, 132.19, 131.21, 128.74, 128.56, 128.47, 128.14, 127.99, 127.96, 127.15, 125.04, 68.00, 60.86, 40.78, 34.48, 25.93, 25.80, 25.78, 25.69, 20.70, 14.41, 14.32. The obtained ethyl ester (0.036 g, 0.10 mmol) was dissolved in THF/EtOH/H2O 2:2:1 (11 mL), followed by the addition of LiOH·H_2_O (0.153 g, 3.64 mmol) 0 °C. The reaction mixture was allowed to reach room temperature and stirred for 1.5 h. The solvent was evaporated in vacuo. EtOAc (10 mL) and NaH_2_PO_4_ (5 mL) were added. The phases were separated. The water phase was extracted with EtOAc (2 × 10 mL). The combined organic phases were dried over Na_2_SO_4_, filtered and concentrated in vacuo. This gave acid 3*R*-HEPE (**2**) (0.030 g, 0.095 mmol, 95%) as a colorless oil. R_f_: 0.81 (MeOH:CH_2_Cl_2_ 1:3). [α]: −10.5° (CHCl_3_, c 0.86). IR (film): 3014, 2964, 2931, 1712 cm^−1^. UV: λ_max_ 253 nm (ε 561). ^1^H NMR (400 MHz, CDCl_3_): δ 5.62–5.51 (m, 1H), 5.50–5.26 (m, 9H), 4.16–4.02 (m, 1H), 2.93–2.73 (m, 8H), 2.59 (dd, *J* = 16.5, 3.4 Hz, 1H), 2.49 (dd, *J* = 16.6, 8.9 Hz, 1H), 2.42–2.24 (m, 2H), 2.07 (pd, *J* = 7.3, 1.3 Hz, 2H), 0.97 (t, *J* = 7.5 Hz, 3H). ^13^C NMR (100 MHz, CDCl_3_): δ 177.69, 132.19, 131.63, 128.74, 128.65, 128.50, 128.09, 127.97, 127.82, 127.14, 124.65, 67.89, 40.61, 34.46, 25.93, 25.79, 25.77, 25.68, 20.69, 14.40. 

#### 3.1.15. 3R-HEPE (**2**) from Aldol Product **9b**

The aldol product **9b** (100 mg, 0.216 mmol, 1 eq.) was dissolved in a solution of THF/MeOH/H_2_O (2:2:1) (30 mL) and the solution was cooled to 0 °C. LiOH·H_2_O (0.318 g, 7.56 mmol, 35 eq.) was added and the reaction mixture was stirred for 2 h, allowed to reach ambient temperature during this time. The solvent was removed in vacuo and EtOAc (30 mL) was added. The solution was acidified with aqueous saturated NaH_2_PO_4_ (20 mL) and the aqueous layer was extracted with EtOAc (2 × 30 mL). The combined organic layers were dried (Na_2_SO_4_) and the solvent was removed in vacuo. The crude product was purified by column chromatography on silica (CH_2_Cl_2_/MeOH/HCO_2_H 95:5:0.0125) and **2** (60.0 mg) was obtained as a light-yellow oil in 87% yield. *R*_f_ = 0.20 (DCM/MeOH 95:5 KMnO_4_ stain); ${\left[\alpha \right]}_{D}^{20}$ = −10.5° (CHCl_3_, c 1.08). The spectral data were similar as those obtained above. 

#### 3.1.16. (R)-2-Hydroxy-1,2,2-triphenylethyl (S,5Z,8Z,11Z,14Z,17Z)-3-hydroxyicosa-5,8,11,14,17-pentaenoate (**13**)

The aldol product **13** was prepared according to a literature procedure by Braun and co-workers [27]. Auxiliary **12** (81.5 mg, 0.245 mmol, 1 eq.) was added to dry THF (1.28 mL) under argon atmosphere. The slurry was cooled to −78 °C and the 2.5 M LDA solution (0.27 mL, 0.27 mmol, 1.1 eq.) was added dropwise over 10 min. The mixture was slowly warmed to −10 °C over 2 h and stirred further for 40 min at this temperature. The solution was cooled to −78 °C and **4** (85 mg, 0.33 mmol, 1.35 eq.) in THF (0.25 mL) was added dropwise over 30 min. The mixture was stirred for 2 h at −78 °C. The reaction was quenched by a dropwise addition of saturated ammonium chloride solution (1.5 mL). The solution was warmed to −5 °C and diluted with water (1.5 mL). The mixture was extracted with EtOAc (2 × 3 mL). The combined organic layers were dried (Na_2_SO_4_), filtered and concentrated in vacuo. The crude oil was purified by column chromatography on silica gel (EtOAc/Et_2_O/Toluene/hexane 10:15:10:65), affording the aldol product **13** (81.3 mg) in a 70% yield. *R*_f_ = 0.25 (hexane/EtOAc 8:2 KMnO_4_ stain); ^1^H NMR (400 MHz, CDCl_3_) δ 7.63–7.54 (m, 2H), 7.38 (dd, *J* = 8.4, 6.8 Hz, 2H), 7.33–7.28 (m, 1H), 7.22–7.11 (m, 8H), 7.10–7.01 (m, 2H), 6.74 (s, 1H), 5.66–5.45 (m, 1H), 5.36 (m, 9H), 3.96–3.85 (m, 1H), 2.75–2.85 (m, 8H), 2.54–2.31 (m, 2H), 2.25–2.13 (m, 2H), 2.13–2.04 (m, 2H), 0.98 (t, *J* = 7.5 Hz, 3H). ^13^C NMR (101 MHz, CDCl_3_) δ 171.3, 144.5, 142.5, 135.4, 132.1, 131.1, 128.6, 128.4 (3C), 128.1, 128.0, 127.9 (2C), 127.8, 127.6, 127.5, 127.2, 127.0, 126.2 (2C), 124.7, 80.3, 79.0, 67.8, 41.1, 34.2, 25.8, 25.7, 25.6, 20.6, 14.3. Exact mass for C_40_H_46_O_4_ [M]Na^+^: 613.3288.

#### 3.1.17. Methyl (S,5Z,8Z,11Z,14Z,17Z)-3-Hydroxyicosa-5,8,11,14,17-pentaenoate (S-**14**)

To a solution of **13** (16.3 mg, 0.027 mmol, 1 eq.) in MeOH (0.5 mL) was added K_2_HPO_4_ (0.6 mg, 0.0027 mmol, 0.1 eq.). The mixture was heated at reflux for 20 h and then concentrated in vacuo. The crude product was purified by column chromatography on silica gel (EtOAc/hexanes 0:100 → 20:80), affording **14** (7.3 mg) in a 80% yield. *R*_f_ = 0.28 (hexanes/EtOAc 8:2 KMnO_4_ stain); ${\left[\alpha \right]}_{D}^{20}$ = +9.3° (CHCl_3_, c 1.08); ^1^H NMR (400 MHz, CDCl_3_) δ 5.60–5.50 (m, 1H), 5.49–5.29 (m, 9H), 4.20–3.94 (m, 1H), 3.72 (s, 3H), 2.84 (m, 8H), 2.55 (dd, *J* = 16.4, 3.4 Hz, 1H), 2.50–2.42 (m, 1H), 2.40–2.22 (m, 2H), 2.20–1.90 (m, 2H), 0.99 (t, *J* = 7.5 Hz, 3H). ^13^C NMR (101 MHz, CDCl_3_) δ 173.0, 132.2, 131.2, 128.7, 128.6, 128.5, 128.1, 128.0 (2C), 127.2, 125.0, 68.0, 60.9, 40.8, 34.5, 25.9, 25.8 (2C), 25.7, 20.7, 14.4. Exact mass for C_21_H_32_NaO_3_ [M]Na^+^: 355.2244.

#### 3.1.18. 3S-HEPE (**2**)

Methyl ester **S-14** (11.1 mg, 0.033 mmol, 1 eq.) was dissolved in a solution of THF/MeOH/H_2_O (2:2:1) (5 mL) and the solution was cooled to 0 °C. LiOH·H_2_O (49.1 mg, 1.17 mmol, 35 eq.) was added and the reaction mixture was stirred for 2 h, allowed to reach ambient temperature during this time. The solvent was removed in vacuo and EtOAc (5 mL) was added. The solution was acidified with aqueous saturated NaH_2_PO_4_ (5 mL) and the aqueous layer was extracted with EtOAc (2 × 5 mL). The combined organic layers were dried (Na_2_SO_4_) and the solvent was removed in vacuo. The crude product was purified by column chromatography on silica (CH_2_Cl_2_/MeOH/HCO_2_H 95:5:0.0125) and (*S*)-**2** (10.0 mg) was obtained as a light-yellow oil in a 94% yield. *R*_f_ = 0.20 (DCM/MeOH 95:5 KMnO_4_ stain); ${\left[\alpha \right]}_{D}^{20}$ = +11.1 (CHCl_3_, c 0.90); ^1^H NMR (400 MHz, CDCl_3_) δ 5.62–5.51 (m, 1H), 5.50–5.26 (m, 9H), 4.16–4.02 (m, 1H), 2.93–2.73 (m, 8H), 2.59 (dd, *J* = 16.5, 3.4 Hz, 1H), 2.49 (dd, *J* = 16.6, 8.9 Hz, 1H), 2.42–2.24 (m, 2H), 2.07 (dd, *J* = 7.3 Hz, 1.3 Hz, 2H), 0.97 (t, *J* = 7.5 Hz, 3H). ^13^C NMR (101 MHz, CDCl_3_) δ 177.69, 132.19, 131.63, 128.74, 128.65, 128.50, 128.09, 127.97, 127.82, 127.14, 124.65, 67.89, 40.61, 34.46, 25.93, 25.79, 25.77, 25.68, 20.69, 14.40. Exact mass for C_20_H_30_NaO_3_ [M]Na^+^: 341.2087

#### 3.1.19. Methyl (S,5Z,8Z,11Z,14Z,17Z)-3-Hydroxyicosa-5,8,11,14,17-pentaenoate (R-**14**)

For the comparison of both enantiomers in Mosher analysis, (*R*)-14 was prepared as follows [38]. Trimethylsilyl diazomethane (2.0 M in hexanes, 1 mL, 2 mmol) was added dropwise to a solution of the 3R-HEPE (2) in methanol (3 mL) at 0 °C until a yellow color persisted in the reaction. The reaction was quenched by the addition of acetic acid (2 drops) and concentrated. The crude residue was submitted to the next reaction without further purification. ^1^H NMR (300 MHz, CDCl_3_) δ (ppm): 5.88–5.75 (m, 1H), 5.18–5.11 (m, 2H), 3.97 (major, ddd, *J* = 6.9, 5.7, 3.9 Hz, 0.8H), 3.81–3.73 (minor, 0.2H), 3.72 (s, 3H), 2.58 (dddd, *J* = 6.9, 6.9, 6.9, 4.2 Hz, 1H), 2.32–3.19 (m, 2H), 1.22 (d, *J* = 6.9 Hz, 3H). 

### 3.2. Mosher Analysis

Mosher esters were prepared according to the procedure by Hoye et al. [32]. To a 4 mL glass vial with a Teflon-coated magnetic stir bar, methyl ester R-**14** and S-**14** (20 mg, 60 mmol) and dry pyridine (16 µL, 0.20 mmol) were added. CHCl_3_ (1 mL) was passed through a pipette with fresh silica gel to make sure no alcohol or water were present, then added to the vial. MTPA-Cl (23 mL, 0.12 mmol) was added to the mixture. The vial was capped and left stirring in the dark at room temperature for two hours. Ether (3 mL) and water (1 mL) were added, and the layers were mixed thoroughly. The layers were separated, and the extraction was repeated two times. The combined organic layers were dried over anhydrous solid Na_2_SO_4_ and the solvents were evaporated on rotary evaporator. ^1^H and ^19^F NMR were recorded. The ^1^H NMR results were inclusive, while changes were seen in ^19^F NMR. Data were analyzed as described by Hoye et al. [32,33]. Results are reported in Table 2. 

## 4. Conclusions

3-Hydroxy polyunsaturated oxylipins, in particular 3-hydroxyeicosapentaenoic acid (**2**), show complex roles in fungal pathogenesis [39]. The oxylipin **2** may serve as a substrate for the biosynthesis of pro-inflammatory hydroxylated PUFAs [5]. Hence, biological investigations are in demand that require access to synthetic material. In this paper, the total synthesis of both enantiomers of **2** is presented, using acetate aldol methodologies. This methodology is of current interest in the total synthesis of natural products [40]. Sufficient amounts of materials for conducting biological and biosynthetic studies are now available. Results from such efforts will be reported in due time. 

## Figures and Tables

**Table 1:** Results from the Nagao–Fujita acetate aldol reaction with aldehyde **4**.

Entry	R	Lewis Acid	Base	dr ^a^ (*R,R*:R,*S*)	Yield (%) ^b^
1	**a**: Me	TiCl_4_	DIPEA ^c^	2.6:1	22
2	**b**: *i*-Pr	TiCl_4_	DIPEA ^c^	10:1	46
3	**b**: *i*-Pr	TiCl_4_	NMP ^d^	1.2:1	56
4	**c**: *i*-Bu	TiCl_4_	DIPEA ^c^	1.9:1	25
5	**d**: Bn	TiCl_4_	DIPEA ^c^	5.3:1	30
6	**e**: Ph	TiCl_4_	DIPEA ^c^	nr ^e^	N/A
7	**b**: *i*-Pr	TiCl_4_	(−)-sparteine	5.8:1	35

^a^: Determined by ^1^H NMR analyses. ^b^: After chromatographic purification and isolation of major diastereomer. ^c:^ *N*,*N*-diisopropylethylamine. ^d^: *N*-methyl-2-pyrrolidinone. ^e^: No reaction.

**Table 2:** Combinations of configurations of 3-hydrocyeicosapentaenoic acids MTBA-esters (**15**) and their chemical shifts.

Assumed Configuration of Carbinol	MTPA Configuration	^19^F NMR Shifts	Δδ^SR^ (= δ_S_ − δ_R_)	Found Configuration of Carbinol
*R*	*S* *R*	−72.42 −72.38	−0.04	*R*
*S*	*S* *R*	−72.37 −72.65	+0.28	*S*

## Data Availability

Not applicable.

## Supplementary Materials

The following supporting information can be downloaded at: https://www.mdpi.com/article/10.3390/molecules27072295/s1, spectra of IR, UV, MS, ^1^H and ^13^C NMR as well as HPLC chromatograms.

## Sample Availability

Samples of (*R*)- and (*S*)-enantiomers of 3-hydroxyeicosapentaenoic acid (**2**) are available from the authors.
