# Supplementary material for: The Synthesis of 3-(R)- and 3-(S)-Hydroxyeicosapentaenoic Acid
Source: Molecules. 2022 Apr 1;27(7):2295. doi: 10.3390/molecules27072295 (PMC9000449; doi:10.3390/molecules27072295)
Supplement: Supplementary file 1 [file molecules-27-02295-s001.zip › molecules-1662926-supplementary.pdf]

Supplementary Material

Synthesis of 3-(*R*)- and 3-(*S*)-Hydroxyeicosapentaenoic Acid

Gard Gjessing [1], Lars-Inge Gammelsæter Johnsen [2], Simen Gjelseth Antonsen [1,3], Jens M. J. Nolsøe [1], Yngve Stenstrøm [1,*] and Trond Vidar Hansen [1,2,*]

[1] Department of Chemistry, Biotechnology and Food Science, Norwegian University of Life Sciences, P.O. Box 5003, NO-1433 Ås, Norway; gjegar@gmail.com (G.G.); simen.antonsen@oslomet.no (S.G.A.); jens.mj.nolsoe@nmbu.no (J.M.J.N.)
[2] Section of Pharmaceutical Chemistry, Department of Pharmacy, University of Oslo, P.O. Box 1068 Blindern, NO-0316 Oslo, Norway; lars.inge1988@gmail.com
[3] Department of Mechanical, Electronic and Chemical Engineering, Faculty of Technology, Art and Design, OsloMet, P.O. Box 4, St. Olavs Plass, NO-0130 Oslo, Norway
* Correspondence: yngve.stenstrom@nmbu.no (Y.S.); t.v.hansen@farmasi.uio.no (T.V.H.)

Table of Contents

Iodolactone 6: 2
Epoxide 7: 5
Acetal 8: 8
Aldehyde 4: 11
Auxiliary 5b: 14
Aldol products 9b: 17
TBS protected aldol product 10: 27
TBS protected ethyl ester 11: 32
3(R)-HEPE (2): 37
Aldol product 13: 45
(R)-4-isopropylthiazolidinone-2-thione 15: 47
Ethyl ester 16: 50
Mosher esters: 55

Iodolactone 6:

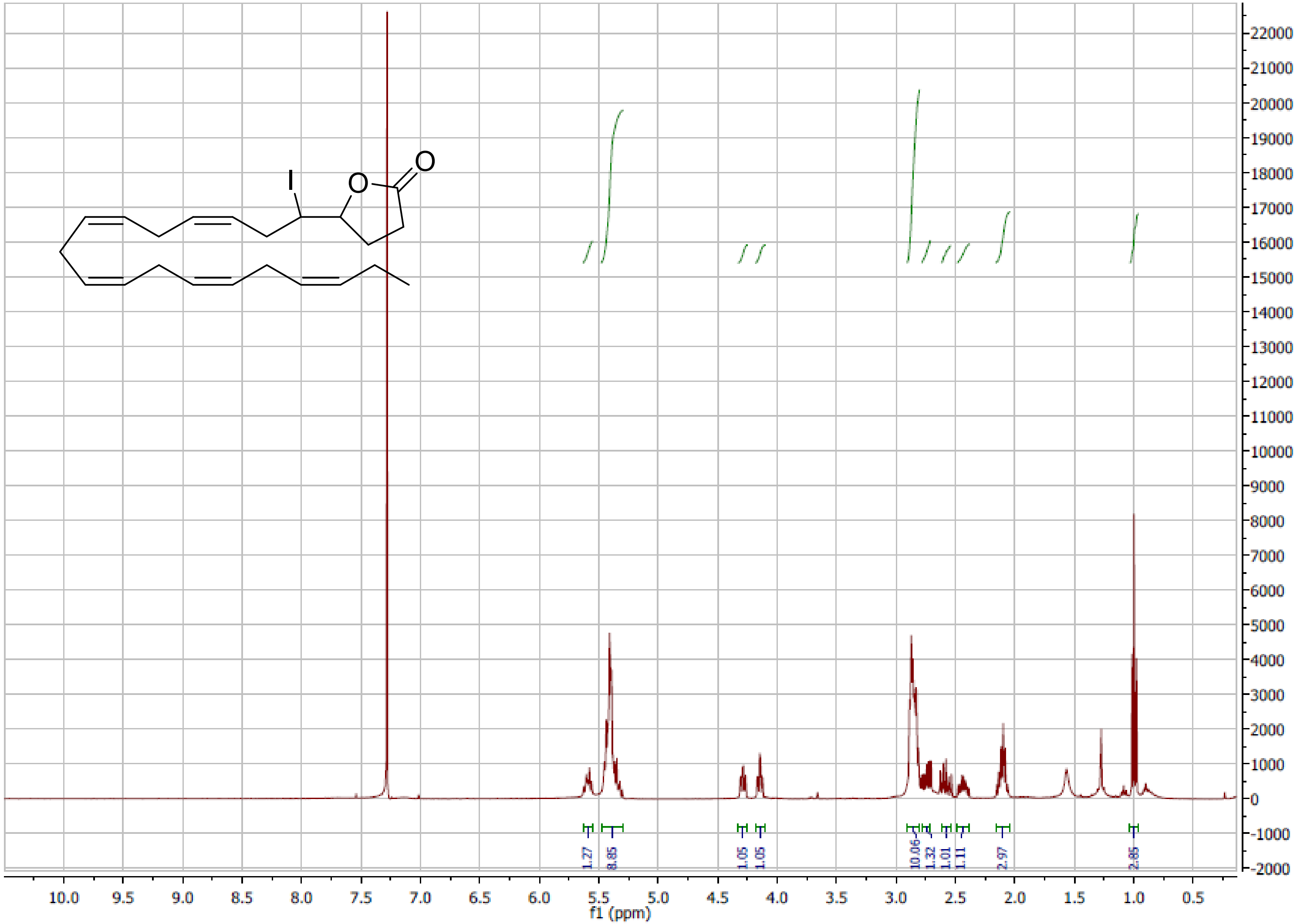

Figure S1 1H NMR spectrum of iodolactone 6.

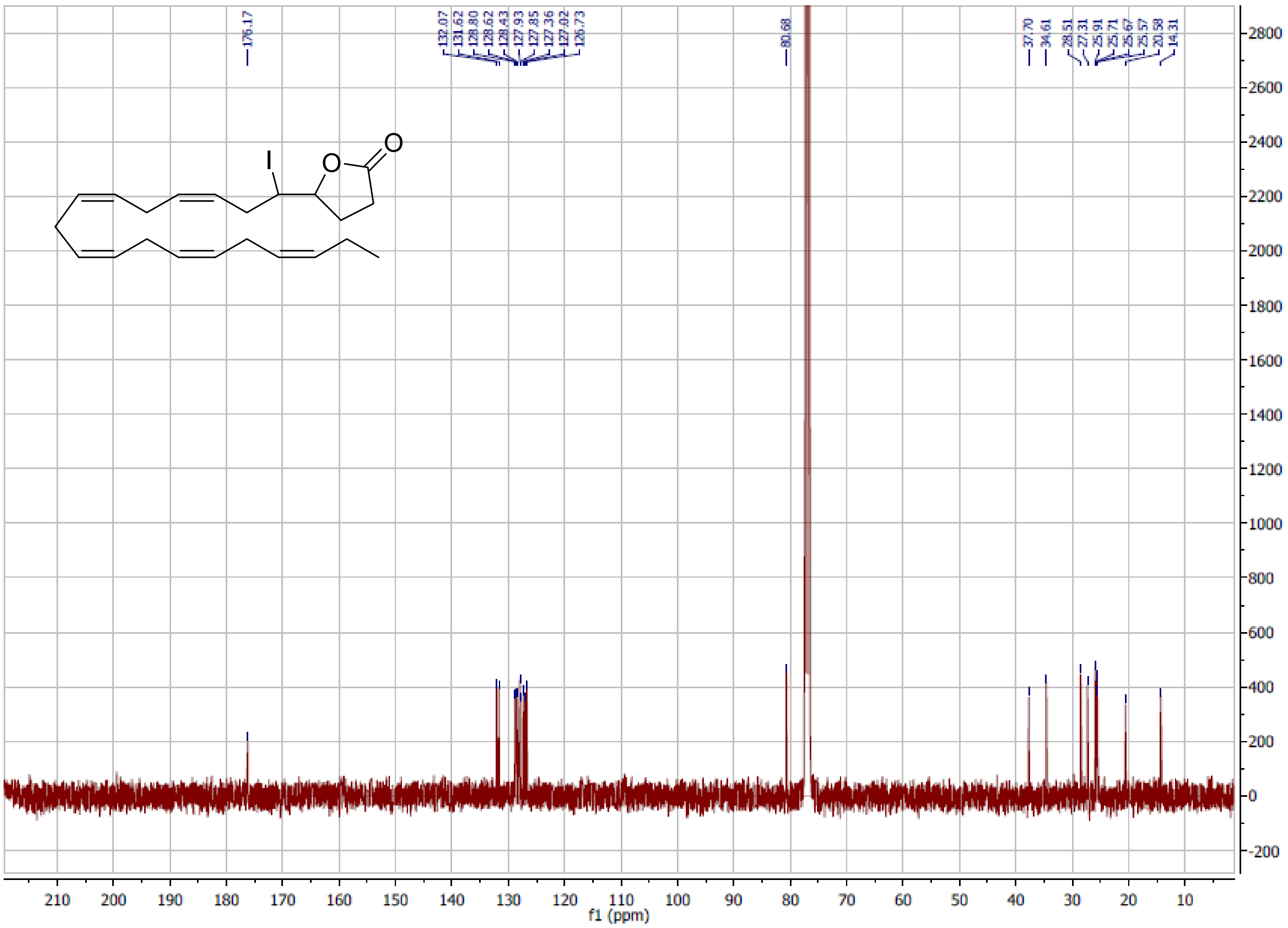

Figure S2 ^{13}C NMR spectrum of iodolactone **6**.

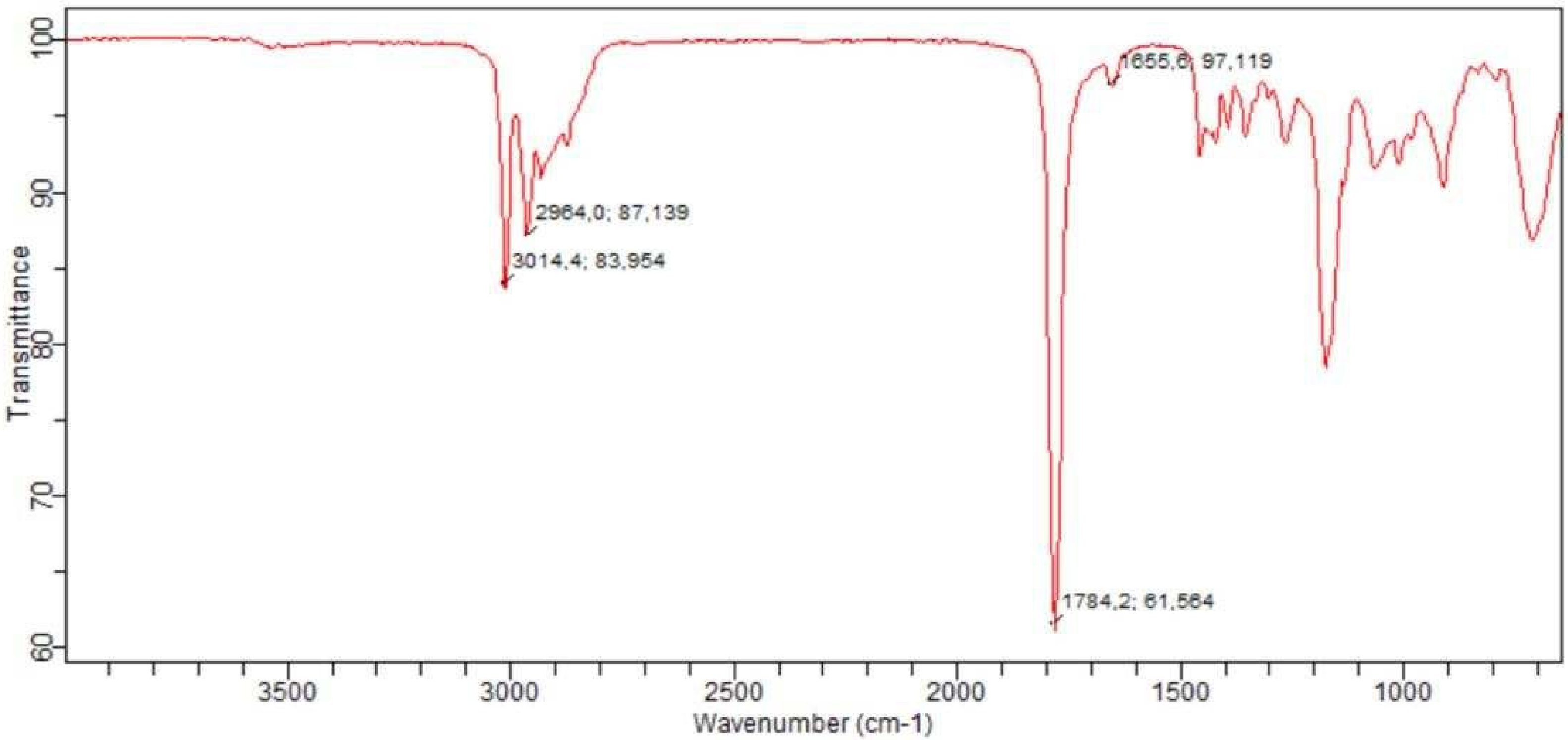

Figure S3 IR of iodolactone 6.

Epoxide 7:

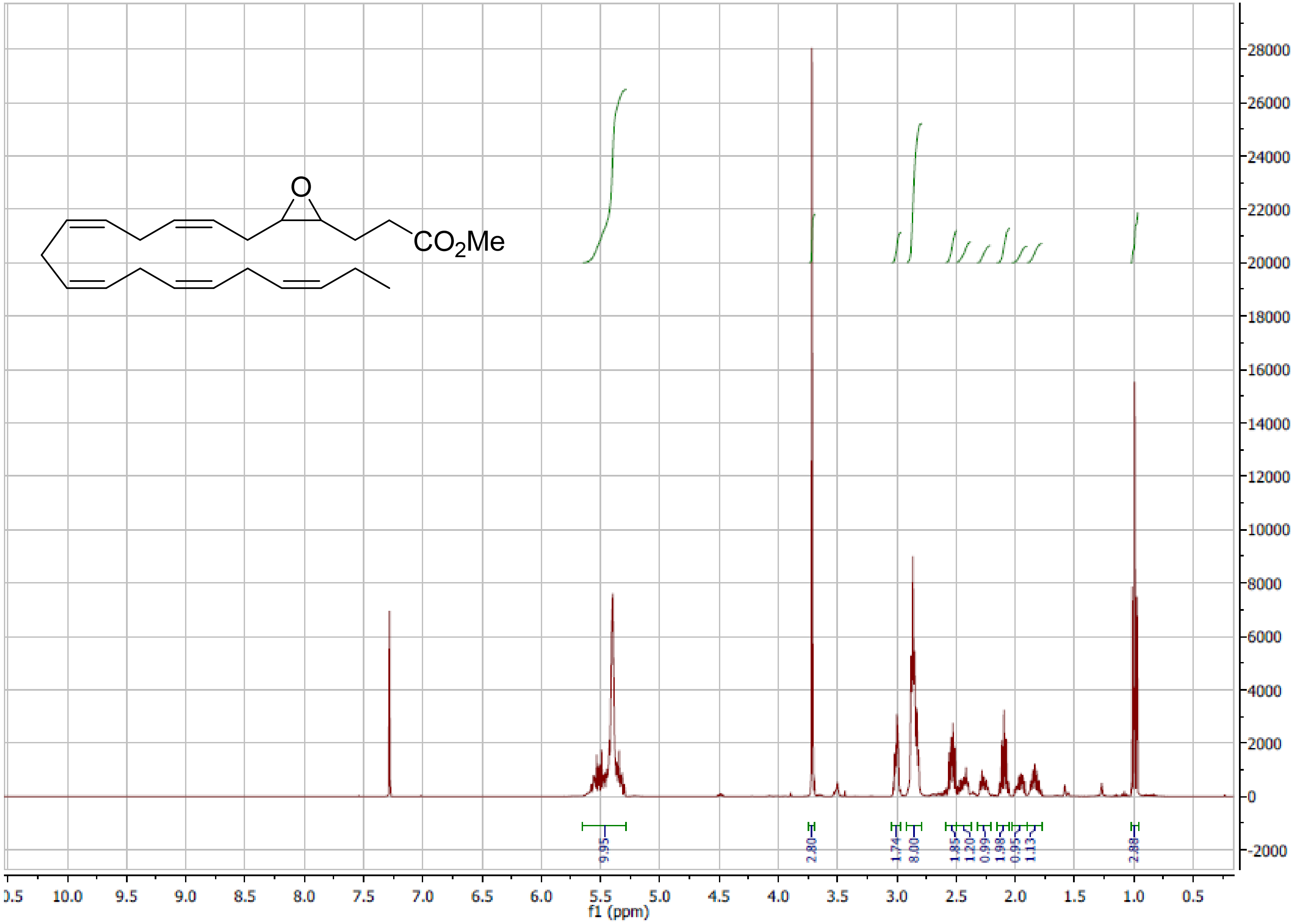

Figure S4 ^{1}H NMR spectrum of epoxide **7**.

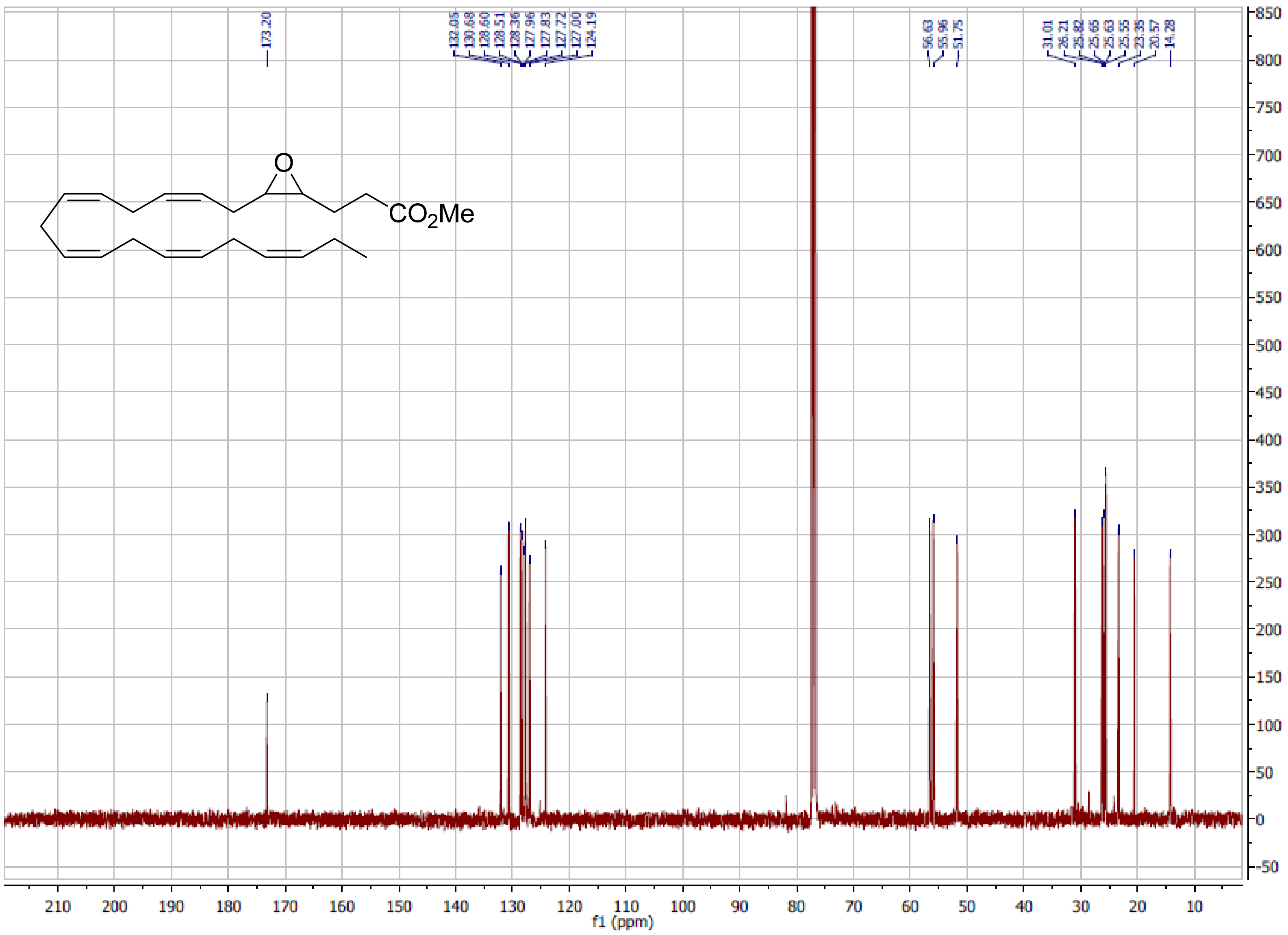

Figure S5 ^{13}C NMR spectrum of epoxide **7**.

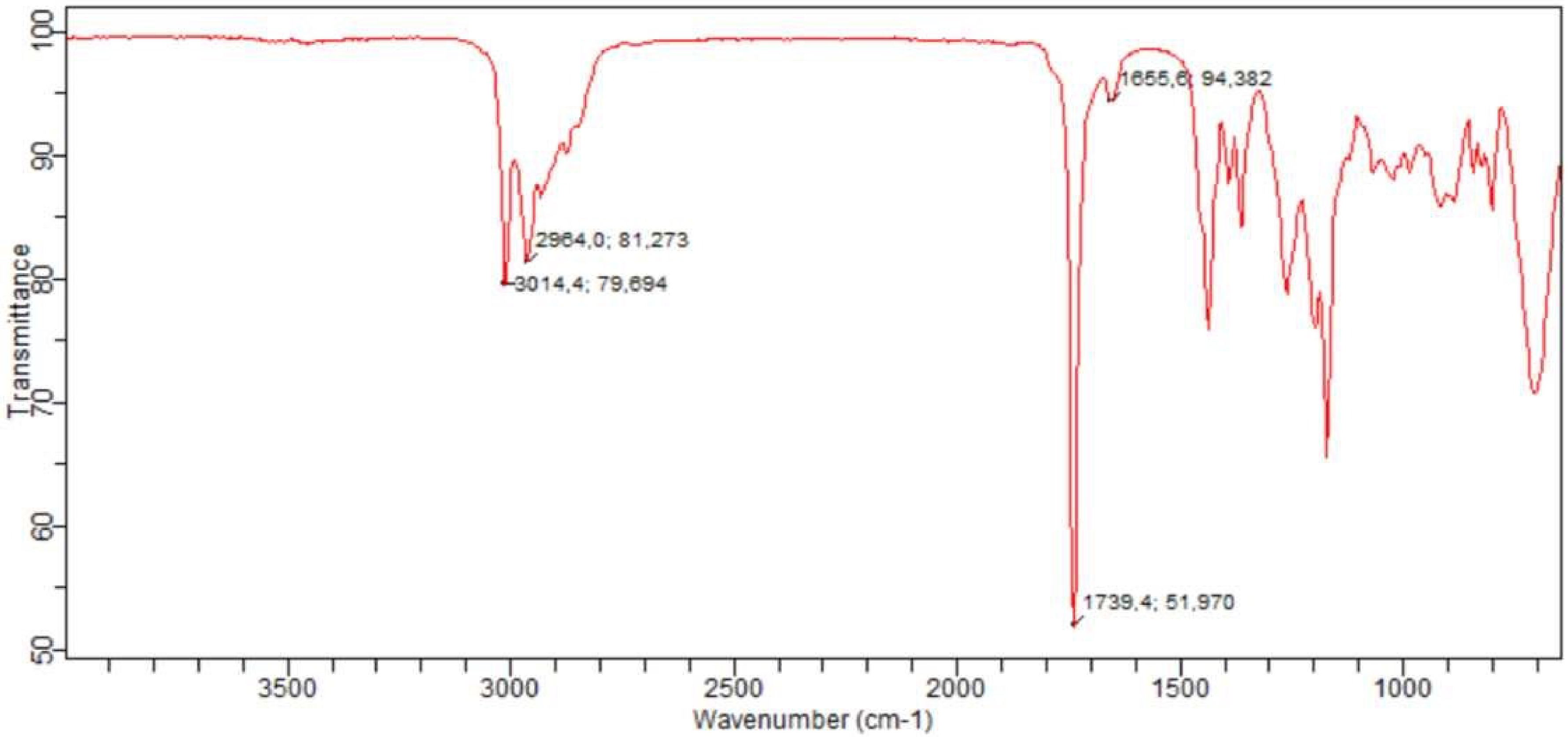

*Figure S6 IR spectrum of epoxide **7***

Acetal 8:

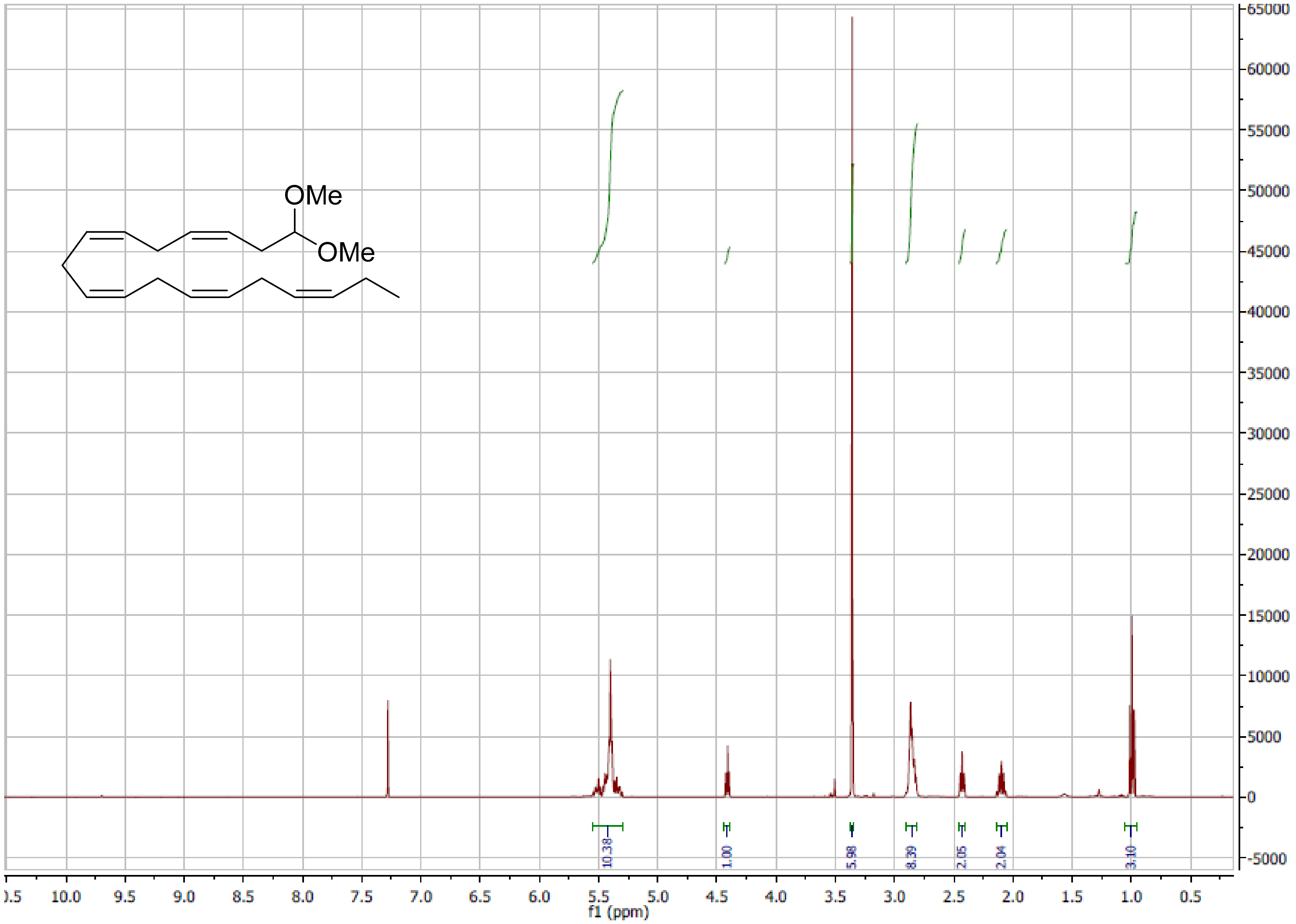

Figure S7 ^{1}H NMR spectrum of acetal **8**.

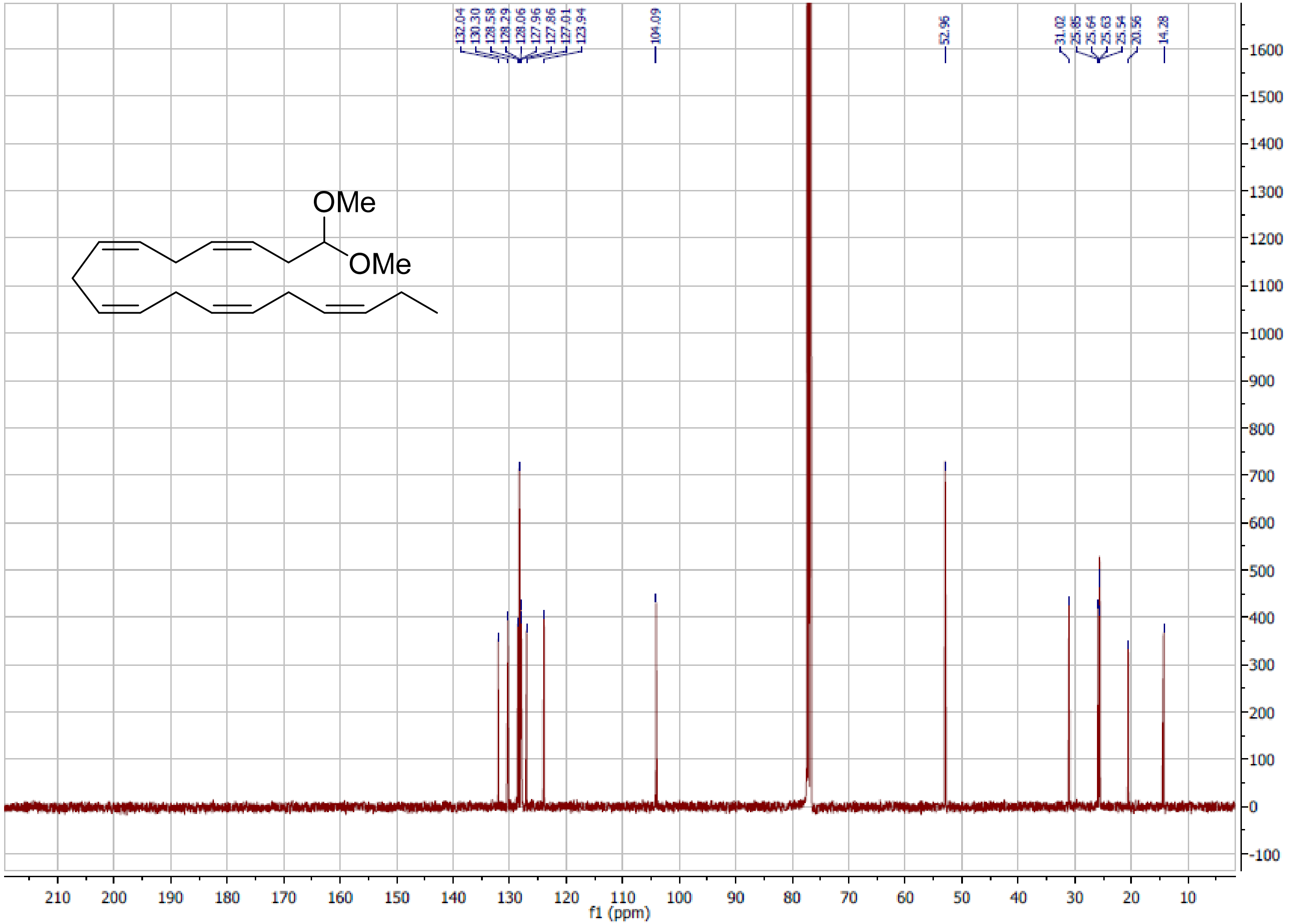

Figure S8 ^{13}C NMR spectrum of acetal **8**.

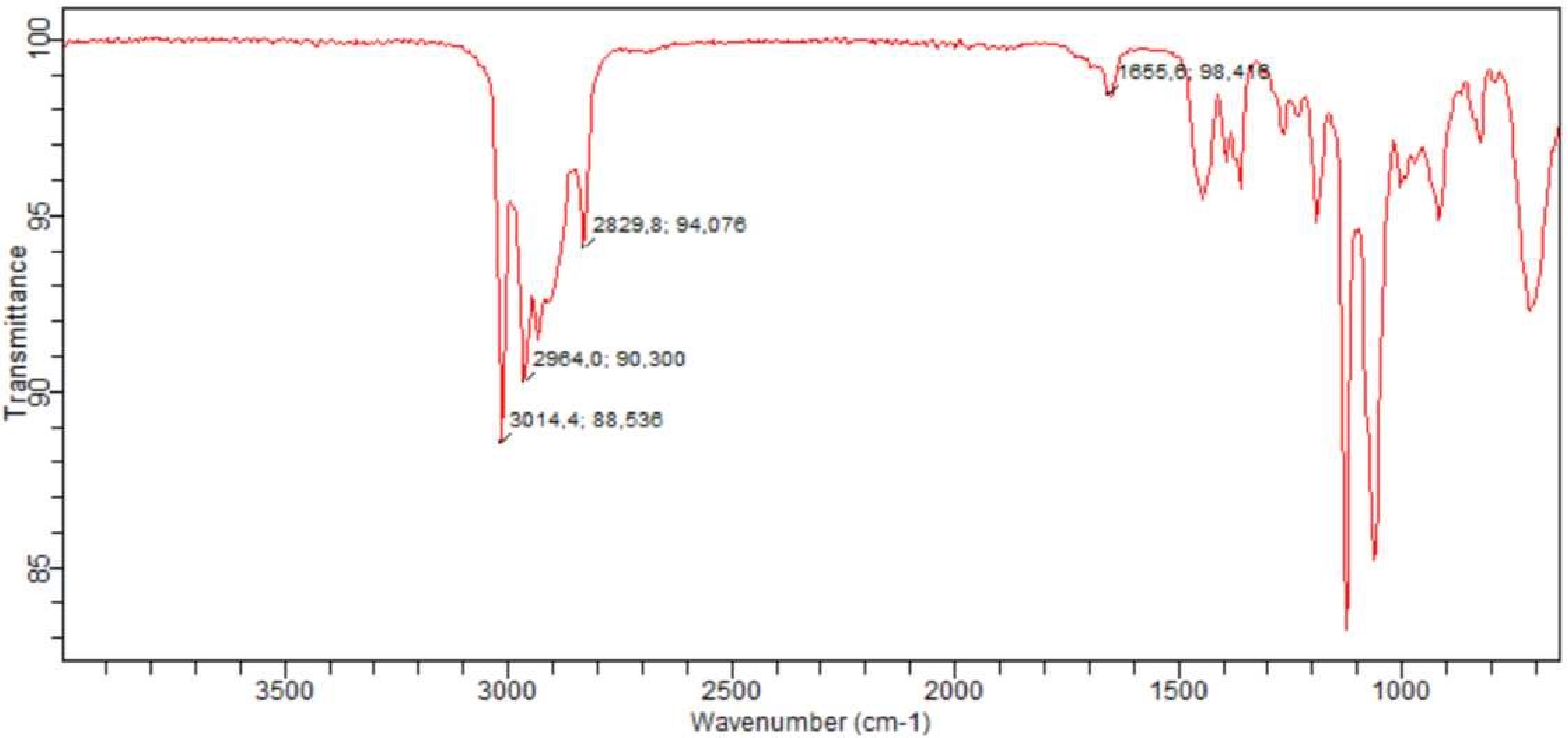

Figure S9 IR spectrum of acetal 8.

Aldehyde 4:

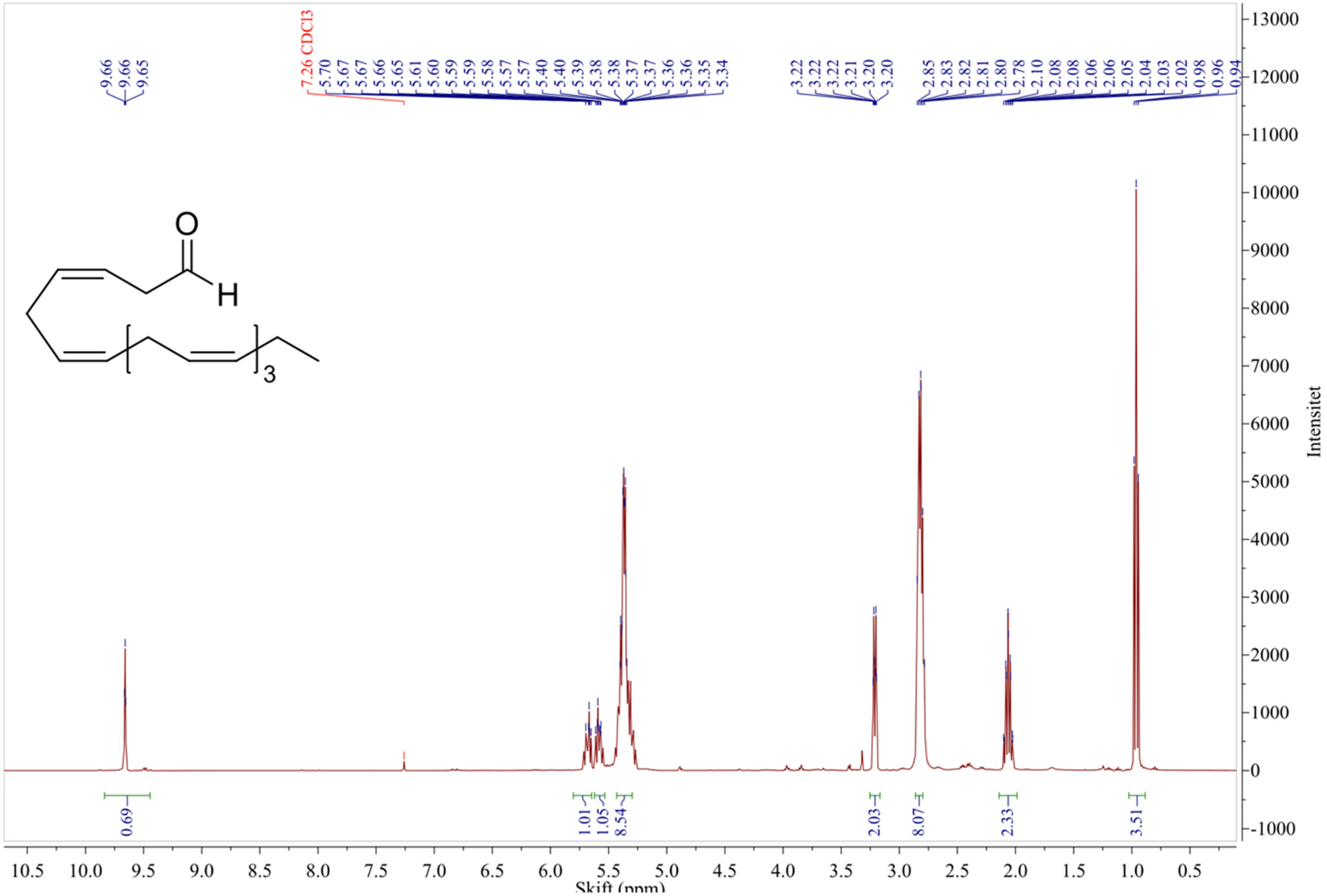

Figure S10 1H NMR spectrum of aldehyde 4

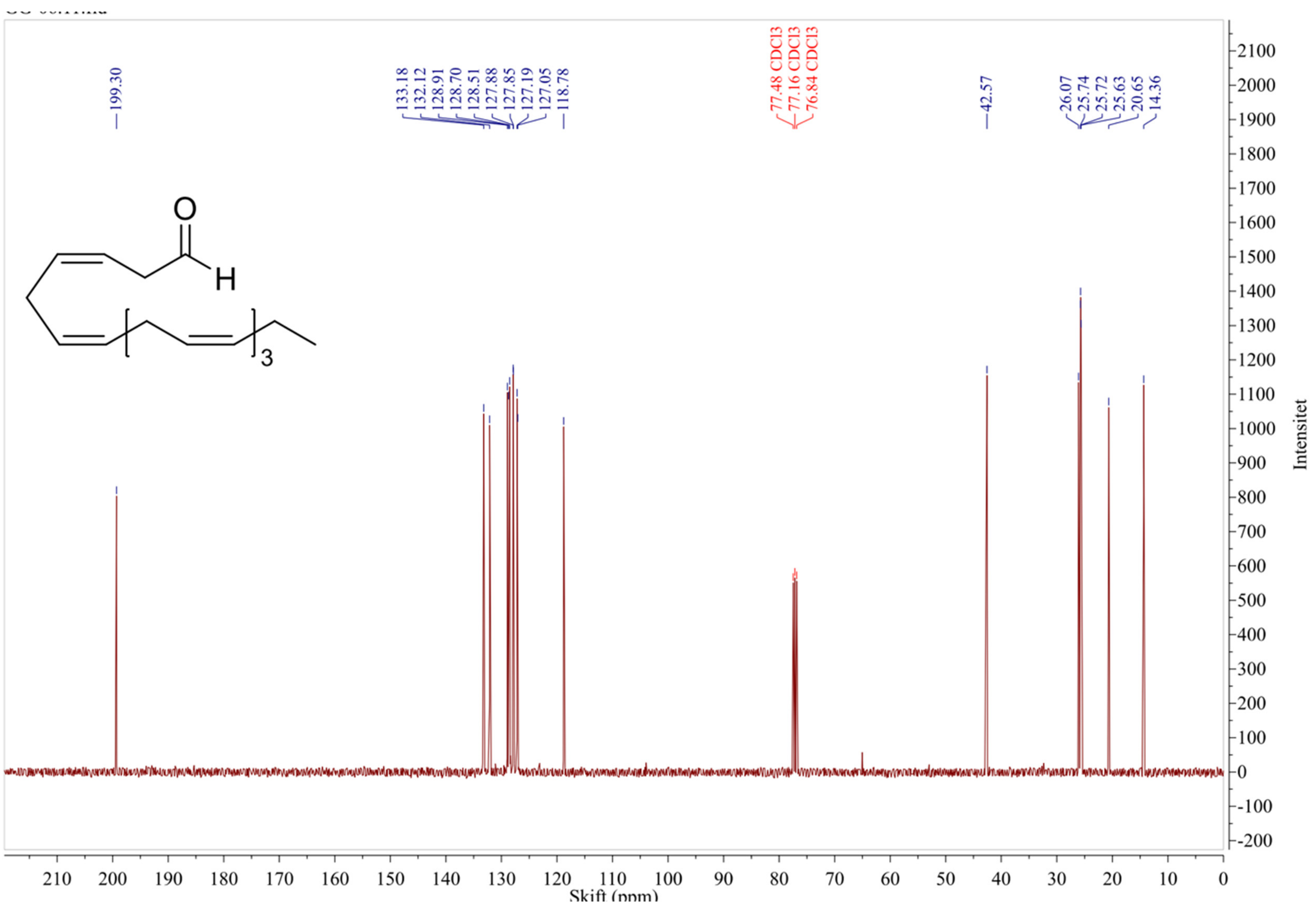

Figure S11 13C NMR spectrum of aldehyde 4

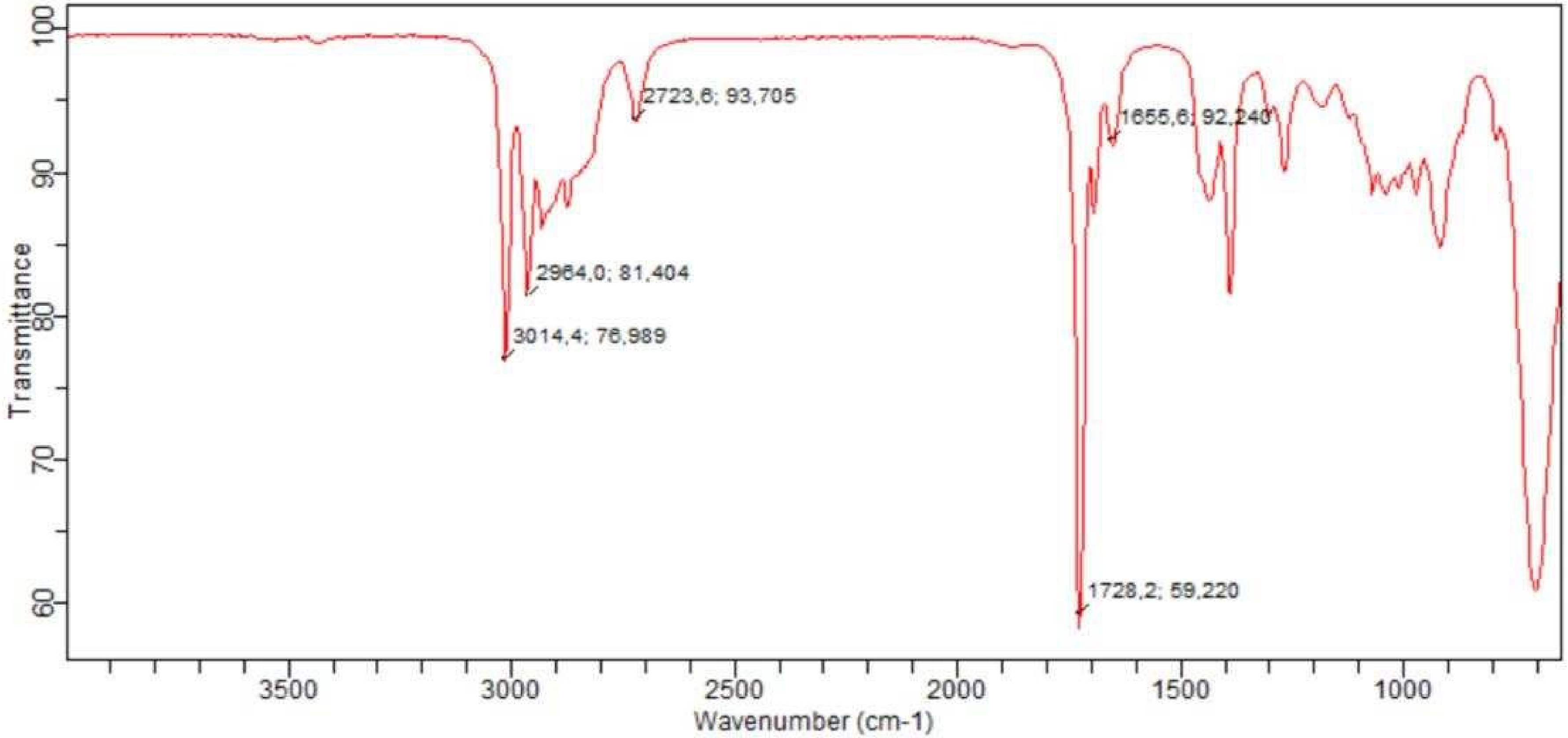

Figure S12 IR spectrum of aldehyde 27

Auxiliary 5b:

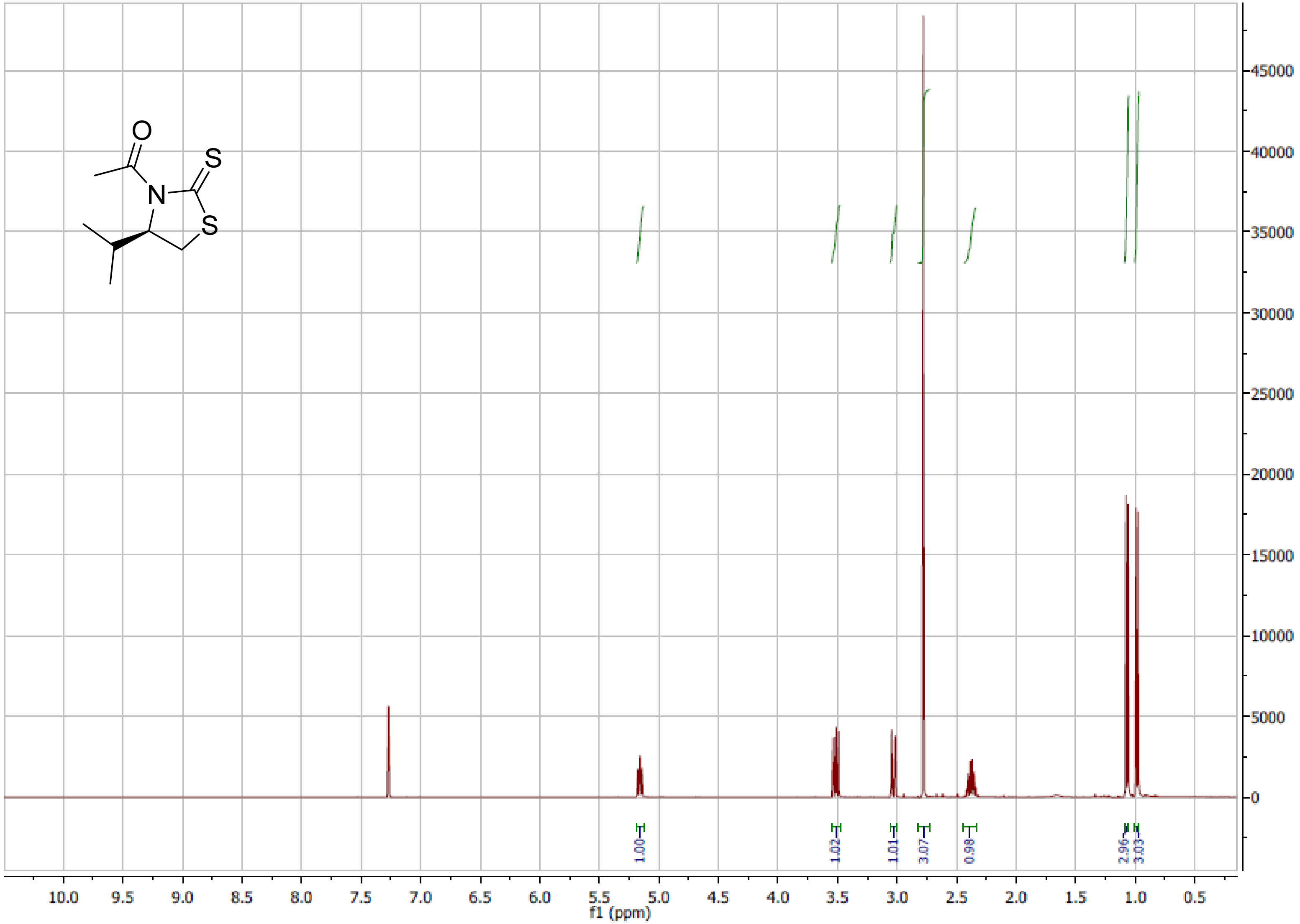

Figure S13 1H NMR spectrum of auxiliary 5b.

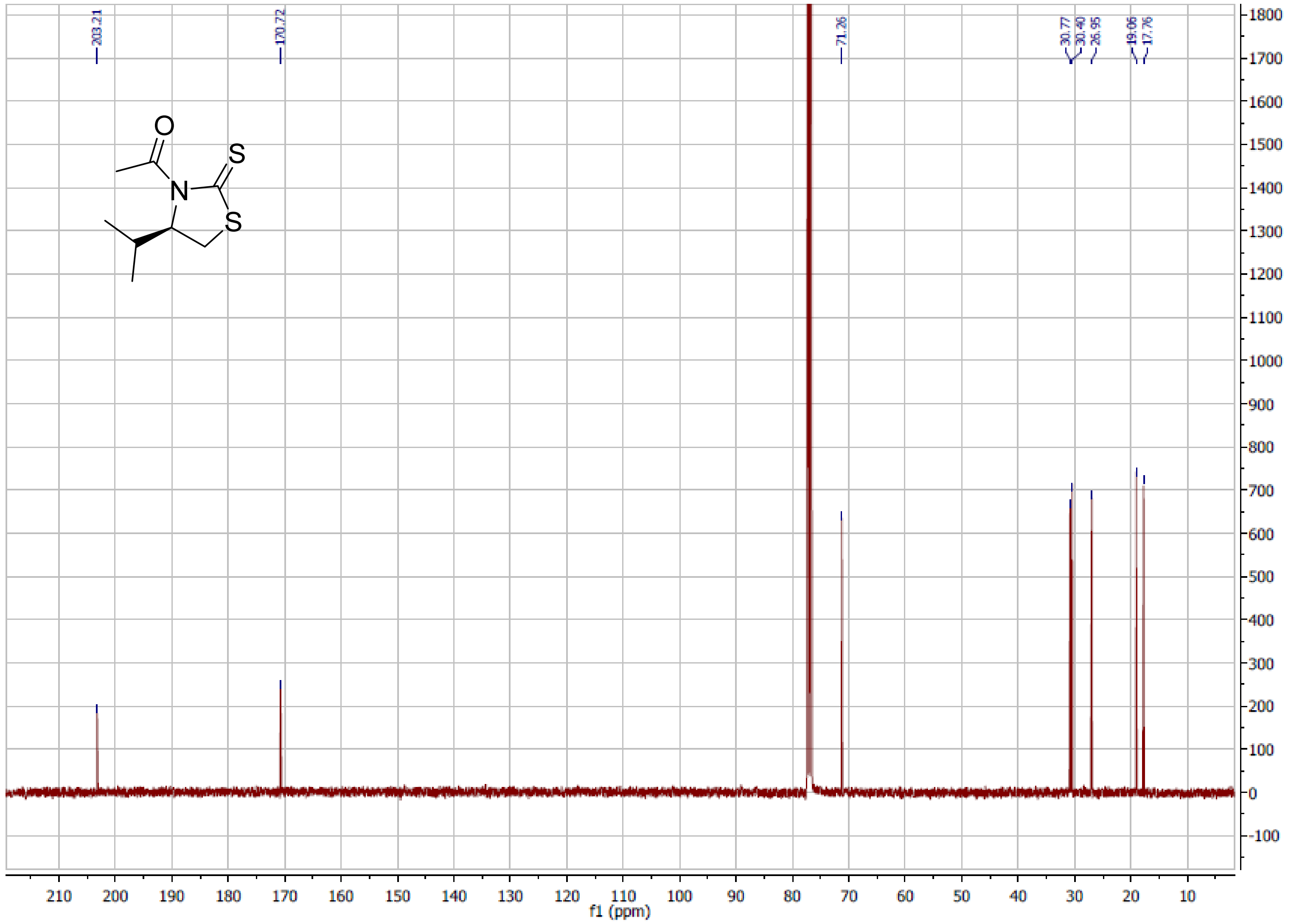

Figure S14 13C NMR spectrum of auxiliary 5b.

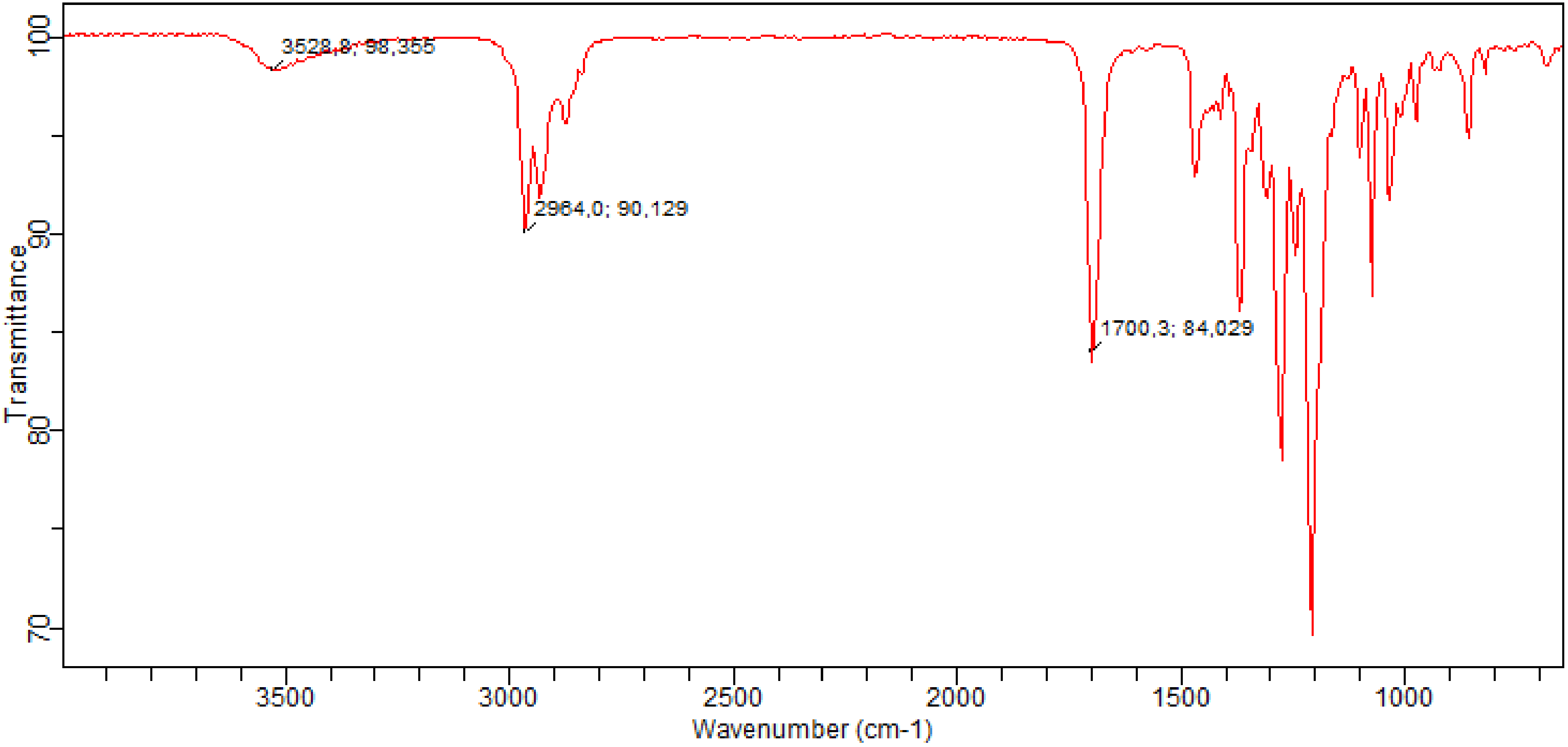

Figure S15 IR spectrum of auxiliary 5b.

Aldol products 9b:

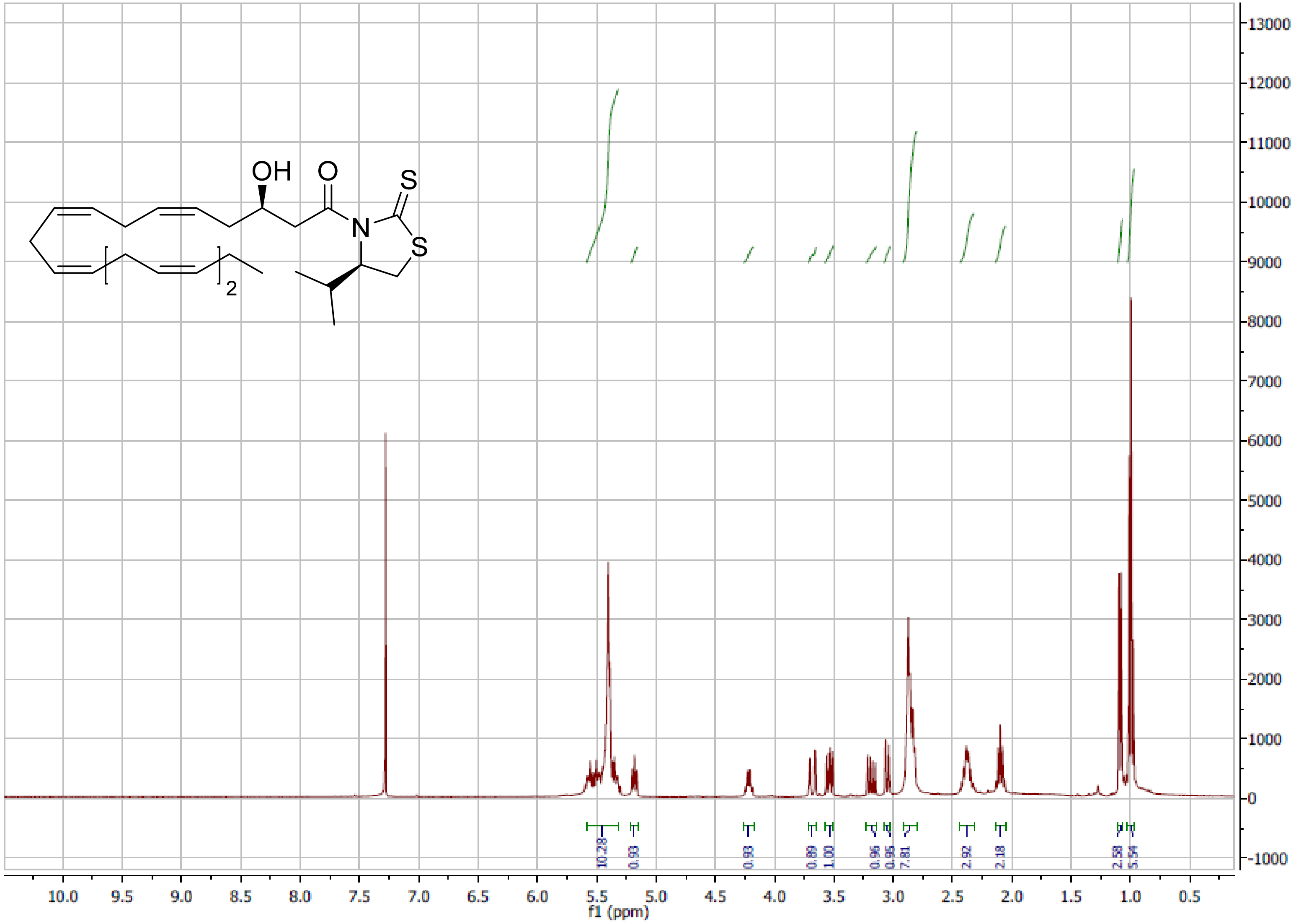

Figure S16 1H NMR spectrum of major aldol product (R)-9b

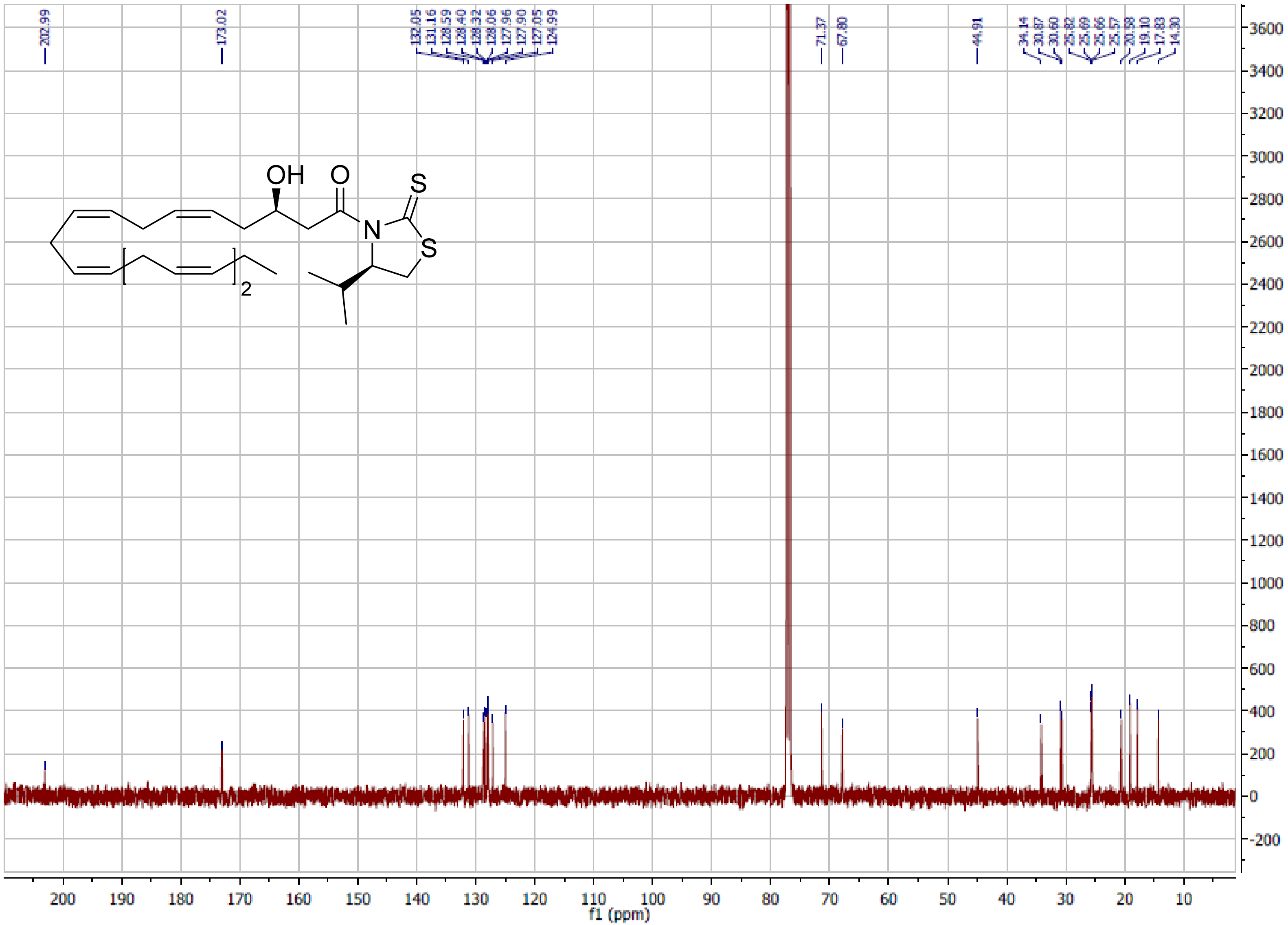

Figure S17 13C NMR spectrum of major aldol product (R)-9b.

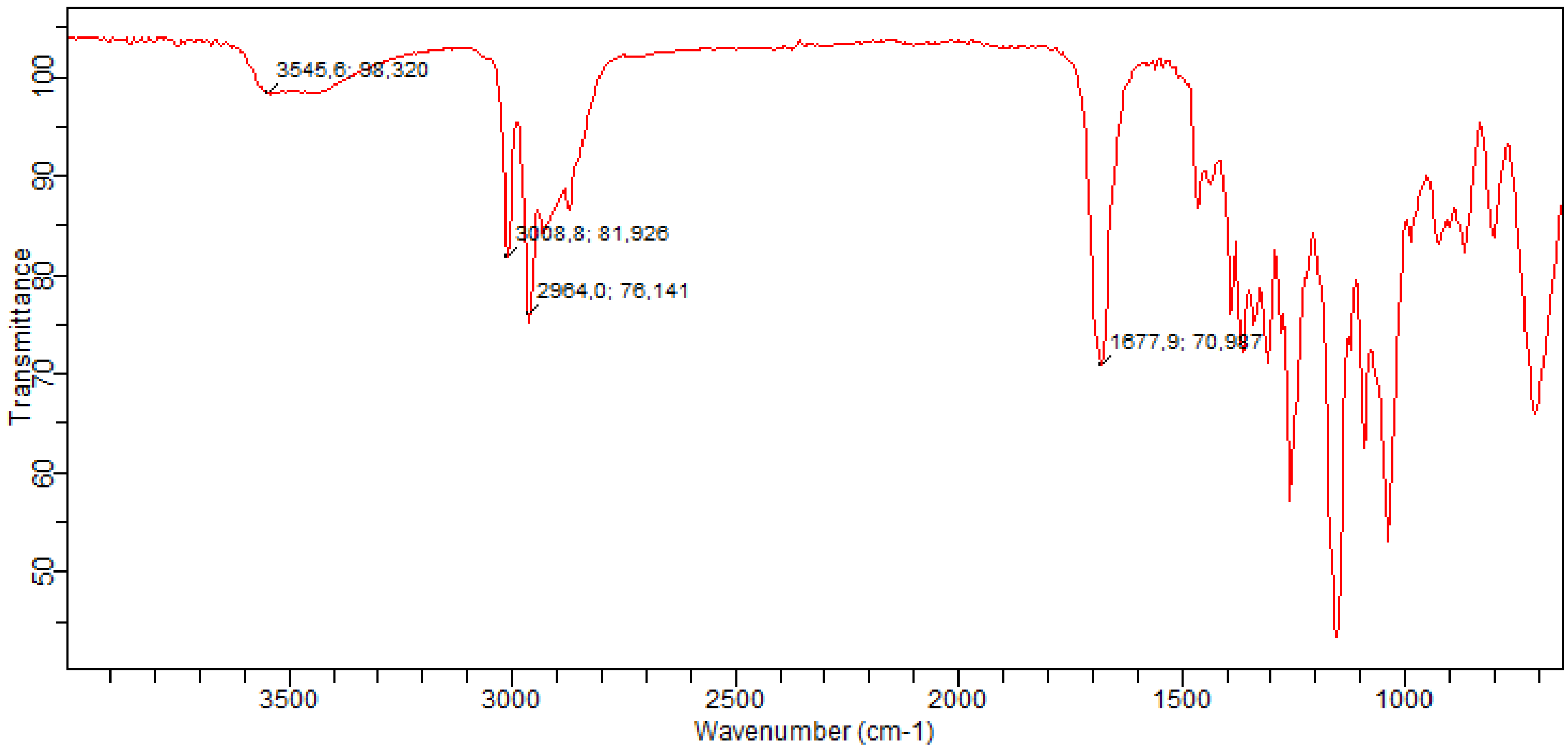

Figure S18 IR spectrum of major aldol product (R)-9b.

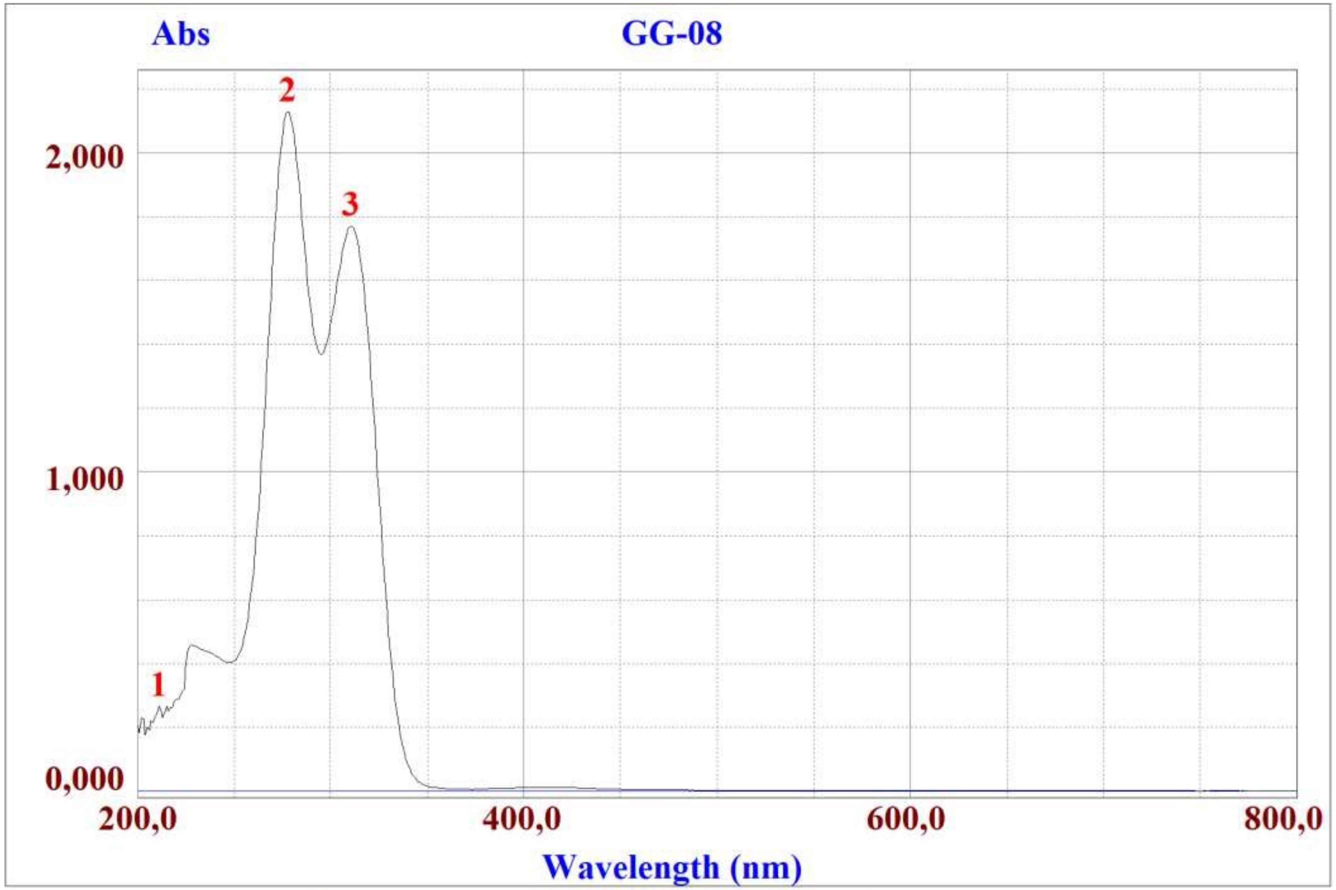

No.	Peak Type	Position	Height	Area	Start	End
1	Peak	211,2	0,267			
2	Peak	277,7	2,129			
3	Peak	310,5	1,770			

Figure S19 UV spectrum of major aldol product (R)-9b.

Elemental Composition Report

Single Mass Analysis

Tolerance = 10.0 PPM / DBE: min = -1.5, max = 50.0
Isotope cluster parameters: Separation = 1.0 Abundance = 1.0%

Monoisotopic Mass, Odd and Even Electron Ions
32 formula(e) evaluated with 1 results within limits (up to 50 closest results for each mass)

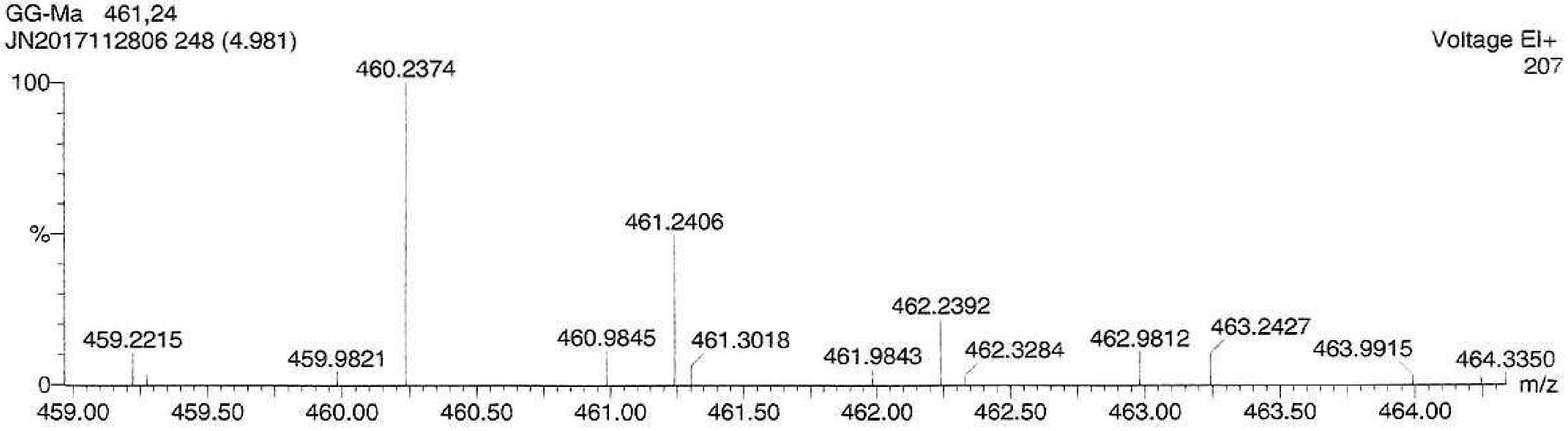

Minimum:				-1.5		
Maximum:		200.0	10.0	50.0		
Mass	Calc. Mass	mDa	PPM	DBE	Score	Formula
461.2406	461.2422	-1.6	-3.5	8.0	1	C26 H39 N O2 S2

Figure S20 MS of major aldol product (R)-9b.

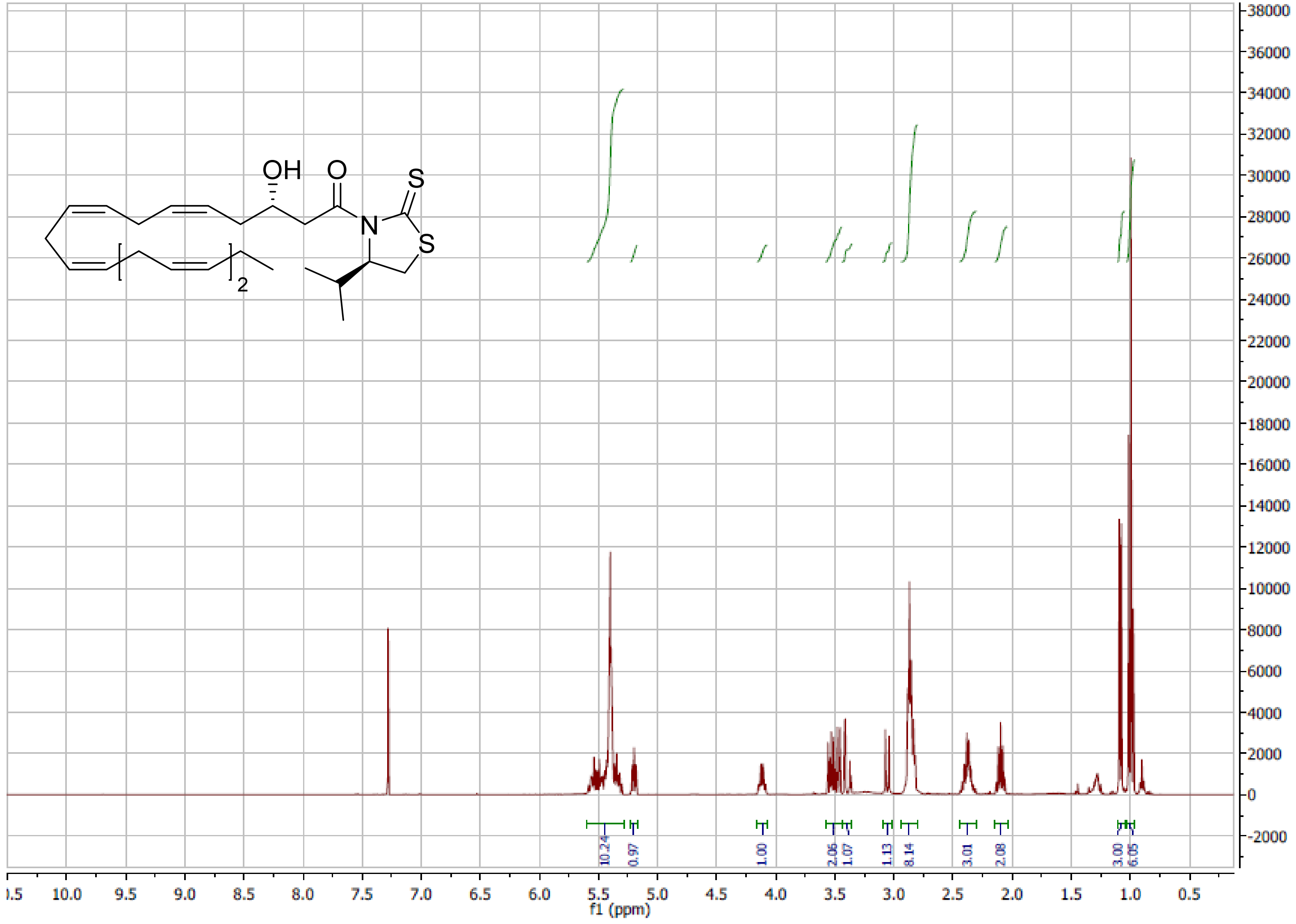

Figure S21 1H NMR spectrum of minor aldol product (S)-9b.

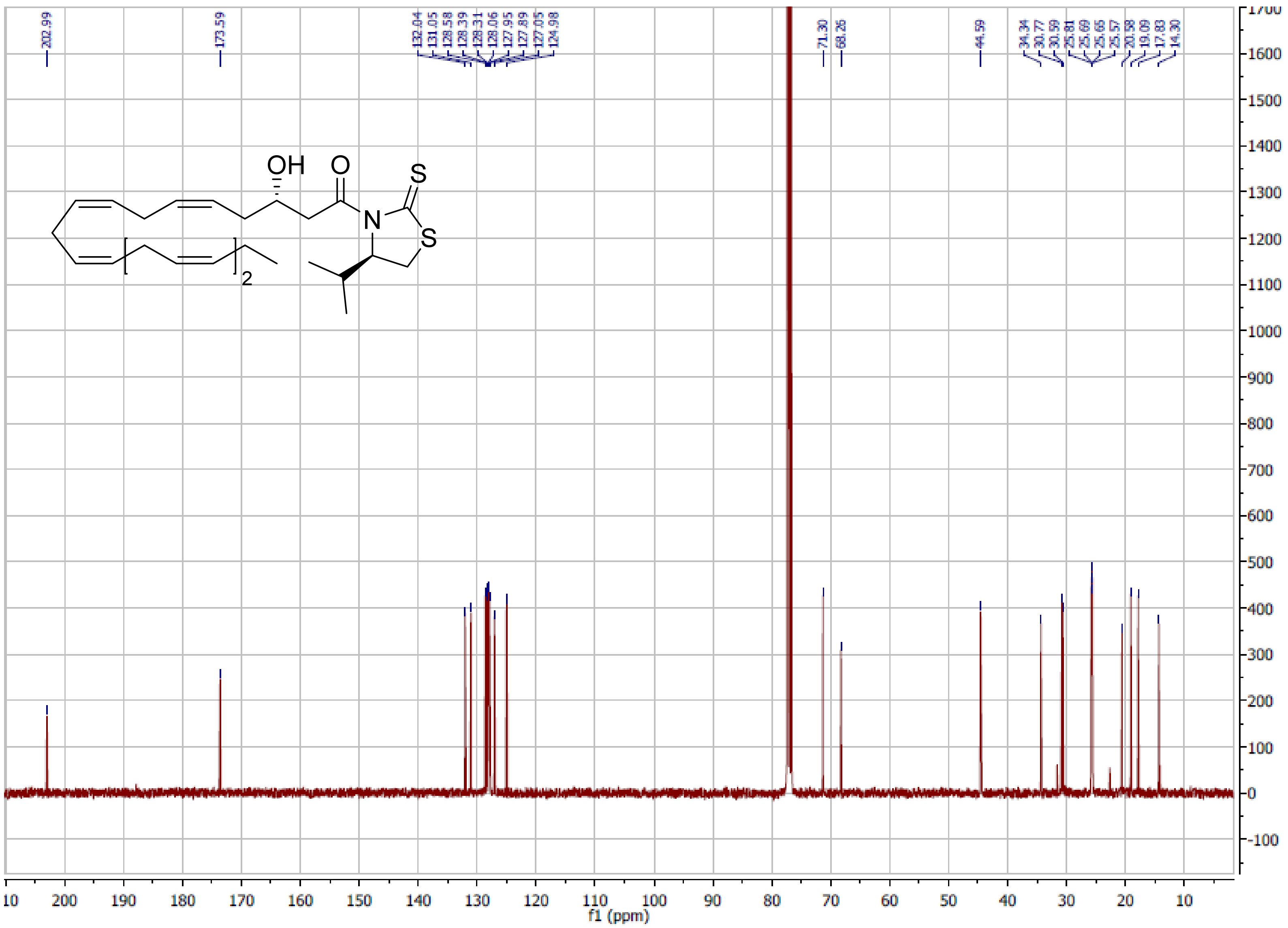

Figure S22 13C NMR spectrum of minor aldol product (S)-9b.

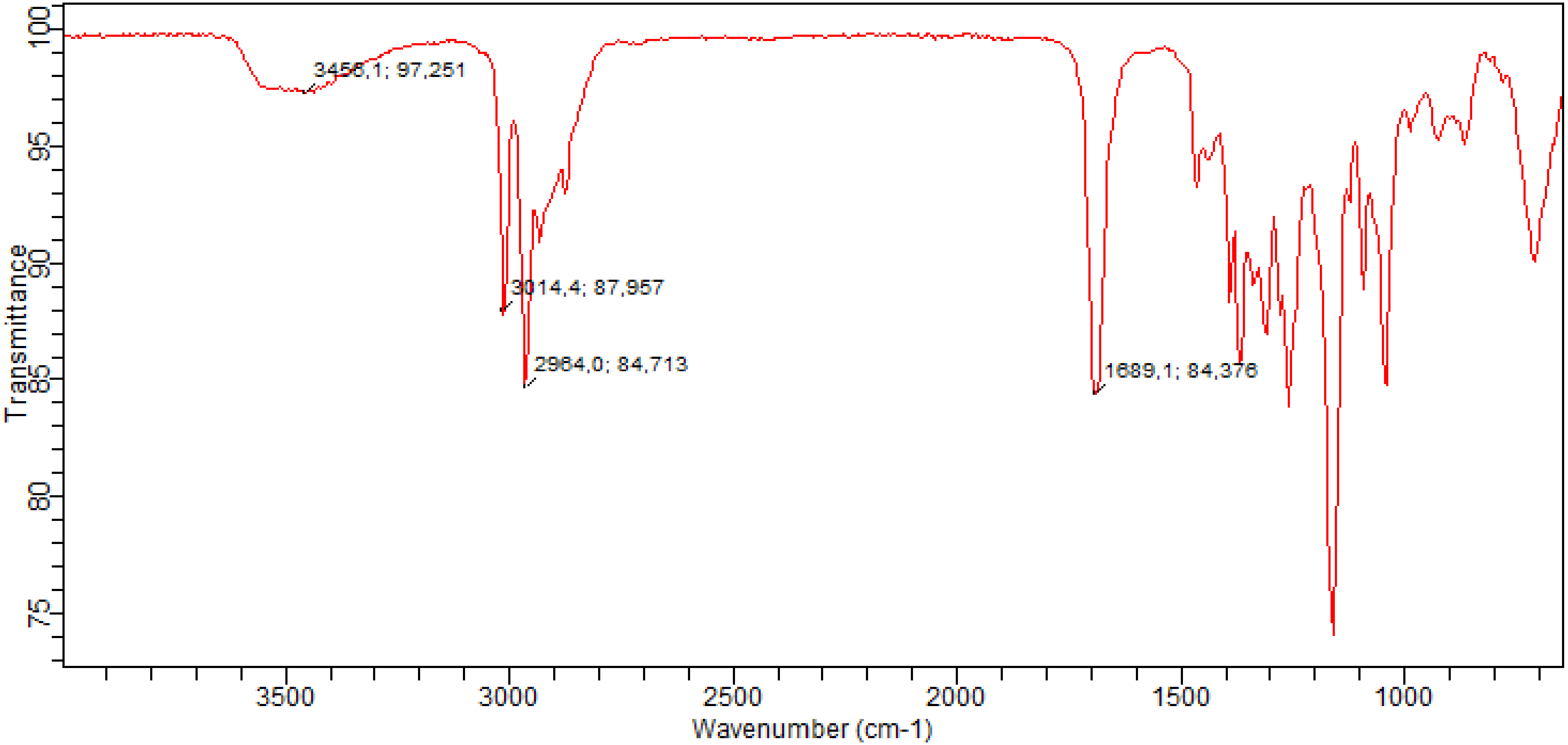

Figure S23 IR spectrum of minor aldol product (S)-9b.

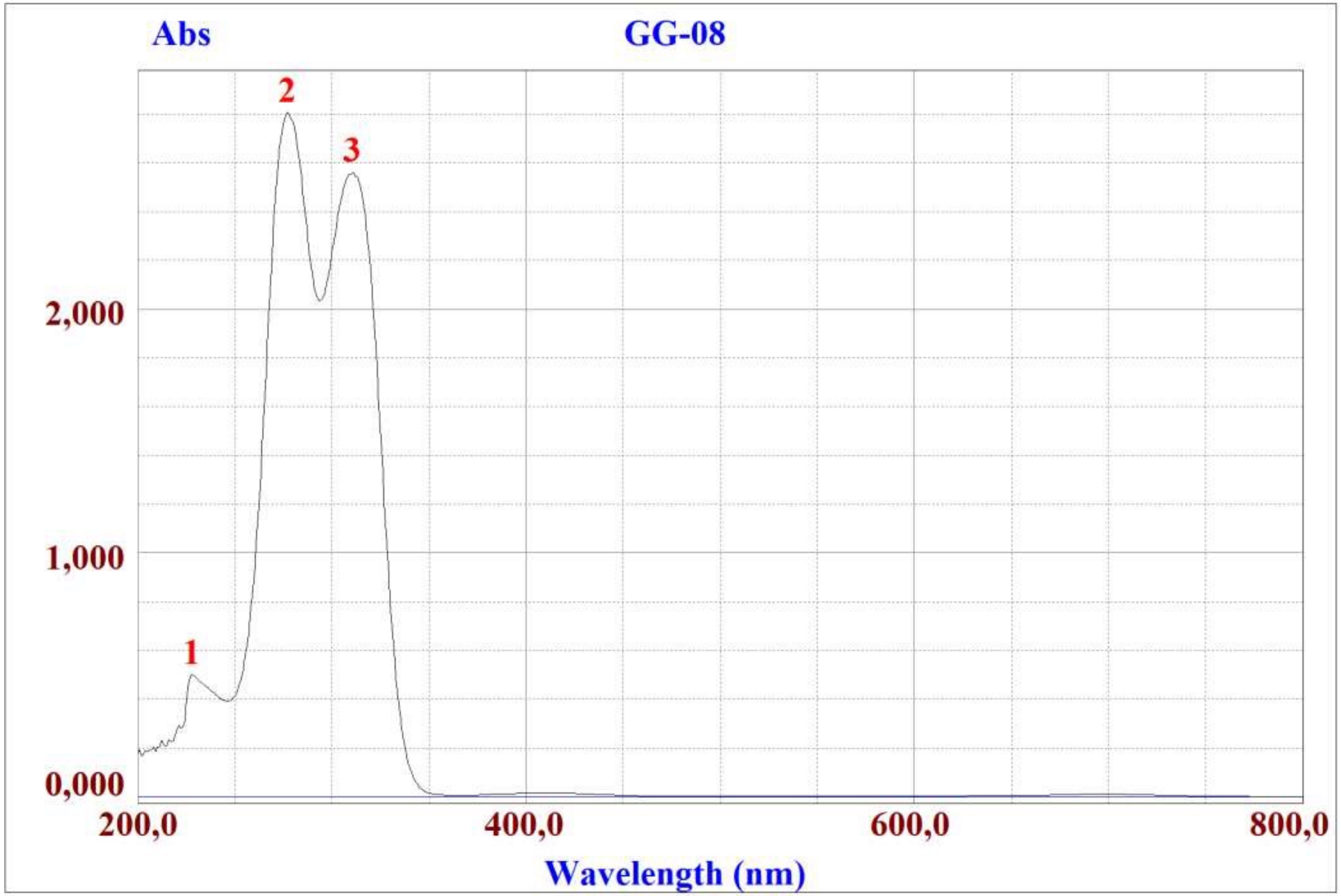

No.	Peak Type	Position	Height	Area	Start	End
1	Peak	227,7	0,502			
2	Peak	277,2	2,808			
3	Peak	310,7	2,560			

Figure S24 UV spectrum of minor aldol product (S)-9b.

Elemental Composition Report

Single Mass Analysis
Tolerance = 10.0 PPM / DBE: min = -1.5, max = 50.0
Isotope cluster parameters: Separation = 1.0 Abundance = 1.0%

Monoisotopic Mass, Odd and Even Electron Ions
32 formula(e) evaluated with 1 results within limits (up to 50 closest results for each mass)

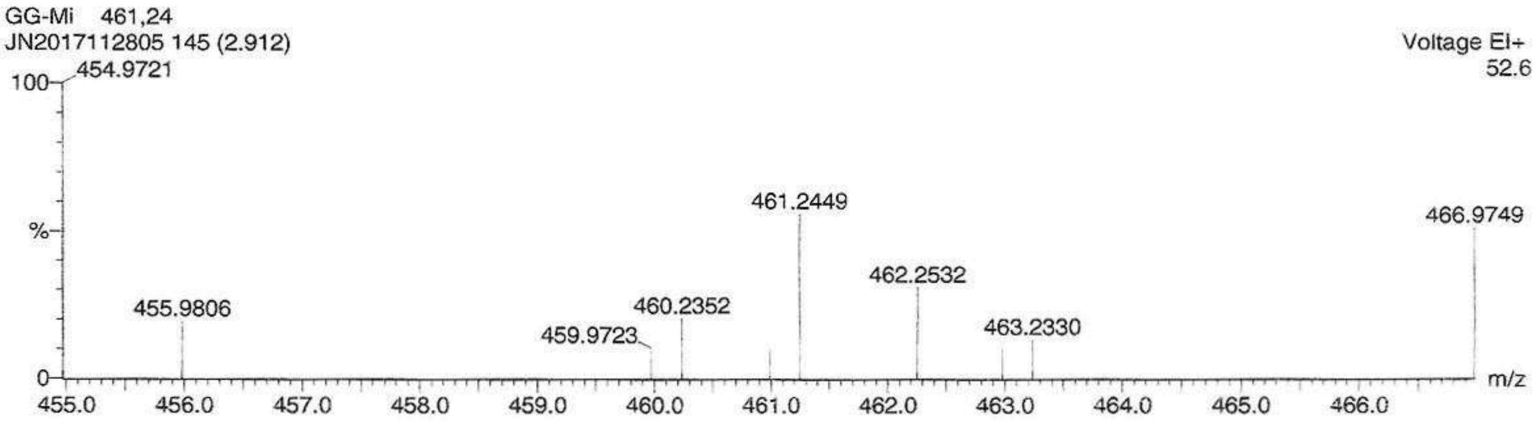

Minimum:				-1.5		
Maximum:		200.0	10.0	50.0		
Mass	Calc. Mass	mDa	PPM	DBE	Score	Formula
461.2449	461.2422	2.7	5.8	8.0	1	C26 H39 N O2 S2

Figure S25 HRMS of minor aldol product (S)-9b.

TBS protected aldol product 10:

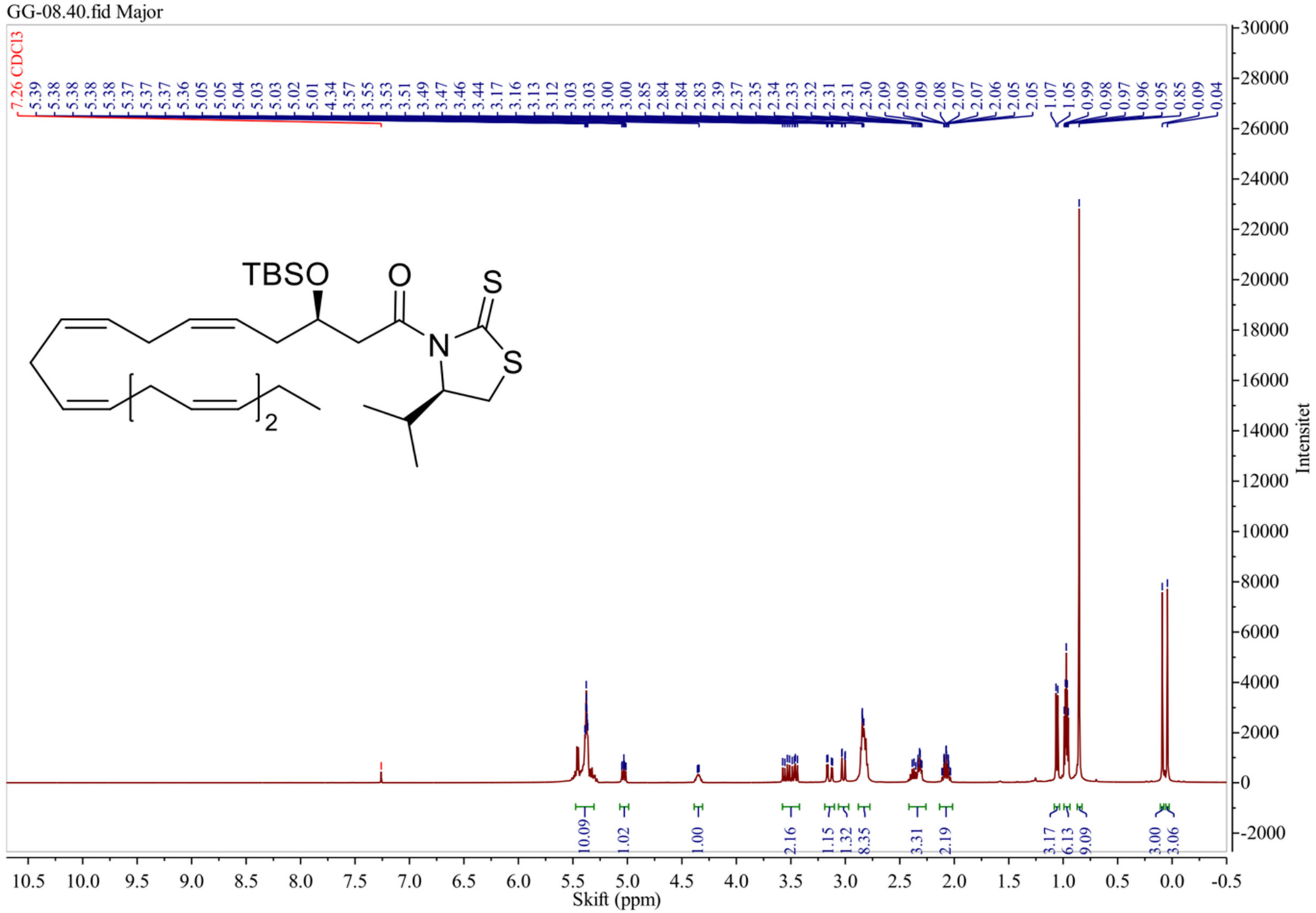

Figure S26 1H NMR spectrum of TBS protected aldol product

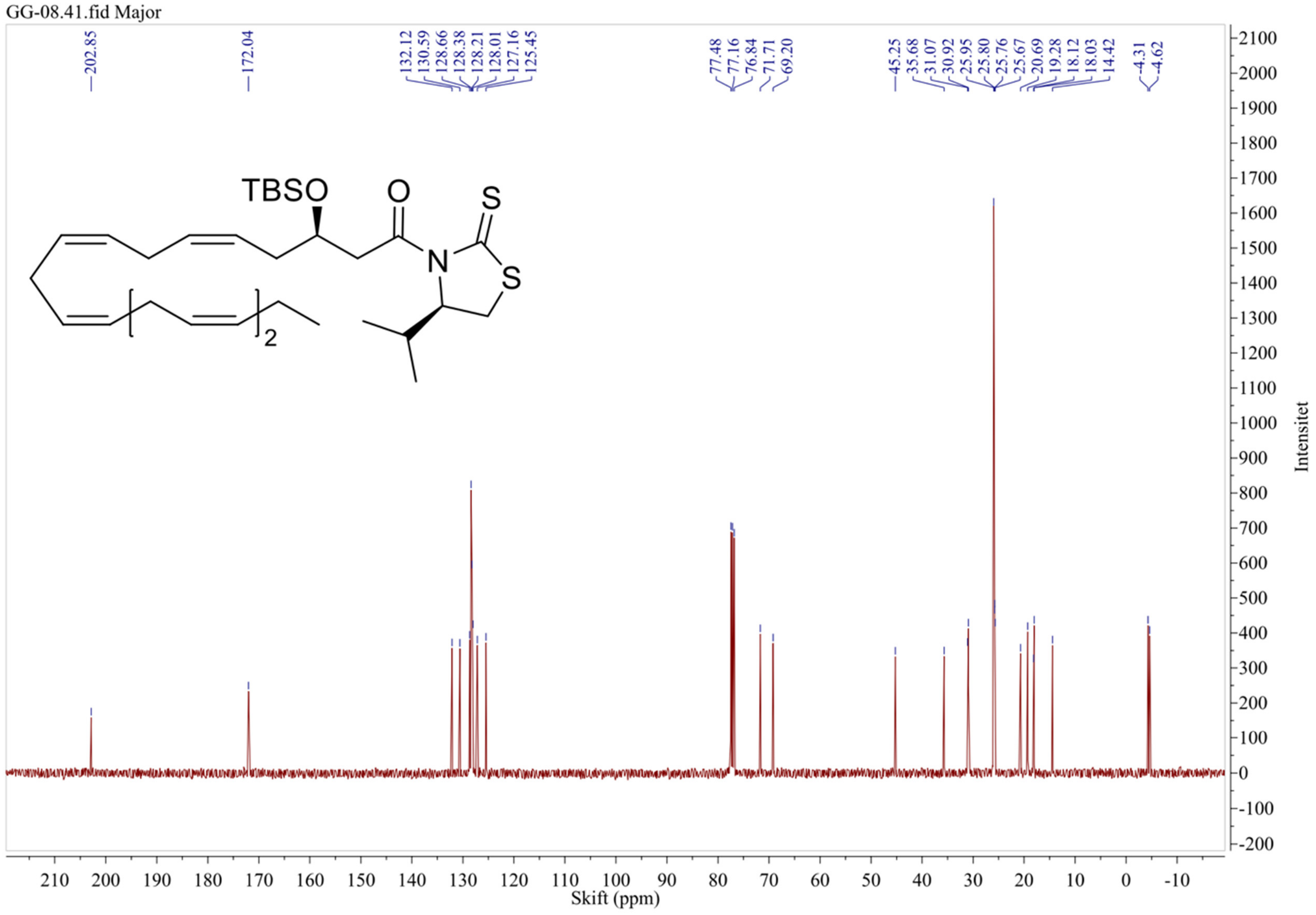

Figure S27 13C NMR spectrum of TBS protected aldol product

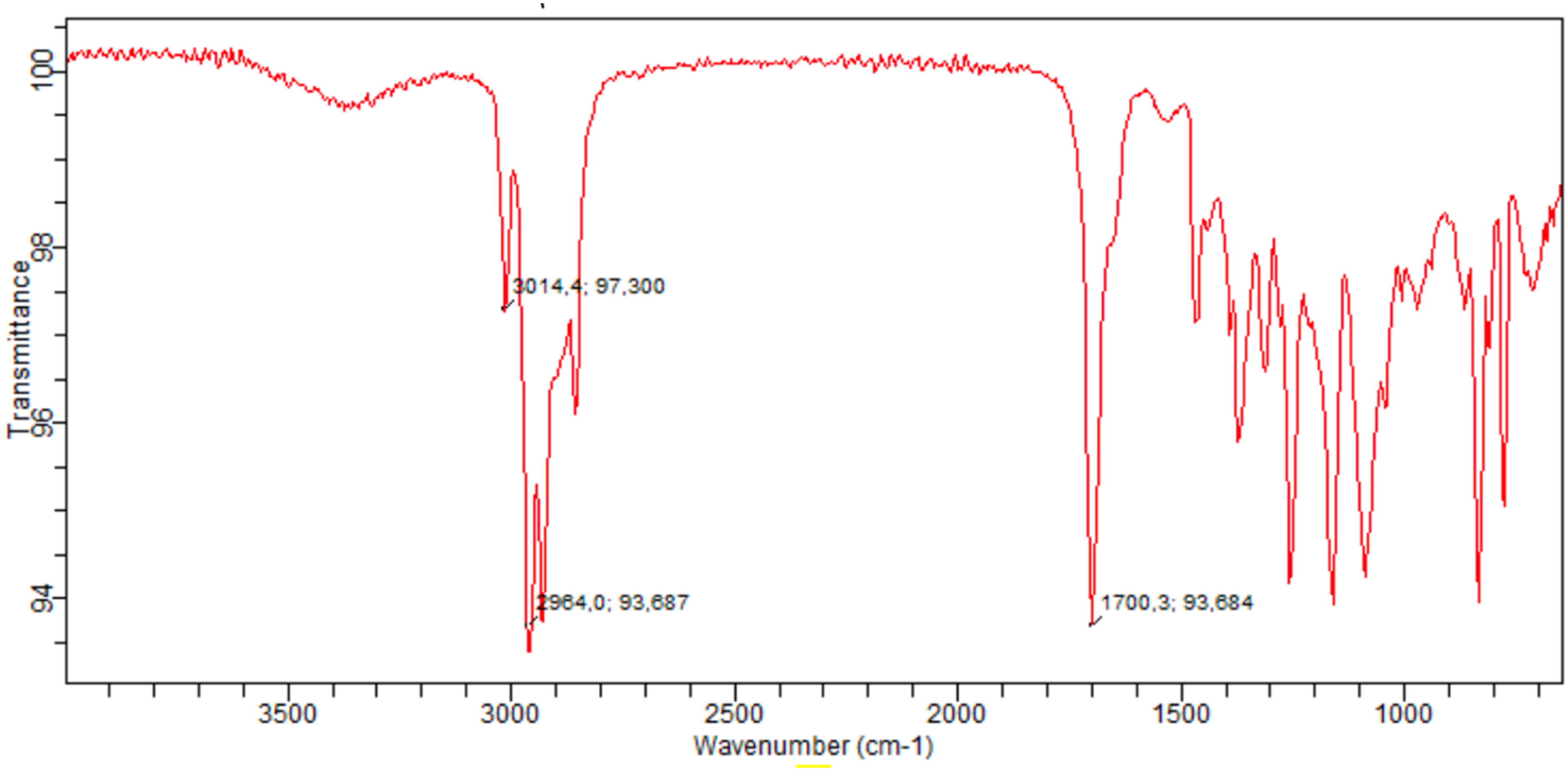

Figure S28 IR spectrum of TBS protected aldol product

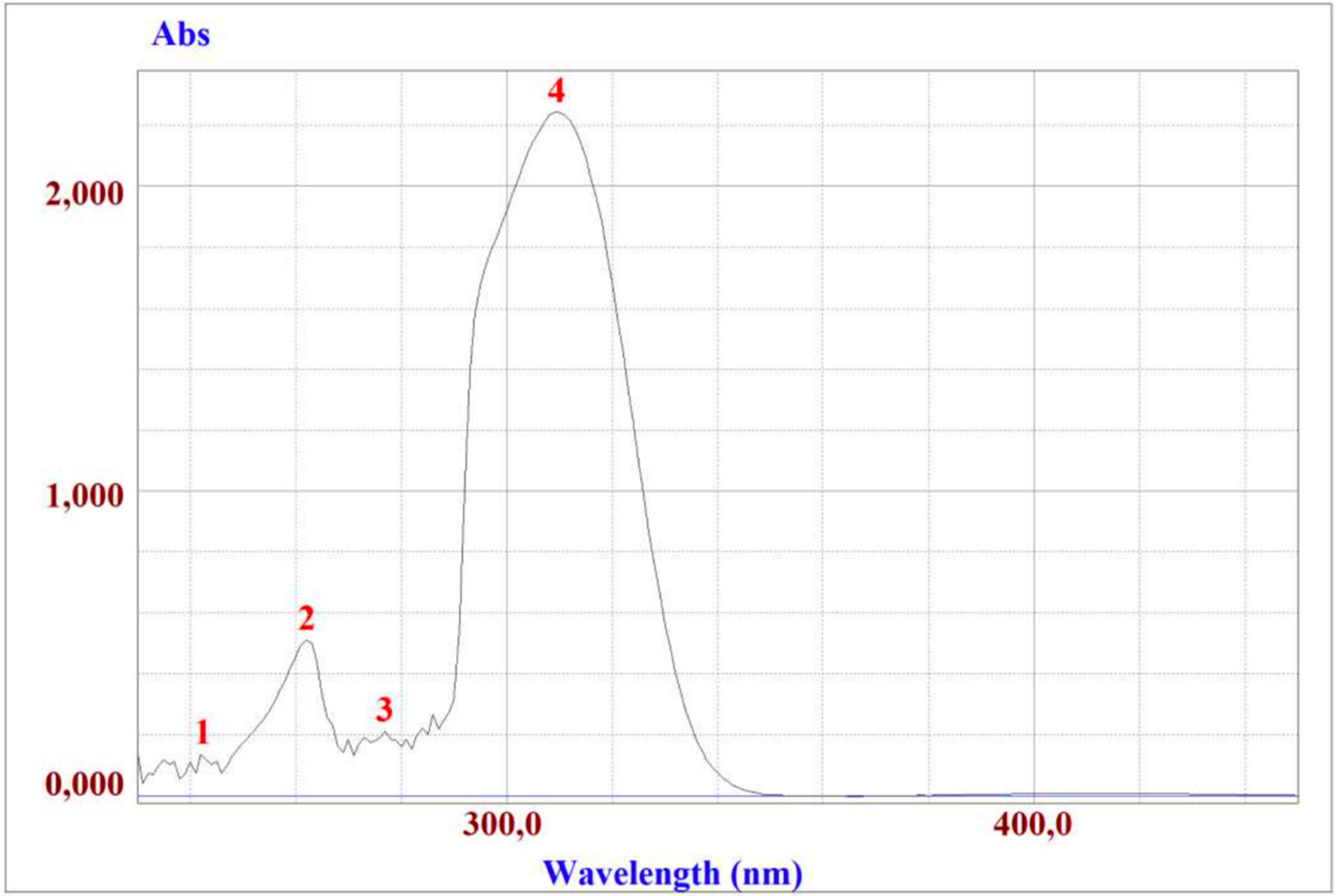

No.	Peak Type	Position	Height
1	Peak	242,3	0,136
2	Peak	262,2	0,513
3	Peak	276,9	0,212
4	Peak	309,5	2,244

Figure S29 UV spectrum of TBS protected aldol product

Elemental Composition Report

Single Mass Analysis

Tolerance = 10.0 PPM / DBE: min = -1.5, max = 50.0
Isotope cluster parameters: Separation = 1.0 Abundance = 1.0%

Monoisotopic Mass, Odd and Even Electron Ions
572 formula(e) evaluated with 9 results within limits (up to 50 closest results for each mass)

GG-15
DEMHL2018022808 133 (2.669)

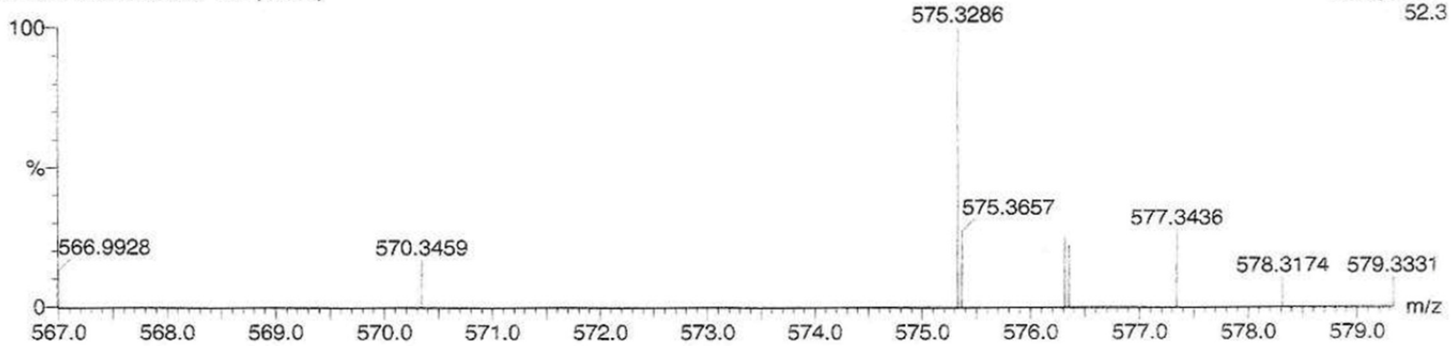

Minimum:				-1.5		
Maximum:		200.0	10.0	50.0		
Mass	Calc. Mass	mDa	PPM	DBE	Score	Formula
575.3286	575.3287	-0.1	-0.2	8.0	3	C32 H53 N O2 S2 Si
	575.3281	0.5	0.9	9.0	6	C32 H49 N O6 S
	575.3260	2.6	4.5	3.5	2	C29 H55 O5 S2 Si
	575.3314	-2.8	-4.9	22.5	9	C43 H43 O
	575.3314	-2.8	-4.9	4.0	1	C29 H53 N O6 S2
	575.3256	3.0	5.3	13.0	5	C36 H49 N O S2
	575.3253	3.3	5.7	13.0	7	C35 H49 N O2 S Si
	575.3247	3.9	6.8	14.0	8	C35 H45 N O6
	575.3229	5.7	9.9	8.5	4	C33 H51 O4 S2

Figure S30 MS of TBS protected aldol product

TBS protected ethyl ester 11:

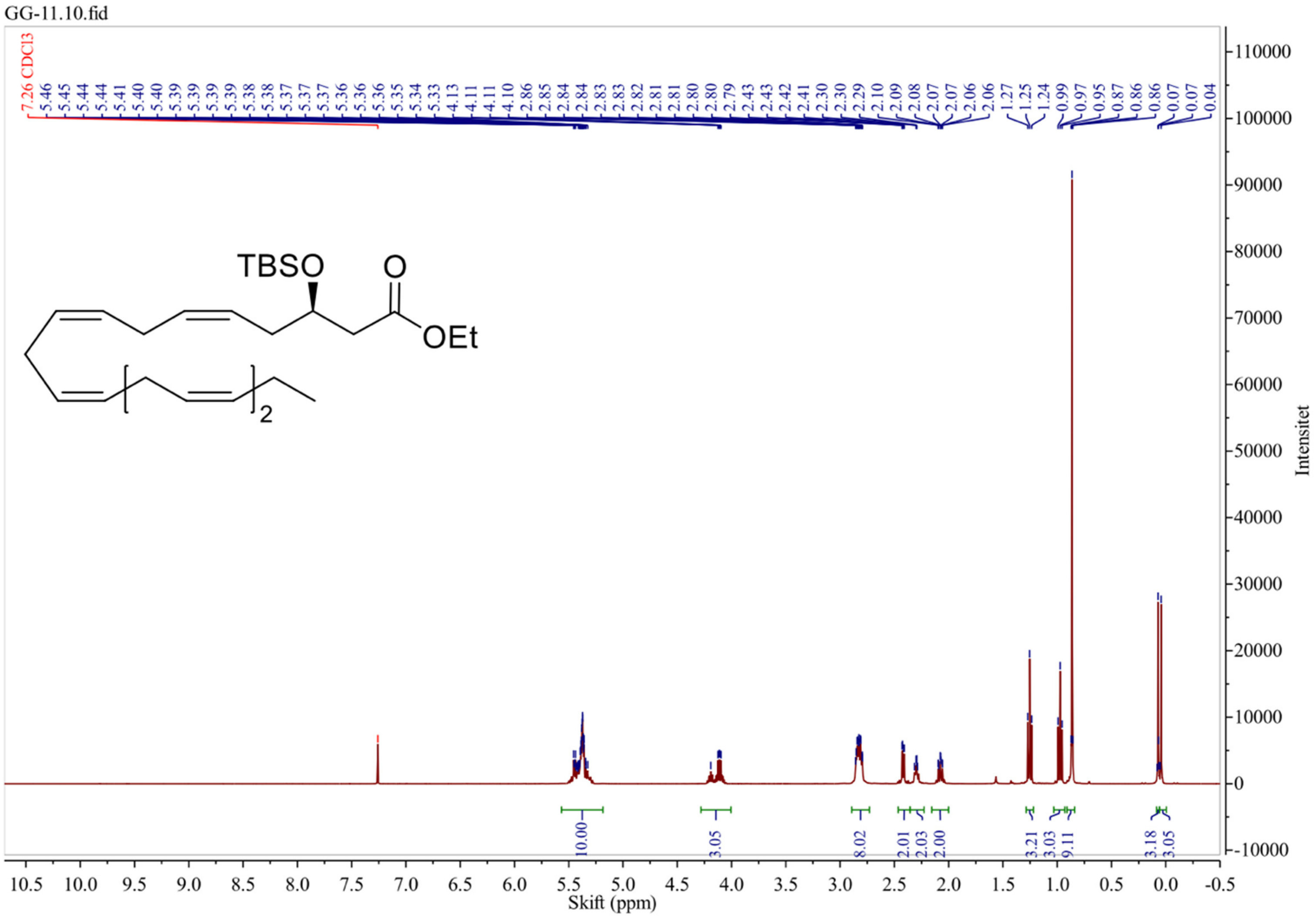

Figure S31 1H NMR spectrum of TBS protected ethyl ester.

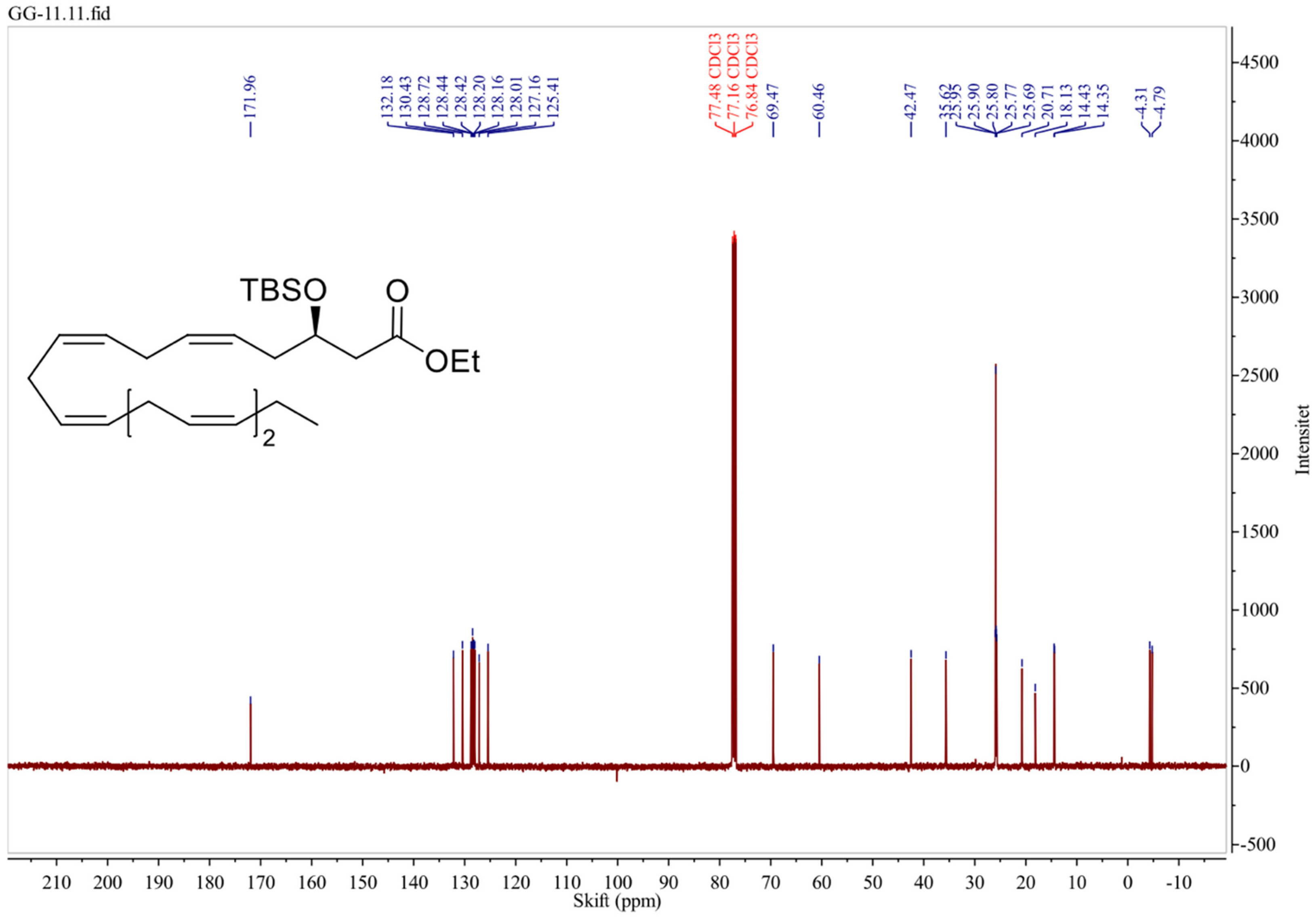

Figure S32 ^{13}C NMR spectrum of TBS protected ethyl ester.

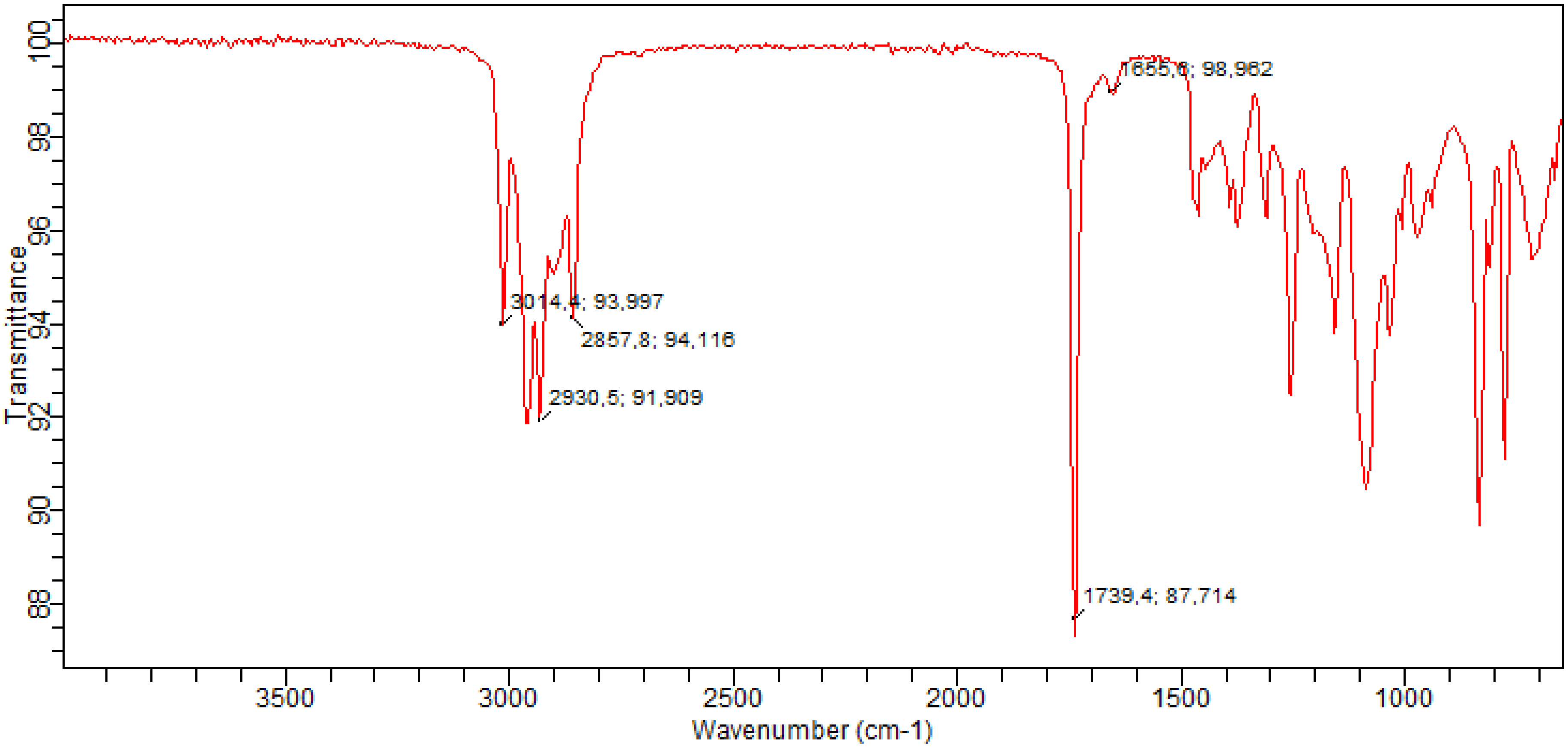

Figure S33 IR spectrum of TBS protected ethyl ester.

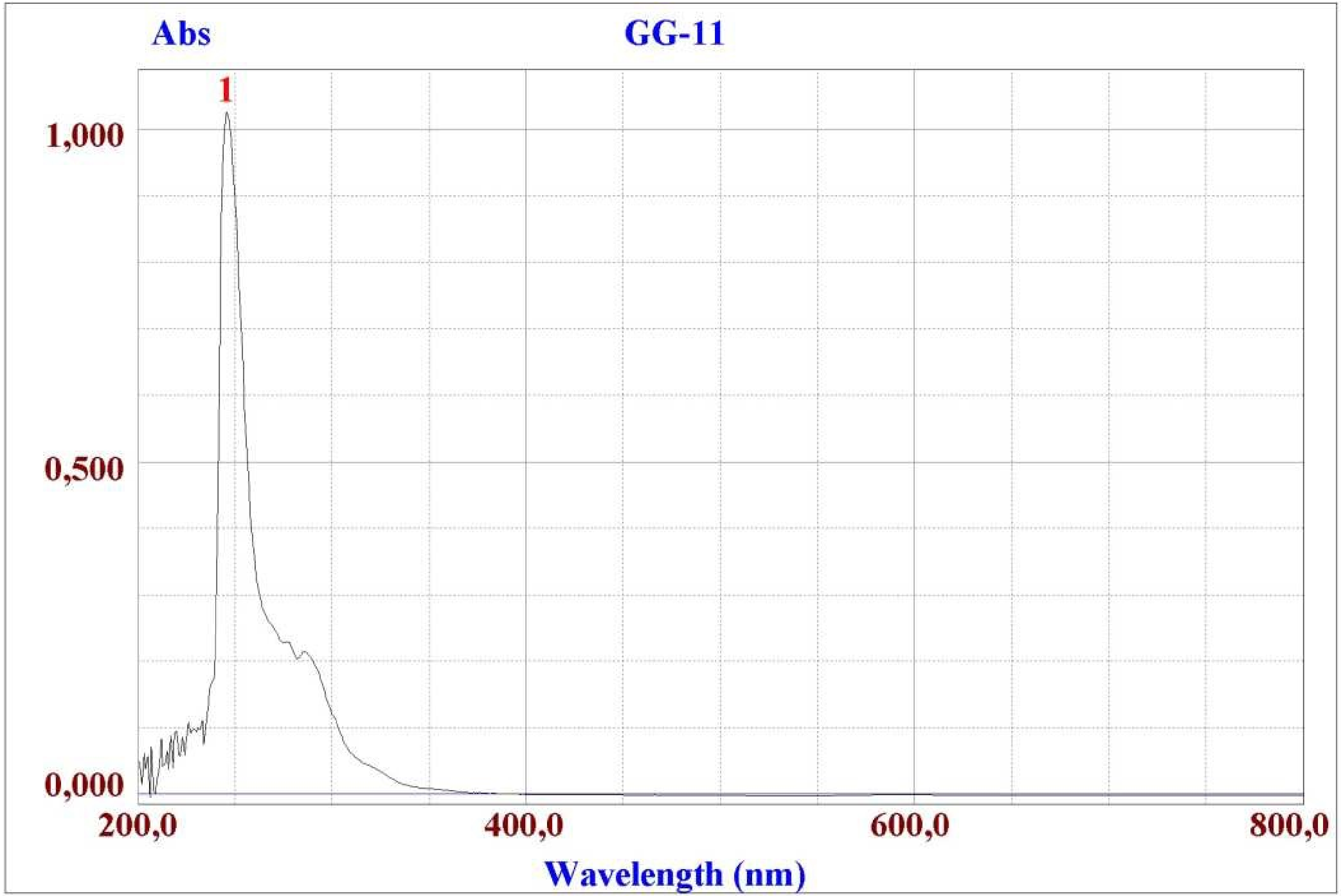

No.	Peak Type	Position	Height
1	Peak	245,8	1,027

Figure S34 UV spectrum of TBS protected ethyl ester.

Elemental Composition Report

Single Mass Analysis

Tolerance = 10.0 PPM / DBE: min = -1.5, max = 50.0
Isotope cluster parameters: Separation = 1.0 Abundance = 1.0%

Monoisotopic Mass, Odd and Even Electron Ions
83 formula(e) evaluated with 2 results within limits (up to 50 closest results for each mass)

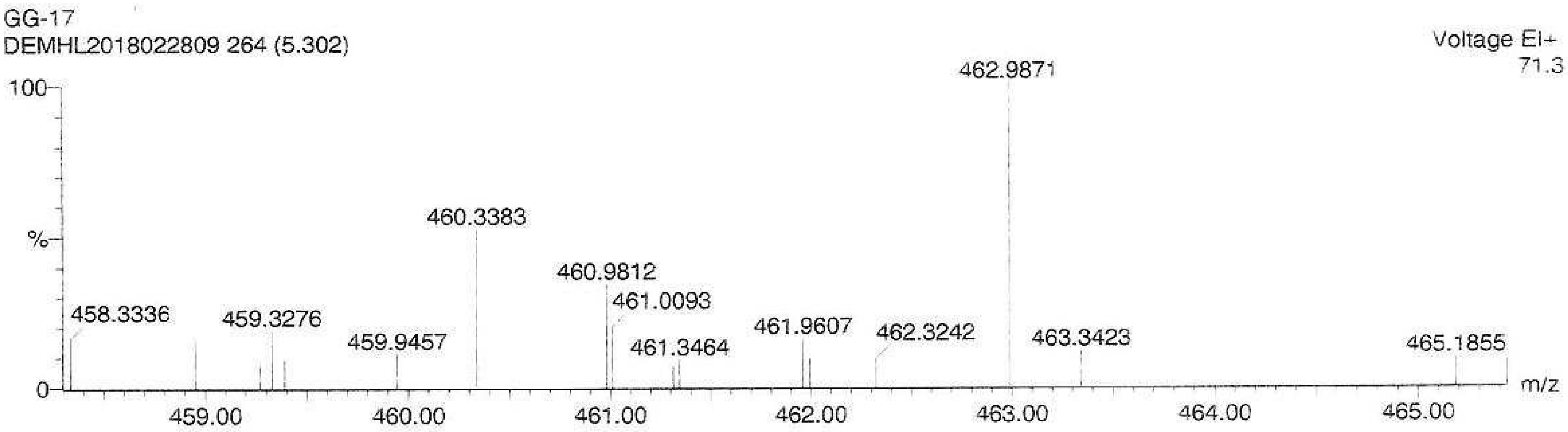

Minimum:				-1.5		
Maximum:		200.0	10.0	50.0		
Mass	Calc. Mass	mDa	PPM	DBE	Score	Formula
460.3383	460.3373	1.0	2.2	6.0	2	C28 H48 O3 Si
	460.3341	4.2	9.1	11.0	1	C32 H44 O2

Figure S35 HRMS of TBS-protected ethyl ester 11

3(R)-HEPE (2):

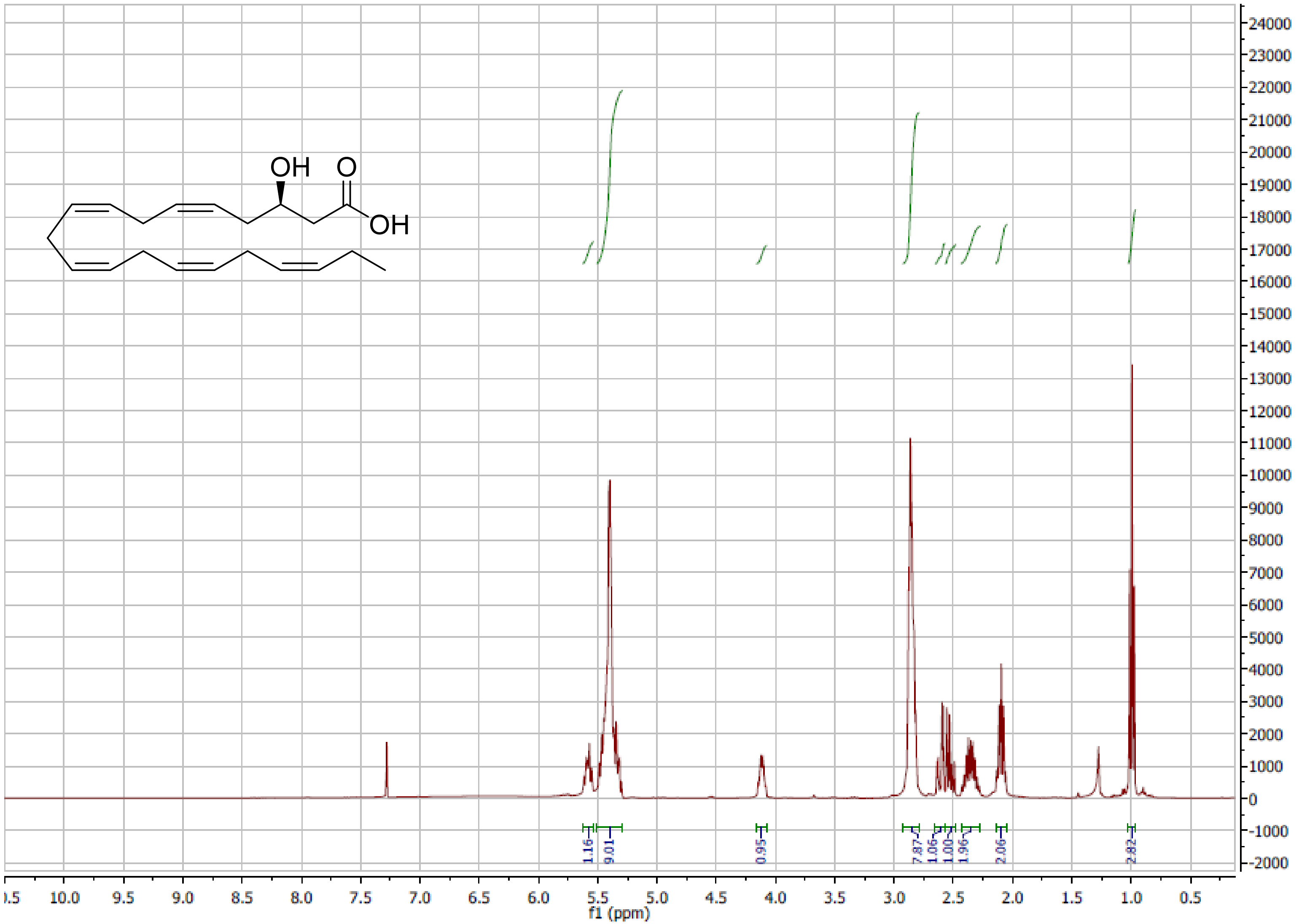

Figure S36 ^{1}H NMR spectrum of 3(R)-HEPE (2).

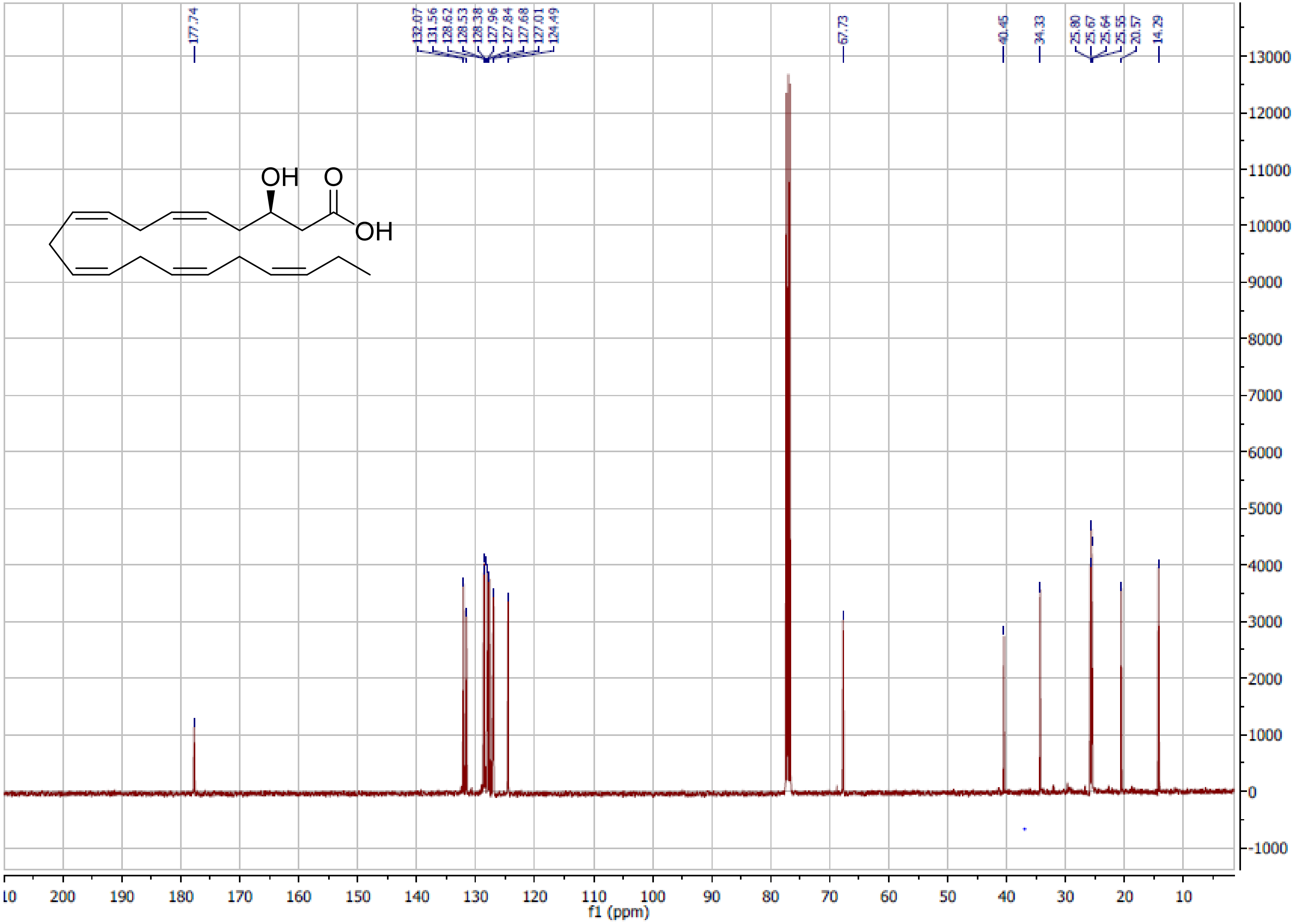

Figure S37 ^{13}C NMR spectrum of 3(R)-HEPE (2).

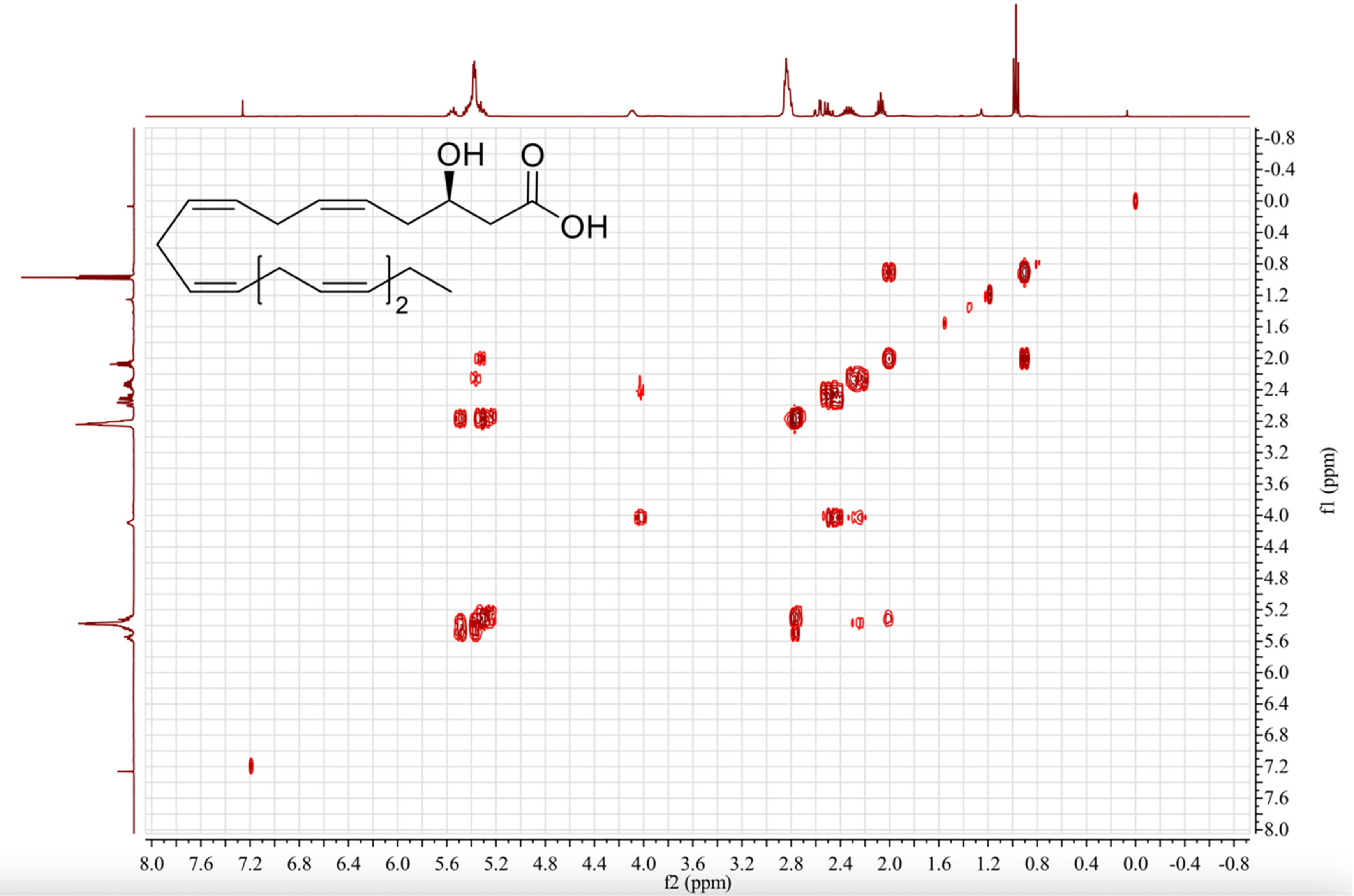

Figure S38 COSY spectrum of 3(R)-HEPE (2).

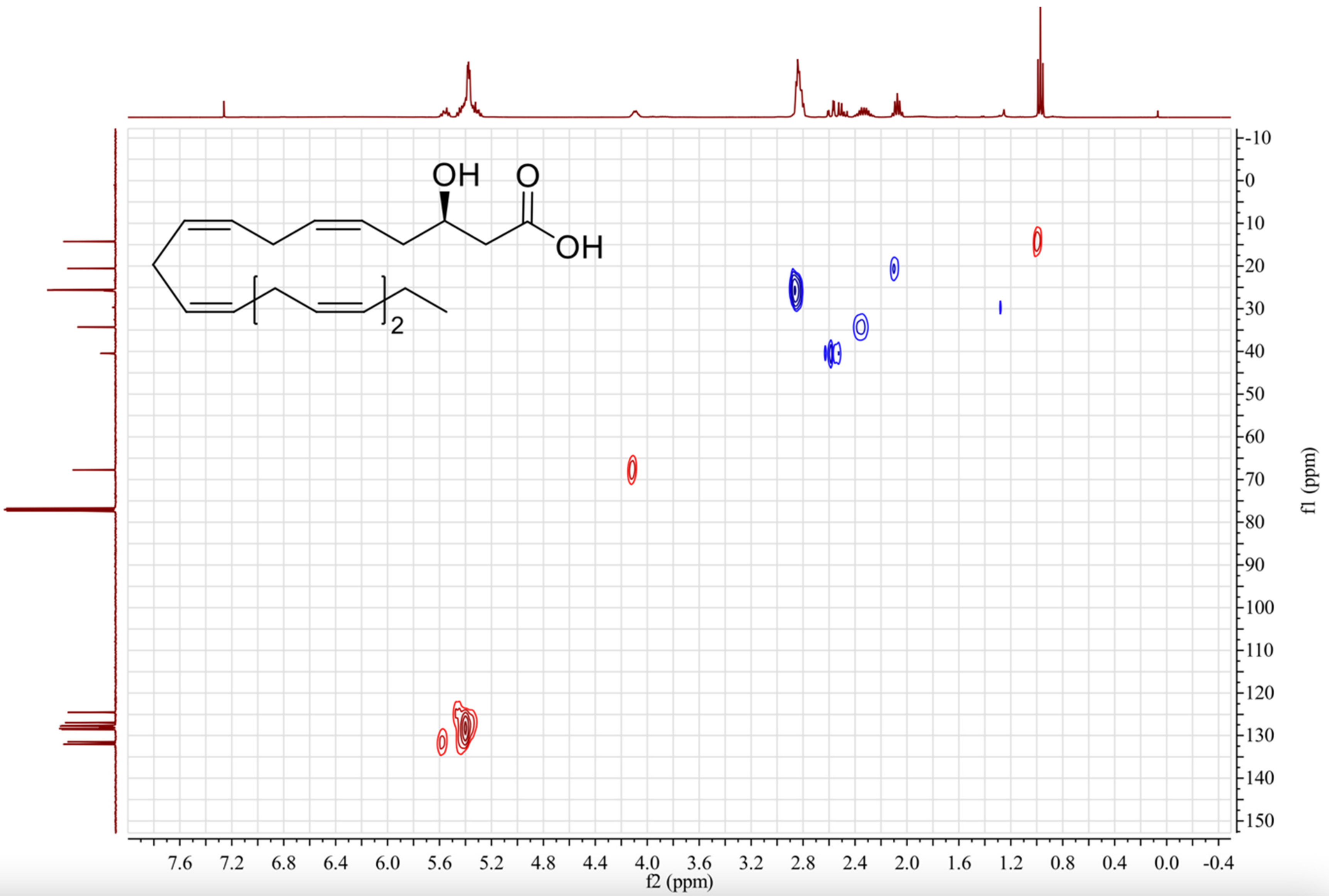

Figure S39 HMBC spectrum of 3(R)-HEPE (2).

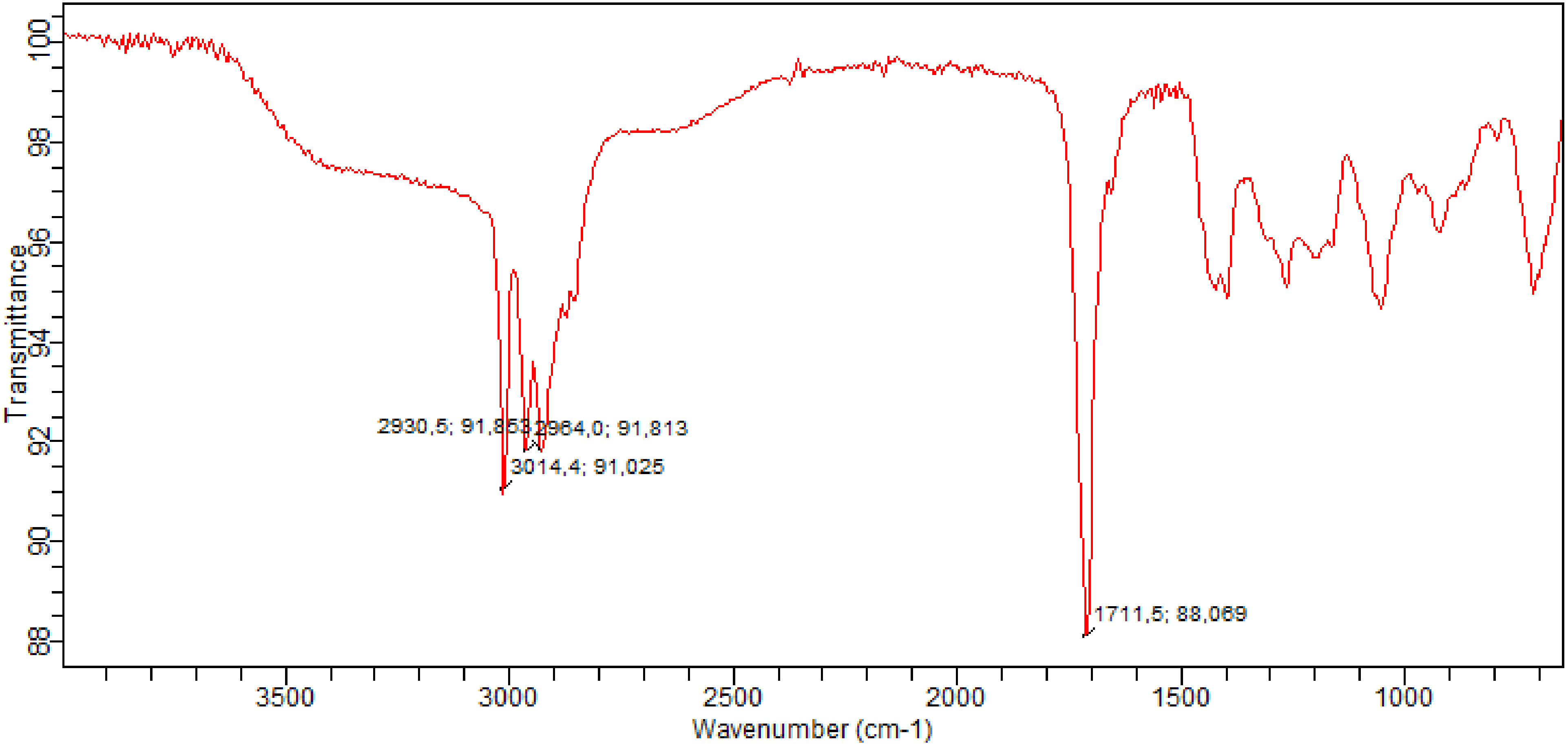

Figure S40 IR spectrum of 3(R)-HEPE (2).

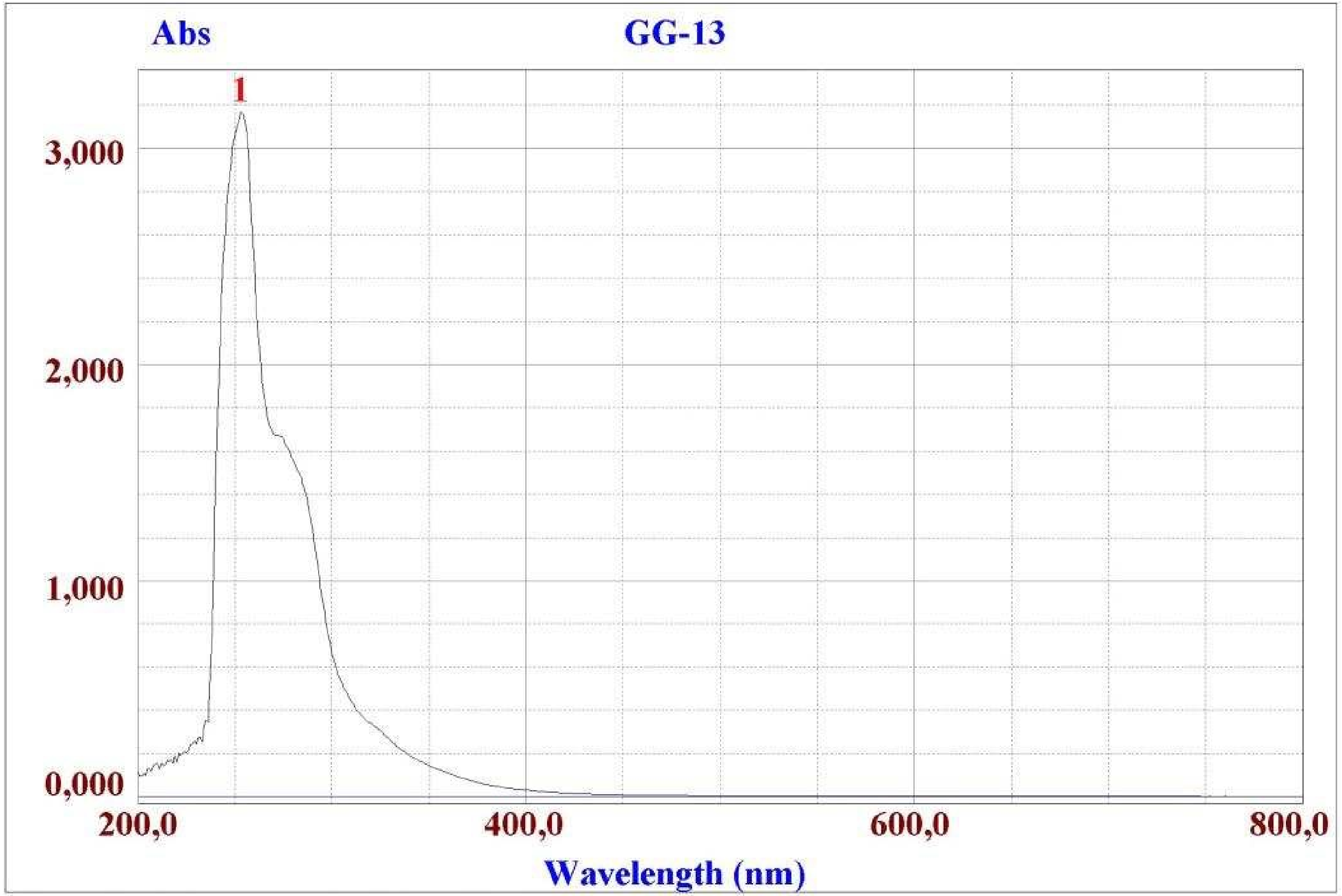

No.	Peak Type	Position	Height
1	Peak	253,4	3,169

Figure S41 UV spectrum of 3(R)-HEPE (2).

Acquisition Parameter

Source Type	ESI	Ion Polarity	Positive	Set Nebulizer	0.4 Bar
Focus	Not active	Set Capillary	3500 V	Set Dry Heater	200 °C
Scan Begin	50 m/z	Set End Plate Offset	-500 V	Set Dry Gas	4.0 l/min
Scan End	1500 m/z	Set Charging Voltage	2000 V	Set Divert Valve	Waste
		Set Corona	0 nA	Set APCI Heater	0 °C

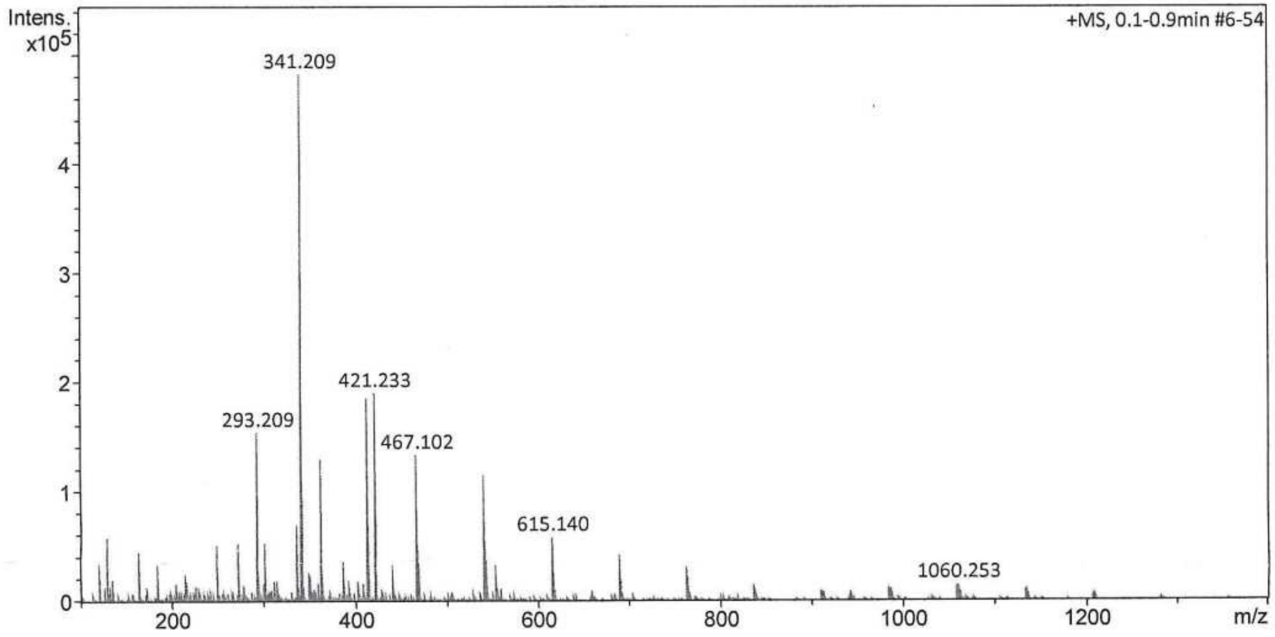

#	m/z	I %	#	m/z	I %
1	129.052	12.0	11	413.266	38.5
2	164.920	9.4	12	414.270	10.6
3	249.182	10.5	13	421.233	39.3
4	273.167	11.1	14	422.236	8.2
5	293.209	31.9	15	467.102	27.6
6	301.141	11.0	16	468.103	11.1
7	337.235	14.5	17	541.121	24.0
8	341.209	100.0	18	542.121	11.6
9	342.212	22.2	19	615.140	12.2
10	363.191	27.0	20	689.158	8.8

Figure S42 MS of 3(R)-HEPE (2)

Acquisition Parameter					
Source Type	ESI	Ion Polarity	Positive	Set Nebulizer	0.4 Bar
Focus	Not active	Set Capillary	3500 V	Set Dry Heater	200 °C
Scan Begin	50 m/z	Set End Plate Offset	-500 V	Set Dry Gas	4.0 l/min
Scan End	1500 m/z	Set Charging Voltage	2000 V	Set Divert Valve	Waste
		Set Corona	0 nA	Set APCI Heater	0 °C

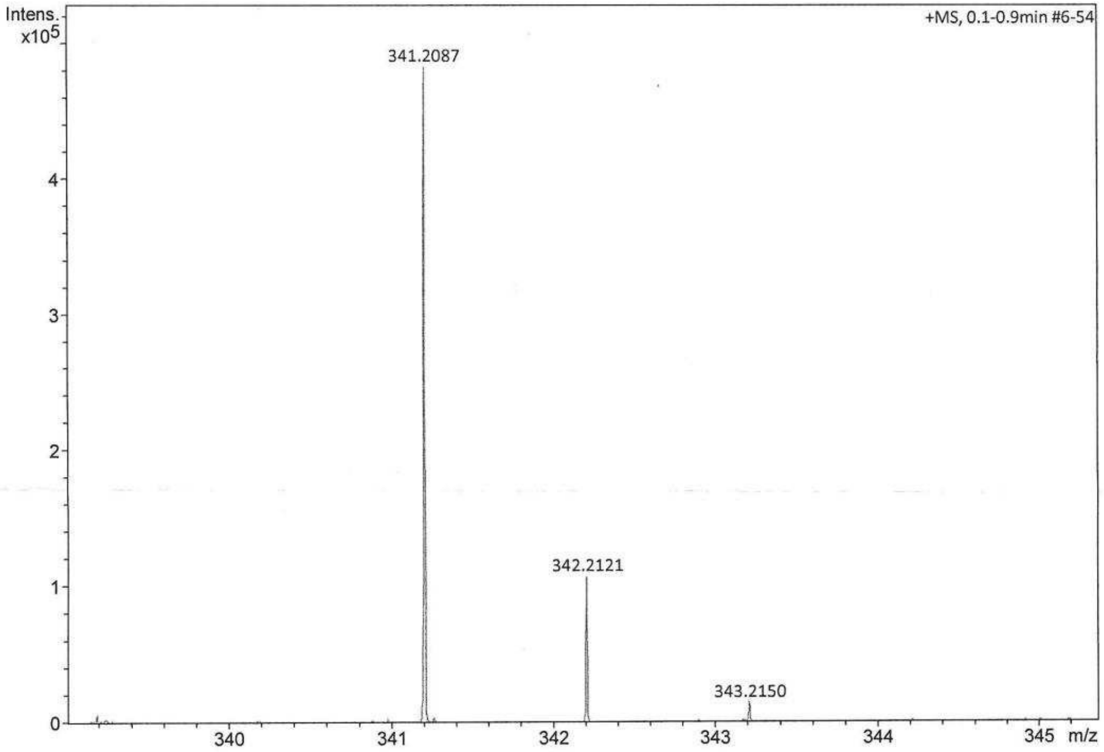

Meas. m/z	Ion Formula	m/z	err [ppm]
341.2087	C20H30NaO3	341.2087	0.1
	C18H25N6O	341.2084	-0.7
	C22H29O3	341.2111	7.1
	C16H26N6NaO	341.2060	-7.8
	C17H29N2O5	341.2071	-4.7

*Figure S43 HRMS of 3(R)-HEPE (**2**) ($C_{20}H_{30}O_3$)*

Aldol product 13:

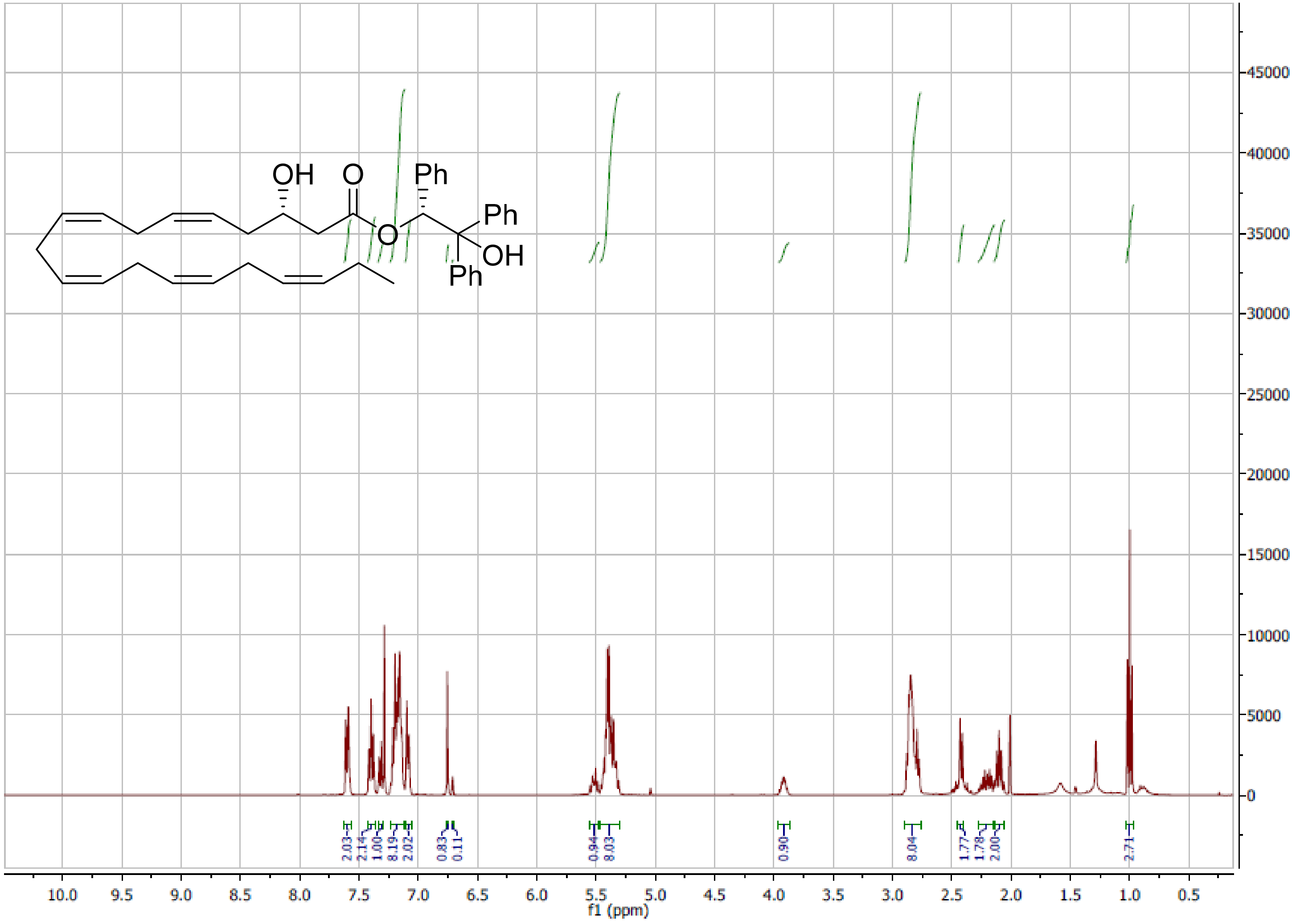

Figure S44. ^{1}H NMR spectrum of **14**.

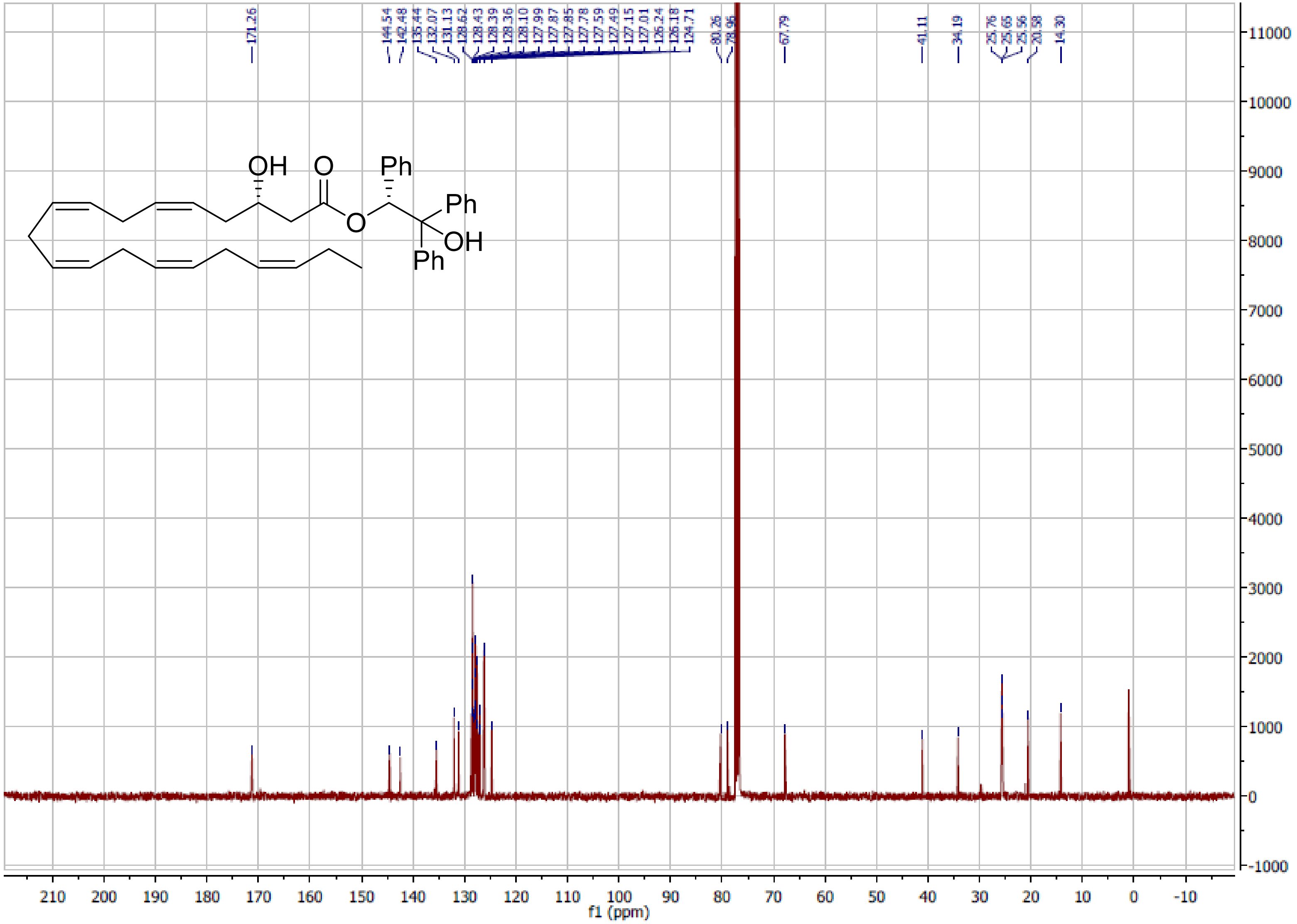

Figure S45. ^{13}C NMR spectrum of **14**.

(R)-4-isopropylthiazolidinone-2-thione **15:**

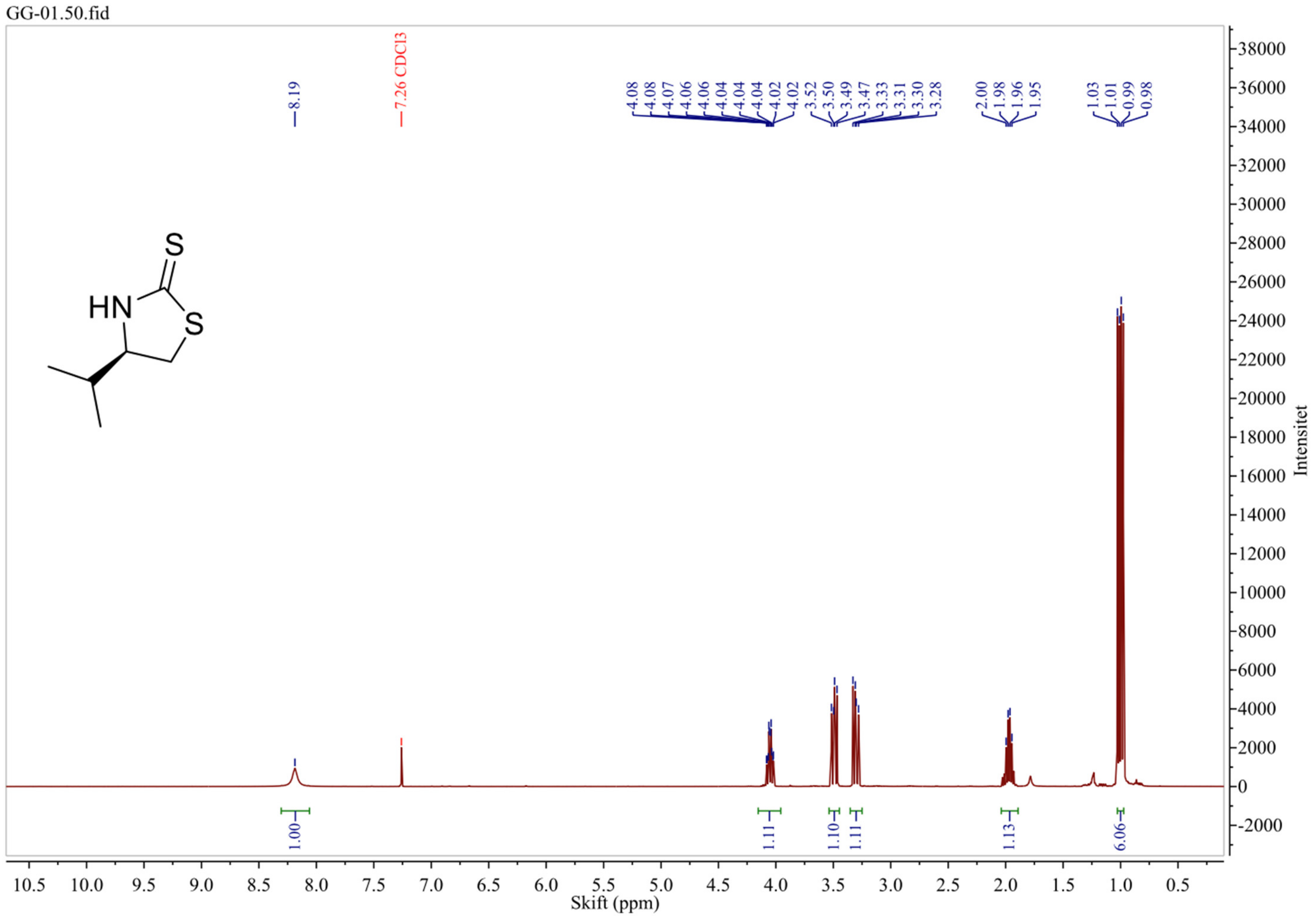

Figure S46 ^{1}H NMR spectrum of (R)-4-isopropylthiazolidinone-2-thione (15)

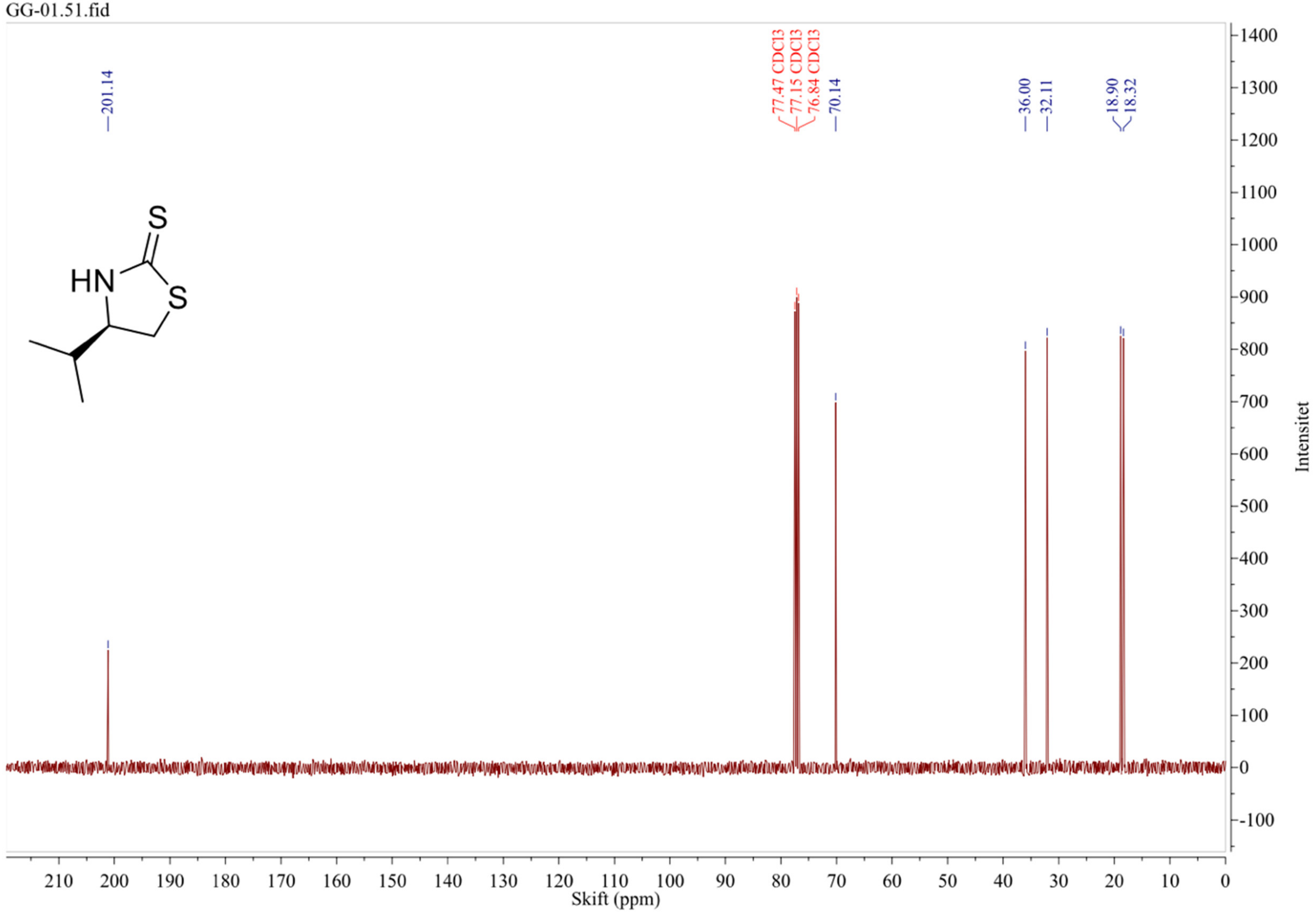

Figure S47 ^{13}C NMR spectrum of (R)-4-isopropylthiazolidinone-2-thione (15)

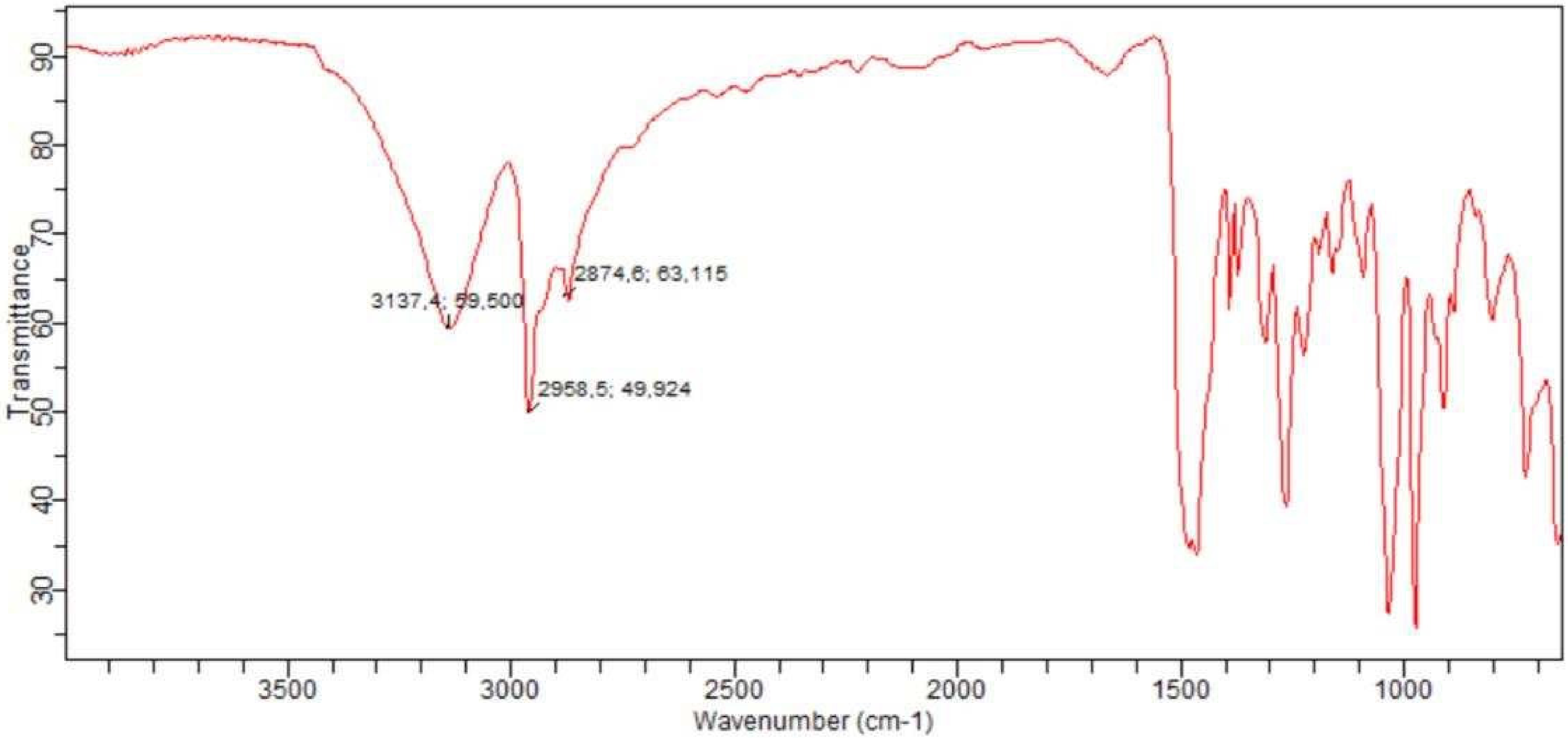

Figure S48 IR spectrum of (R)-4-isopropylthiazolidine-2-thione (15)

Ethyl ester 16:

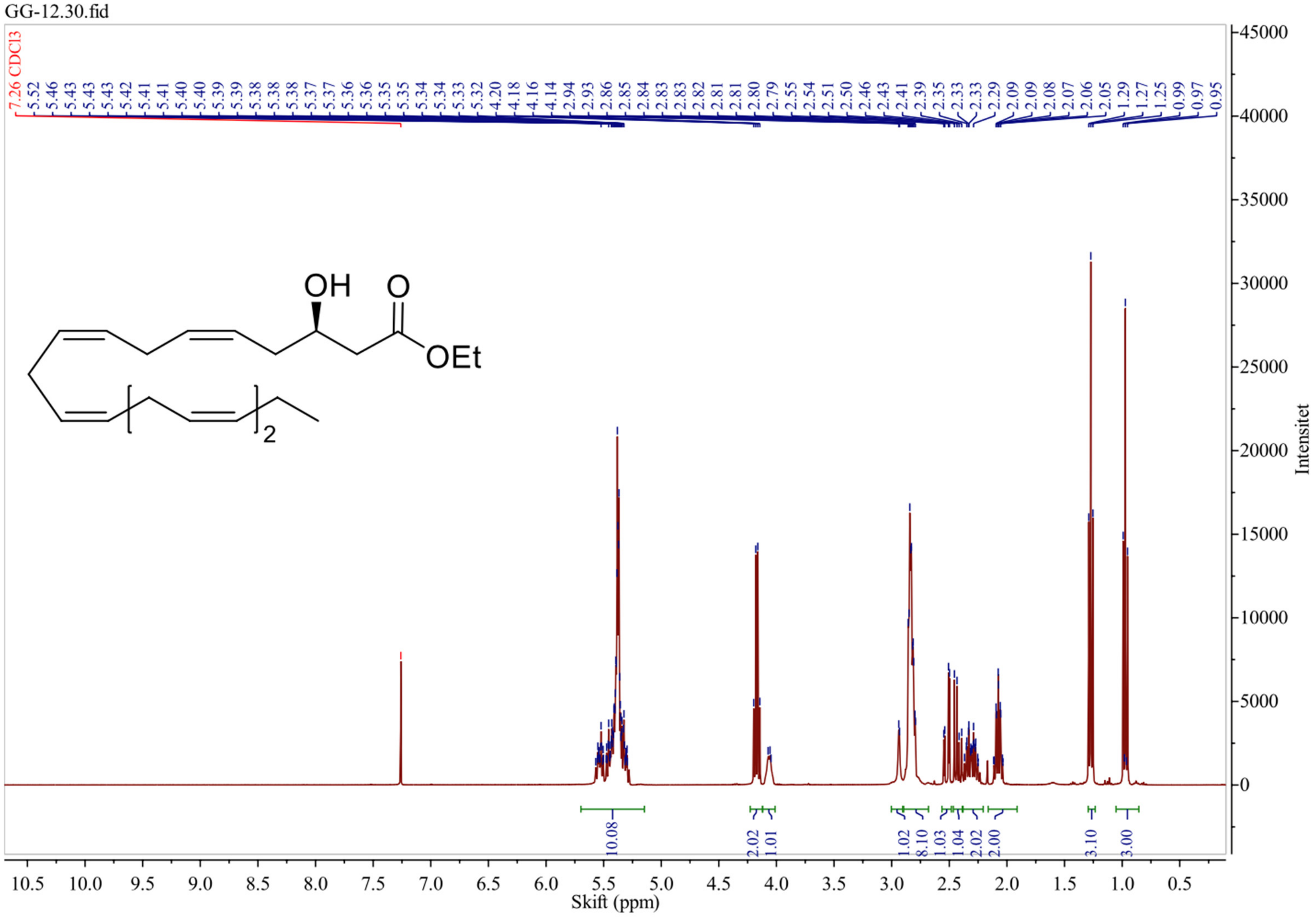

Figure S49 1H NMR spectrum of ethyl ester 16

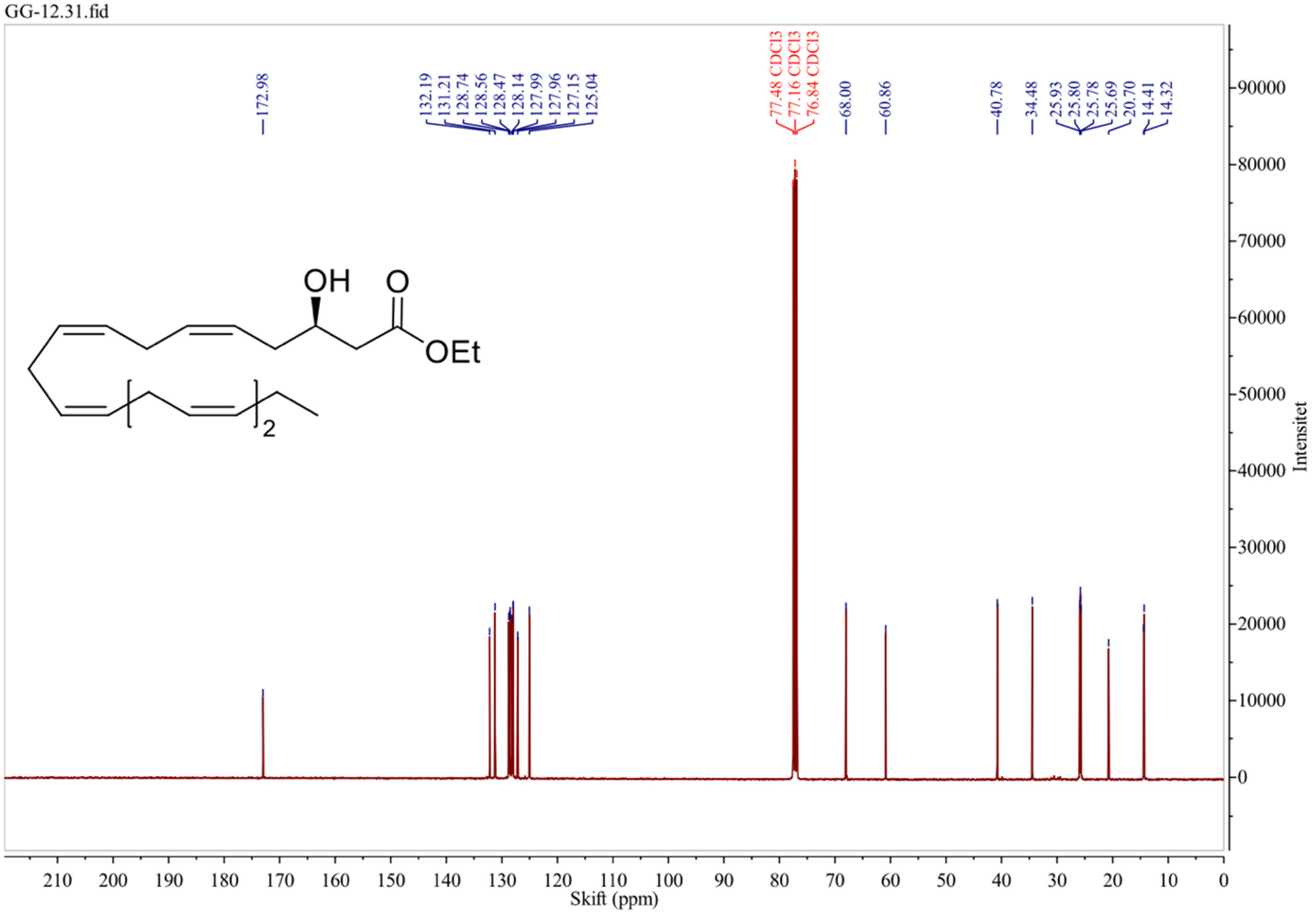

Figure S50 13C NMR spectrum of ethyl ester 16

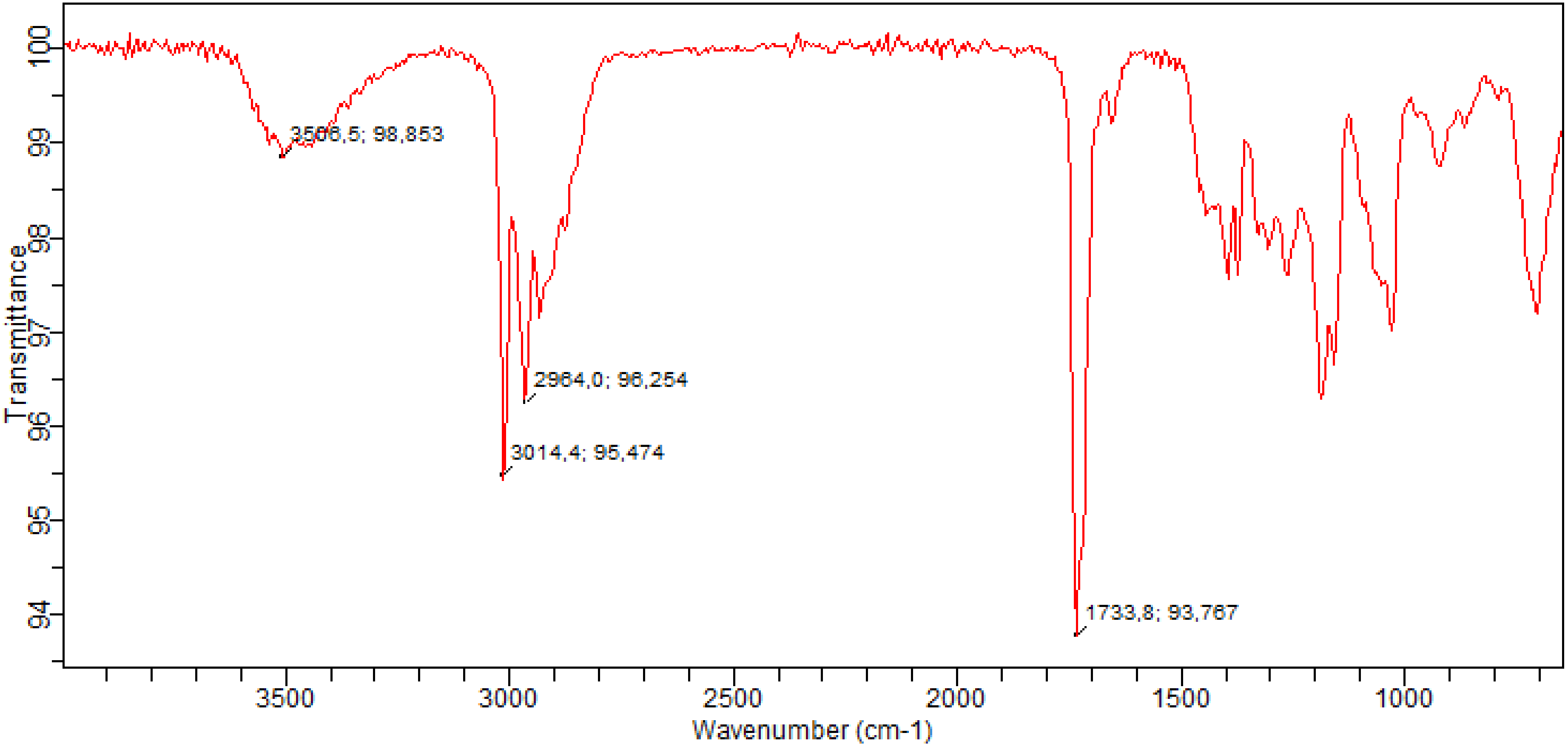

Figure S51 IR spectrum of ethyl ester 16.

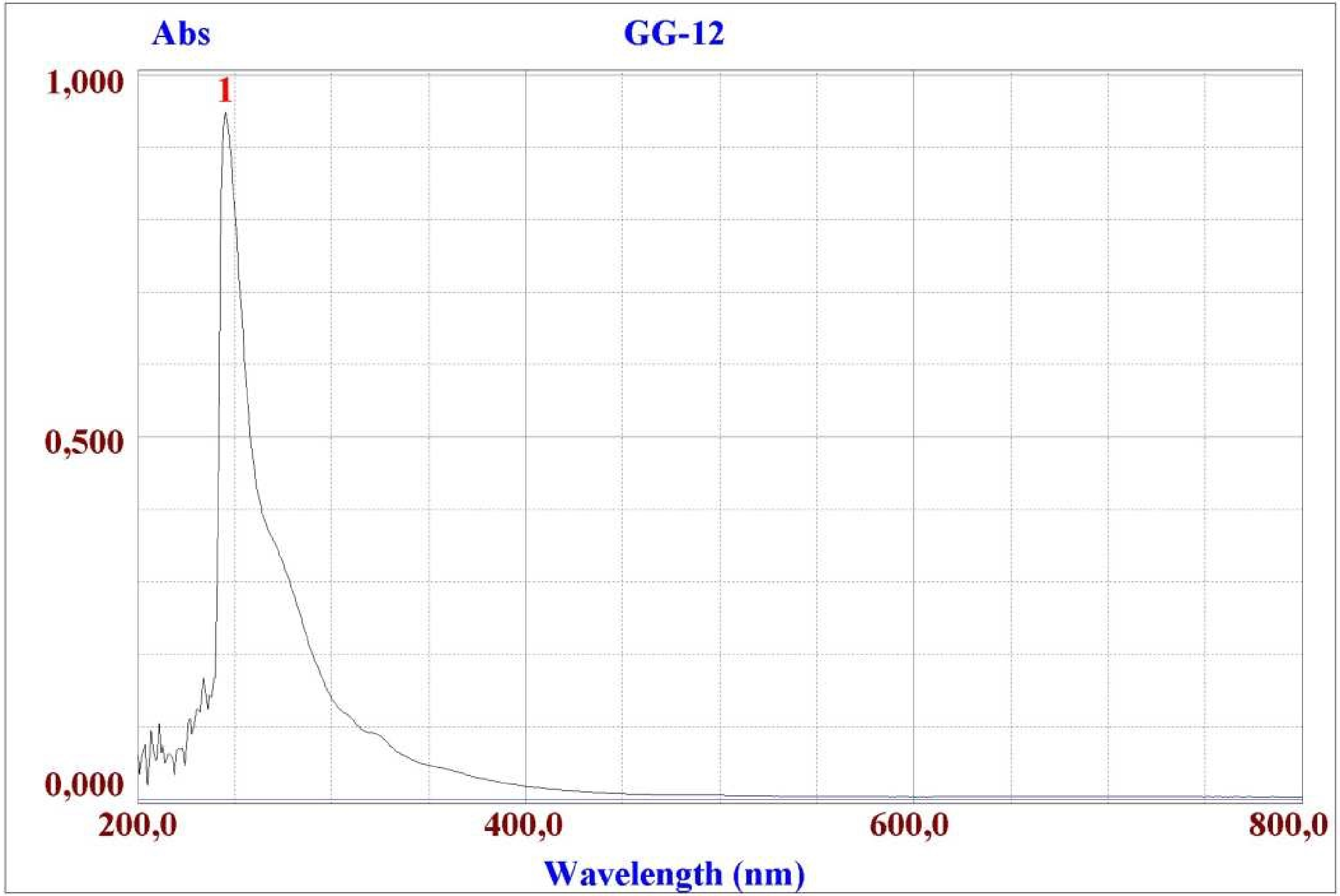

No.	Peak Type	Position	Height	Area	Start	End
1	Peak	245,3	0,949			

Figure S52 UV spectrum of ethyl ester 16.

Elemental Composition Report

Single Mass Analysis

Tolerance = 10.0 PPM / DBE: min = -1.5, max = 50.0
Isotope cluster parameters: Separation = 1.0 Abundance = 1.0%

Monoisotopic Mass, Odd and Even Electron Ions
31 formula(e) evaluated with 1 results within limits (up to 50 closest results for each mass)

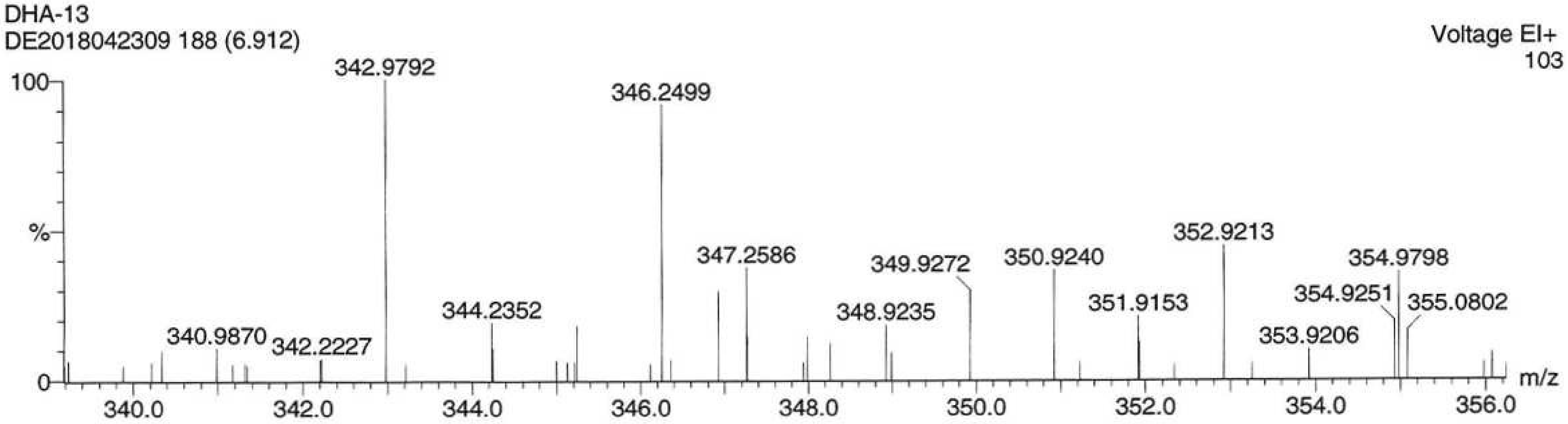

Minimum:				-1.5		
Maximum:		200.0	10.0	50.0		
Mass	Calc. Mass	mDa	PPM	DBE	Score	Formula
346.2499	346.2508	-0.9	-2.6	6.0	1	C22 H34 O3

Figure S53 HRMS of ethyl ester 16.

Mosher esters:

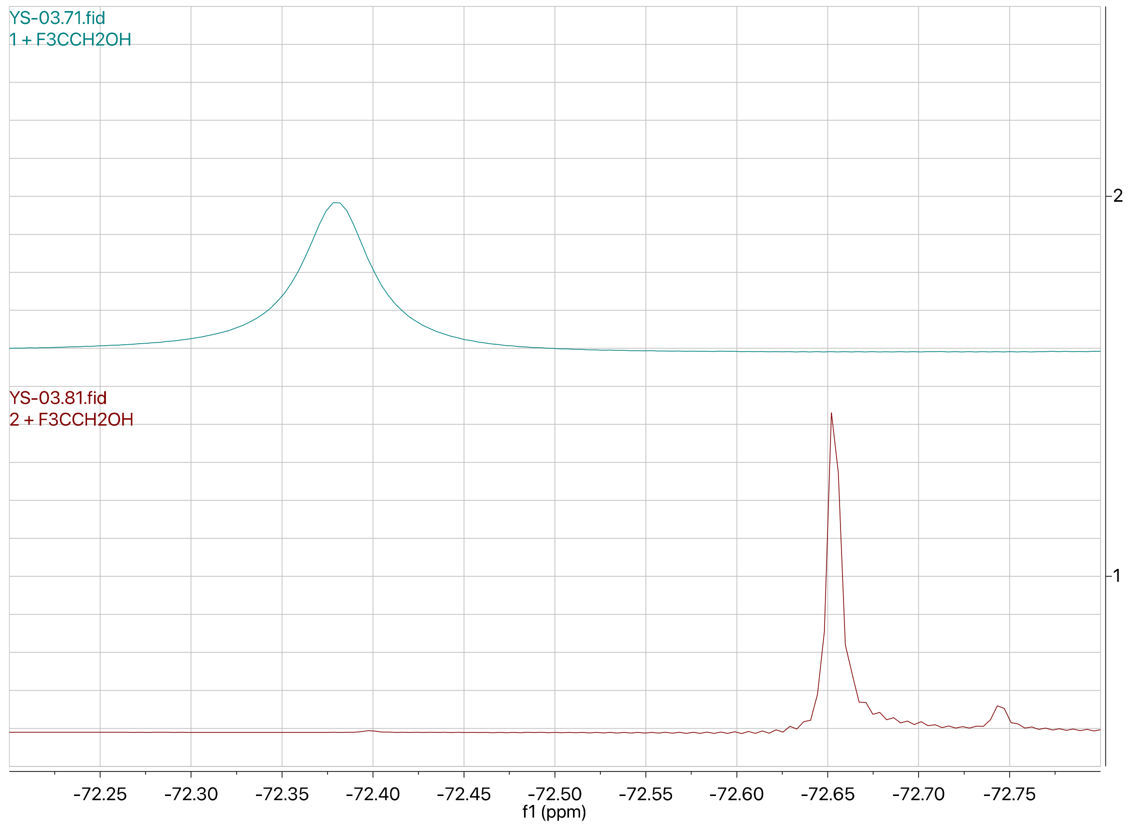

*Figure S54 ^{19}F NMR spectrum of assumed (S)-isomer of 15. Top is **15** reacted with (S)-MTPA (thus assumingly being S,S), while bottom being 15 reacted with (R)-MTPA (thus assumingly being S,R).*

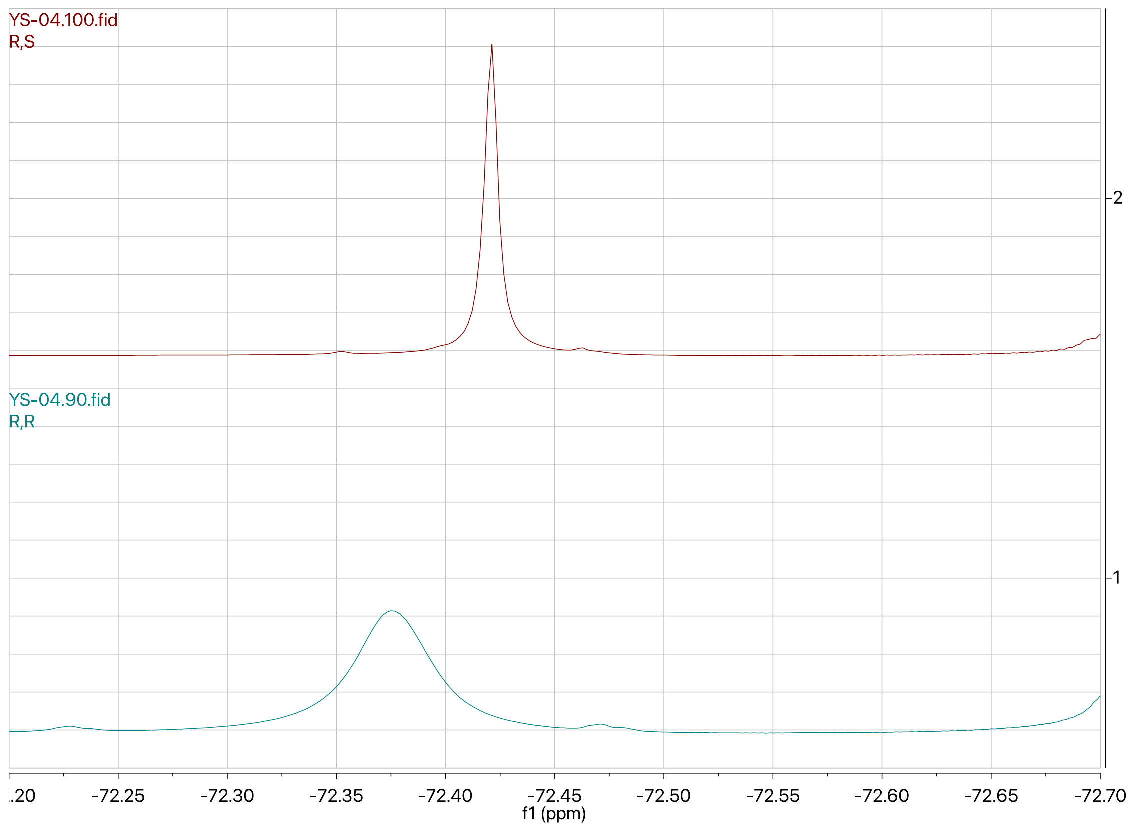

*Figure S55 ^{19}F NMR spectrum of assumed (R)-isomer of **15**. Top is 15 reacted with (S)-MTPA (thus assumingly being R,S), while bottom being 15 reacted with (R)-MTPA (thus assumingly being R,R).*
